# Ruthenium Single‐Atom Nanozyme Driven Sonosensitizer with Oxygen Vacancies Enhances Electron–Hole Separation Efficacy and Remodels Tumor Microenvironment for Sonodynamic‐Amplified Ferroptosis

**DOI:** 10.1002/advs.202416997

**Published:** 2025-04-25

**Authors:** Yang Zhu, Dengliang Wang, Chengzhong Du, Tiantian Wu, Penghui Wei, Hongjia Zheng, Guanting Li, ShunZhe Zheng, Lichao Su, Lingjun Yan, Yongrui Hu, Huimin Wang, Lisen Lin, Chenyu Ding, Xiaoyuan Chen

**Affiliations:** ^1^ Department of Neurosurgery Neurosurgery Research Institute The First Affiliated Hospital of Fujian Medical University Fuzhou Fujian 350209 P. R. China; ^2^ Department of Neurosurgery, National Regional Medical Center, Binhai Campus of First Affiliated Hospital Fujian Medical University Fuzhou Fujian 350212 P. R. China; ^3^ School of Pharmaceutical Sciences/NHC key laboratory of tropical disease control/School of Tropical Medicine Hainan Medical University Haikou 571199 P. R. China; ^4^ Departments of Diagnostic Radiology Surgery, Chemical and Biomolecular Engineering, and Biomedical Engineering Yong Loo Lin School of Medicine and College of Design and Engineering National University of Singapore Singapore 119074 Singapore; ^5^ Clinical Imaging Research Centre Centre for Translational Medicine Yong Loo Lin School of Medicine National University of Singapore Singapore 117599 Singapore; ^6^ Nanomedicine Translational Research Program Yong Loo Lin School of Medicine National University of Singapore Singapore 117597 Singapore

**Keywords:** ferroptosis, oxygen vacancy, single‐atom nanozyme, sonodynamic therapy, tumor microenvironment

## Abstract

Sonodynamic therapy (SDT) has emerged as a promising noninvasive approach for tumor therapy. However, the effectiveness of traditional inorganic semiconductor sonosensitizers is hindered by rapid electron (e^−^) and hole (h^+^) recombination under ultrasonic (US) stimulation, as well as the hypoxic and reductive conditions of tumor microenvironment (TME), which limit the generation of reactive oxygen species (ROS). Herein, a ruthenium (Ru) single‐atom nanozyme‐driven superimposition‐enhanced titanium dioxide‐based sonosensitizer (Ru/TiO_2‐x_ SAE) is presented that features sufficient oxygen vacancies and high e^−^/h^+^ separation efficiency. Through synchrotron radiation‐based X‐ray absorption spectroscopy and extended X‐ray absorption fine structure analysis it is confirmed that oxygen vacancies in TiO_2‐x_ nanoparticles promote the immobilization of single‐atomic Ru, forming Ru‐O₄ active sites. Density functional theory calculations demonstrate that oxygen vacancies alter the electronic structure of nanosensitizer, enhanced e^−^/h^+^ separation, increasing oxygen adsorption, and accelerating reaction kinetics under US stimulation, ultimately improving ROS production. Moreover, Ru/TiO_2‐x_ SAE boosts sonodynamic efficacy by mitigating the hypoxic and reductive TME. This is attributed to its catalase‐ and glutathione peroxidase 4‐like activities, which facilitate the generation of ROS and trigger lipid peroxidation‐mediated ferroptosis. These findings highlight the innovative role of single‐atom Ru in optimizing sonosensitizers for SDT‐induced ferroptosis, demonstrating its potential for advancing cancer therapy.

## Graphical abstract

An atomic‐level engineered ruthenium single‐atom nanozyme is fabricated that features sufficient oxygen vacancies and electron and hole separation efficiency, which enhances response to the sono‐suppressive tumor microenvironment and induces tumor ferroptosis. Density functional theory and experimental results demonstrate that Ru/TiO_2‐x_ SAE exhibits significant catalase‐ and glutathione oxidase‐mimicking activities, effectively alleviating hypoxia and depleting overproduced glutathione, thus initiating lipid peroxidation‐mediated ferroptosis.

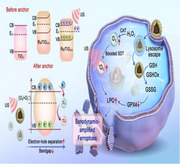

## 1 Introduction

Sonodynamic therapy (SDT), facilitated by ultrasound (US), has emerged as a prospective noninvasive tumor therapy owing to its superiorities in deeper tissue penetration and desirable spatial precision.^[^ ^1^ ^]^ SDT activates sonosensitizers with the US, leading to the generation of noxious reactive oxygen species (ROS) that trigger oxidative damage, which in turn effectively inhibits tumor growth.^[^ ^2^ ^]^ Sonosensitizers are crucial in strengthening the efficacy of SDT and have been widely utilized in cancer treatment.^[^ ^3^ ^]^ However, traditional inorganic sensitizers, such as titanium dioxide (TiO_2_) and zinc oxide nanoparticles (NPs), suffer from challenges due to their low production of ROS.^[^ ^4^ ^]^ This is mainly attributed to the limitations of inefficient ROS quantum yields and the tumor microenvironment (TME).^[^ ^5^ ^]^ On one side, TiO_2_ is hindered from the rapid recombination of electron–hole (e^−^/h^+^) pairs and its wide bandgap, which leads to suboptimal efficacy in SDT.^[^ ^1^, ^6^ ^]^ On the other side, the hypoxia prevalent in TME results in a deficiency of oxygen essential for ROS generation.^[^ ^7^ ^]^ Additionally, the ROS produced during SDT is quickly neutralized by the high levels of reductive glutathione (GSH) present in the TME, which significantly diminishes the effectiveness of SDT.^[^ ^8^ ^]^ Despite remarkable advancements in the development of various sonosensitizers, the precise engineering of sonosensitizers that can effectively enhance ROS generation efficiency, deplete the overproduced GSH, and alleviate the hypoxic TME remains a formidable challenge.

Due to the constraints of e^−^/h^+^ pair separation and the accelerated recombination of e^−^/h^+^ pairs in semiconductors with a wide bandgap, many nano‐sensitizers exhibit lower charge utilization and ROS quantum yields.^[^ ^9^ ^]^ In response to these challenges, a variety of strategies have been proposed, such as the incorporation of noble metals, the construction of Schottky heterojunctions, and the doping of heteroatoms.^[^ ^10^ ^]^ More importantly, defect engineering stands out as an effective strategy for modulating the surface properties of adsorbents.^[^ ^11^ ^]^ This approach not only enhances the separation efficiency of e^−^/h^+^ pairs but also amplifies oxygen adsorption and accelerates reaction kinetics.^[^ ^12^ ^]^ As previously reported, vacancies have the ability to alter the electronic structure of the nanosensitizer.^[^ ^13^ ^]^ This alteration directly influences the adsorption energy of reaction species and active sites, thereby enhancing the selectivity and kinetic properties of the sonosensitizer.^[^ ^14^ ^]^ Moreover, vacancies can selectively trap oxygen‐containing species and accelerate their electron transfer capabilities, which in turn significantly boosts the effectiveness of SDT.^[^ ^15^ ^]^ Thus, it is reasonable to anticipate that vacancies in sonosensitizers can effectively capture reaction species and reduce the adsorption energy, thereby amplifying the SDT performance.

Nanozyme, a category of nanomaterials endowed with inherent enzyme‐like activities, has opened the floodgates to artificial enzymes.^[^ ^16^ ^]^ Their appeal lies in the lower cost, tunable catalytic activity, and superior stability they offer, outperforming the attributes of their natural counterparts.^[^ ^17^ ^]^ Nanozymes have emerged as promising alternatives to natural enzymes, effectively bridging the unique intersection of nanotechnology and biomedicine.^[^ ^18^ ^]^ This convergence has opened up a vast spectrum of biomedical applications, ranging from in vitro diagnostics to in vivo therapeutic interventions. Despite the significant advancements in nanozyme engineering, challenges persist, including complex compositions and electronic structures, as well as suboptimal atomic utilization efficiency, which impedes their broader application.^[^ ^19^ ^]^ An emerging generation in nanozymes has arrived with the advent of single‐atom enzymes (SAEs), designed for biomedicine.^[^ ^20^ ^]^ SAEs boast well‐defined electronic structures, maximize atomic utilization efficiency, and exhibit unparalleled catalytic activity, positioning them as cutting‐edge solutions in the field of nanozymes.^[^ ^21^ ^]^ Significantly, the integration of SAEs into SDT systems holds the potential to synergistically amplify therapeutic efficacy. This is achieved by alleviating hypoxia and depleting high levels of reductive GSH within the TME, thereby initiating lipid peroxidation (LPO) and subsequently leading to the onset of irreversible ferroptosis. It is imperative to harness the full potential of SAEs to enhance SDT by remodeling the TME. However, to date, the exploration of SAEs as sonodynamic amplifiers has been relatively uncharted territory.

**Figure 1:**
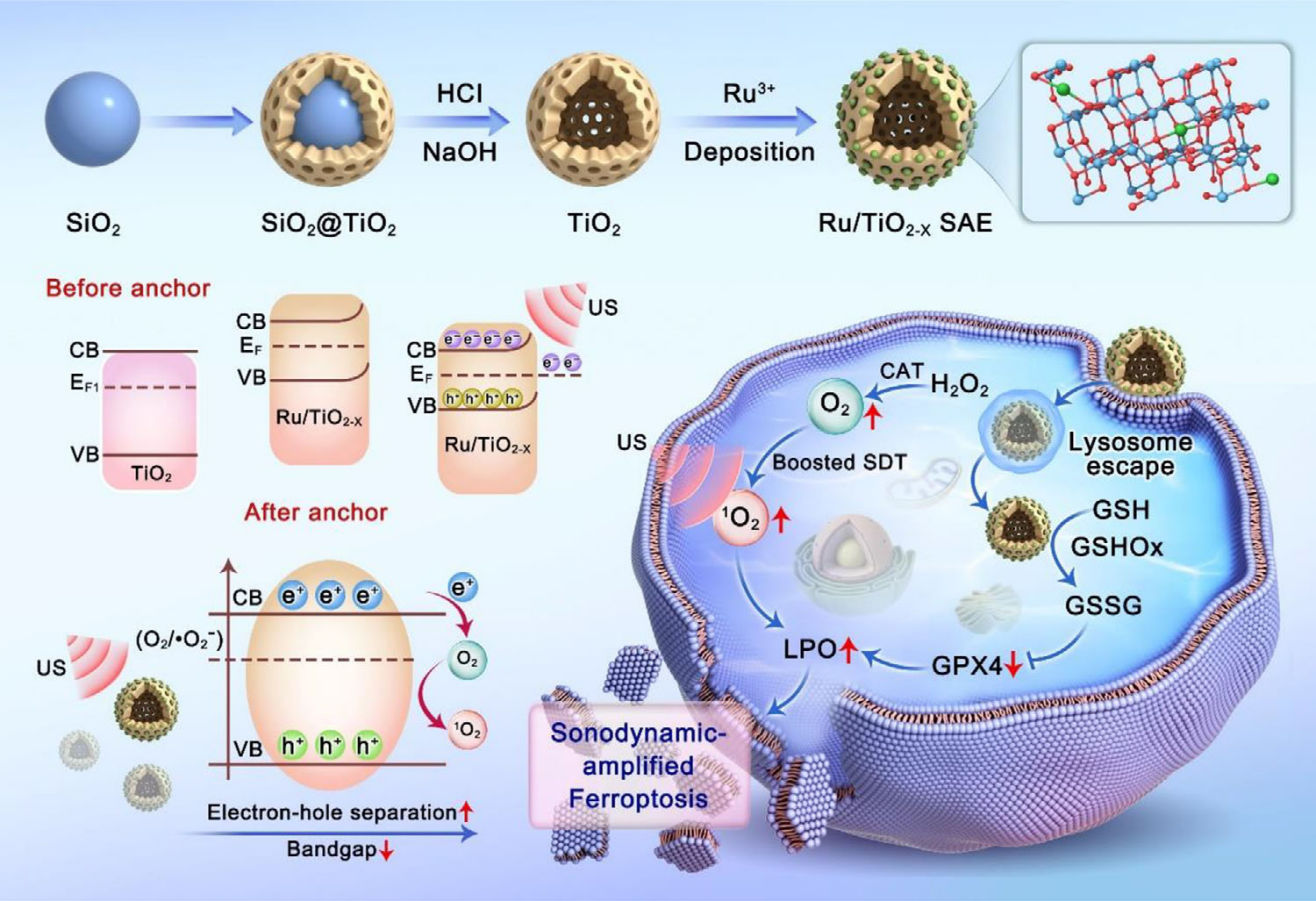
Schematic diagram of a sonodynamic‐amplified ferroptosis mechanism of Ru/TiO_2‐x_ SAE.

In this study, we report an atomic‐level engineered ruthenium (Ru) single‐atom nanozyme that features sufficient oxygen vacancies and high e^−^/h^+^ separation efficiency, which enhances response to the sono‐suppressive TME and induces tumor ferroptosis (**Figure** **1**). To demonstrate this concept, we utilize TiO_2‐x_ NPs with abundant oxygen vacancies to immobilize Ru‐O_4_ SAE (Ru/TiO_2‐x_ SAE), where positively charged Ru^𝛿+^ (0 < 𝛿^+^ < 3) single atoms anchor at oxygen vacancies coordinated with four oxygen atoms. The incorporation of abundant Ru^𝛿+^ single atoms, atomically dispersed within the TiO_2‐x_ NPs, significantly amplifies the e^−^/h^+^ separation. Compared to the traditional sonosensitizer like TiO_2_, Ru/TiO_2‐x_ SAE exhibits superior singlet oxygen (^1^O_2_) production efficiency due to its unique oxygen defects, which dramatically hinder the rate of electron–hole recombination and extend the lifetime of charge carriers. Furthermore, the sufficient Ru^𝛿+^ single atoms demonstrate significant catalase (CAT)‐mimicking activity, converting high levels of hydrogen peroxidation (H_2_O_2_) into sufficient oxygen, thereby alleviating hypoxia and strengthening response to the sono‐suppressive TME. On the other hand, Ru/TiO_2‐x_ SAE also exhibit glutathione oxidase (GSHOx)‐like catalytic performance, depleting overproduced reductive GSH to inhibit the expression of glutathione peroxidase 4 (GPX4) and trigger LPO‐mediated ferroptosis. Density functional theory (DFT) calculations and experimental results collectively elucidate the enhanced catalytic mechanism and superior therapeutic effect of Ru/TiO_2‐x_ SAE, which featured rich oxygen vacancies, in comparison to traditional TiO_2_ sonosensitizer. This study employs Ru SAE to optimize sonosensitizers, thereby enhancing response to the sono‐suppressive TME and advancing the progress of SDT.

## 2 Results and Discussions

### 2.1 Synthesis and Characterization of the Ru/TiO_2‐x_ SAE

**Figure 2:**
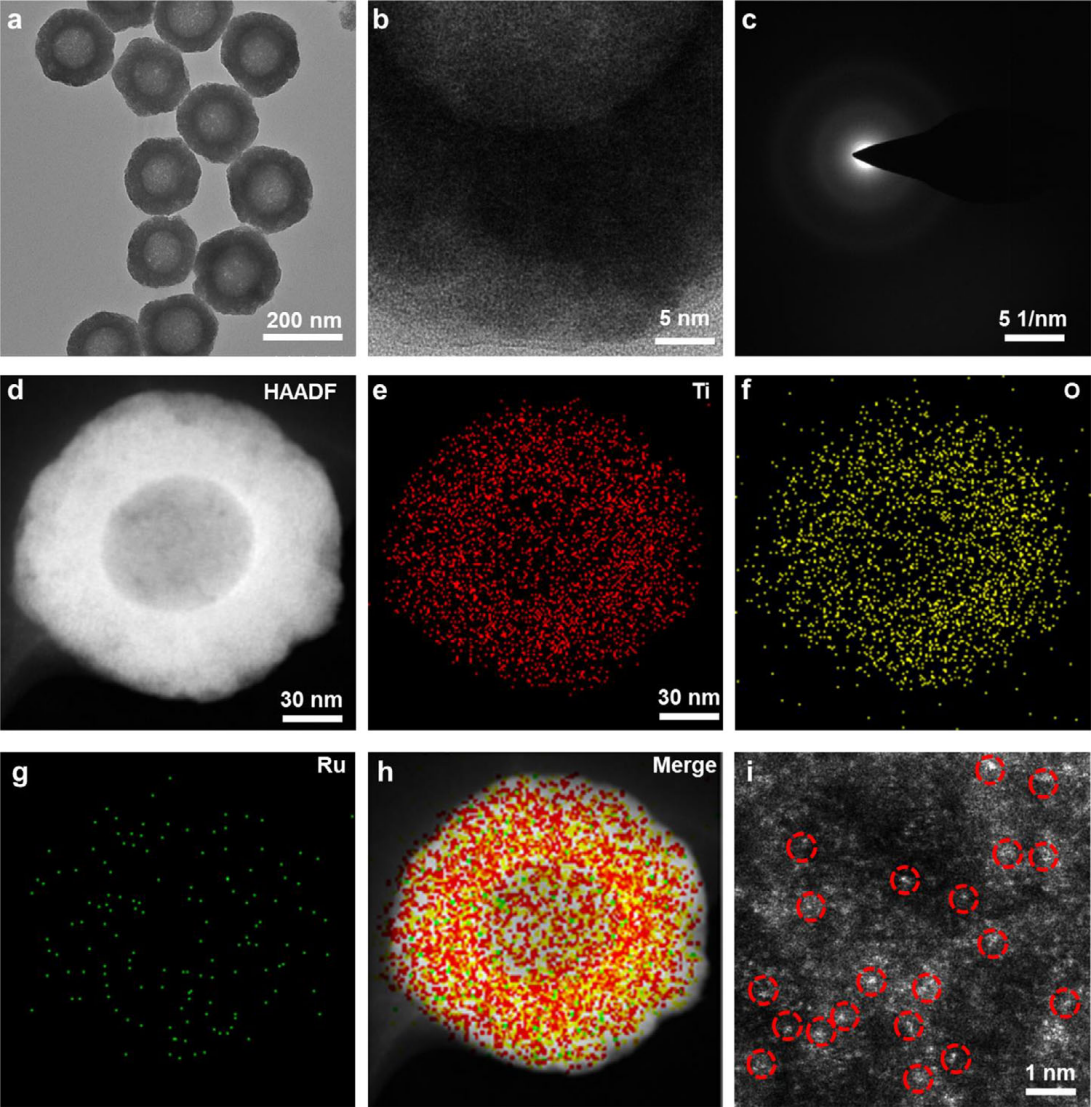
Morphology characterization of Ru/TiO_2‐x_ SAE. a) TEM and b) HRTEM images of Ru/TiO_2‐x_ SAE. c) SAED pattern of Ru/TiO_2‐x_ SAE. d–h) HAADFSTEM image and corresponding EDX elemental mapping images of Ru/TiO_2‐x_ SAE. g) AC HAADF‐STEM images of Ru/TiO_2‐x_ SAE. The single‐atomic Ru is marked with red circles.

The synthesis of TiO_2‐x_ supported atomically dispersed Ru is depicted in Figure 1. Initially, the silica NPs with a diameter of ≈100 nm were synthesized by using the Stöber method, as shown in Figure S1 (Supporting Information). Following this, TiO_2_ hollow mesoporous NPs were derived through an acid‐base etching process of SiO_2_@TiO_2_ NPs. As can be seen in Figure S2 (Supporting Information) the internal silica NPs were etched to form a hollow structural sphere, while the TiO_2_ shell was preserved, facilitating the deposition of metal atoms. Transmission electron microscopy (TEM) images revealed that TiO_2_ NPs possessed a characteristic hollow mesoporous spherical shape with a uniform particle size distribution, averaging ≈200 nm in diameter. Subsequently, Ru atoms were immobilized into the TiO_2_ NPs to form Ru@TiO_2_ NPs. The Ru/TiO_2‐x_ SAE was then synthesized by pyrolyzing at 400 °C in an atmosphere composed of 5% hydrogen and 95% argon. High‐resolution (HR) TEM images, as presented in **Figure** **2**a,b, demonstrated that the surface of Ru/TiO_2‐x_ SAE became rough and porous as a result of the high‐temperature carbonization process. The corresponding selected area electron diffraction (SAED) pattern of the Ru/TiO_2‐x_ SAE displayed distinct polycrystalline diffraction rings, indicative of anatase‐type crystals with high crystallinity (Figure 2c). The power X‐ray diffraction (XRD) pattern further confirmed the crystallographic nature of the Ru/TiO_2‐x_ SAE, exhibiting similar characteristic peaks (Figure S3, Supporting Information). Importantly, the absence of crystalline peaks for metallic Ru or Ru oxide nanoparticles in the XRD pattern suggested minimal Ru aggregation in the Ru/TiO_2‐x_ SAE, supporting the potential for the creation of atomically dispersed Ru SAE. Energy‐dispersive X‐ray spectroscopy (EDX) mapping revealed a homogeneous distribution of Ru throughout the Ru/TiO_2‐x_ SAE structure (Figure 2d–h). The EDX spectrum confirmed that Ru atoms were anchored within the TiO_2‐x_ framework (Figure S4, Supporting Information). The loading efficiency of the Ru atom in the Ru/TiO_2‐x_ SAE was determined to be 0.68% using inductively coupled plasma mass spectrometry (ICP‐MS). Direct observation via aberration‐corrected atomic‐resolution high‐angle annular dark‐field scanning transmission electron microscopy (AC HAADF‐STEM) revealed numerous bright spots, highlighted with red circles, which confirmed the presence of single‐atomic Ru on oxygen vacancies (Figure 2i). We have investigated the stability of Ru/TiO_2‐x_ SAE in DMEM and blood samples. As shown in Figure S5 (Supporting Information), the DLS result showed that the diameter of Ru/TiO_2‐x_ SAE remained stable for 96 h, which indicated the stability of Ru/TiO_2‐x_ SAE in DMEM and blood samples. In addition, we also have investigated the zeta potentials of both Ru/TiO_2‐x_ SAE and TiO_2_ NPs (Figure S6, Supporting Information). These findings suggested the successful construction of single‐atom Ru species anchored on TiO_2‐x_ NPs.

**Figure 3:**
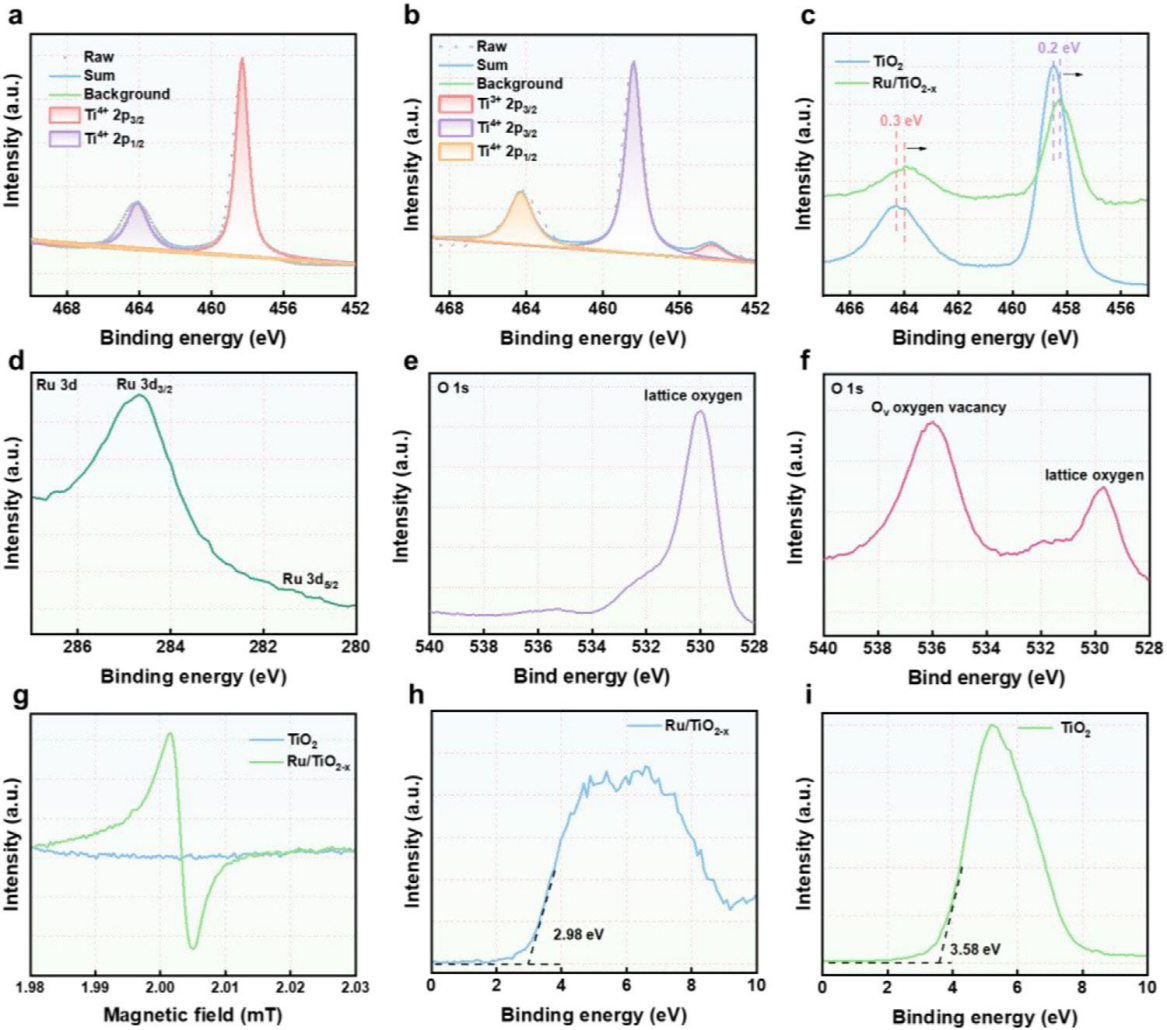
Effect of oxygen vacancies and Ru deposition on Ru/TiO_2‐x_ SAE. High‐resolution Ti 2p XPS in a) TiO_2_ NPs and b) Ru/TiO_2‐x_ SAE. c) Ti 2p XPS in TiO_2_ NPs and Ru/TiO_2‐x_ SAE. d) Ru 3d XPS in Ru/TiO_2‐x_ SAE. e) O 1s XPS in TiO_2_ NPs and f) Ru/TiO_2‐x_ SAE. g) EPR spectra of TiO_2_ NPs and Ru/TiO_2‐x_ SAE. The valence band of h) Ru/TiO_2‐x_ SAE and i) TiO2 NPs.

To investigate the binding states of Ru and oxygen atoms in Ru/TiO_2‐x_ SAE, X‐ray photoelectron spectroscopy (XPS) analysis was performed (Figure S7, Supporting Information). As shown in **Figure** **3**a,b, the high‐resolution Ti 2p spectrum of Ru/TiO_2‐x_ SAE clearly showed the presence of reduced Ti^3+^ ions alongside the native T^i4+^ ions, in contrast to TiO_2_ NPs, due to the reduction by hydrogen at high temperature. The increased presence of lower oxidation state Ti species is prone to lose its electrons, leading to heightened oxidation as a result of a diminished electron density cloud. As observed in the Ti 2p XPS patterns of Ru/TiO_2‐x_ SAE, two peaks are visible at binding energies of 458.1 and 463.8 eV, corresponding to the spin‐orbital components of Ti 2p_3/2_ and Ti 2p_1/2_, respectively. These peaks are shifted by 0.2 and 0.3 eV lower compared to TiO_2_ NPs (Figure 3c), indicating an interaction between TiO_2‐x_ and Ru single atoms. In addition, the Ru 3d profile demonstrated that the Ru 3d_3/2_ binding energy at 284.4 eV was situated between that of metallic Ru^0^ (281.4 eV) and oxidation state of Ru^3+^ (285.2 eV), suggesting the Ru^δ+^ (0 < δ < 3) nature of Ru species within TiO_2‐x_ (Figure 3d). Furthermore, the increase in lower oxidation state Ti species is directly linked to the introduction of oxygen vacancies. To verify the presence of oxygen vacancies in Ru/TiO_2‐x_ SAE, XPS and electron paramagnetic resonance (EPR) were employed to characterize the electronic structure. Compared to the O 1s XPS spectrum of TiO_2_ NPs, Ru/TiO_2‐x_ SAE presented two peaks at a binding energy of 536.0 and 529.7 eV, corresponding to oxygen vacancies and lattice oxygen, respectively (Figure 3e,f). Moreover, the Ru/TiO_2‐x_ SAE sample showed a distinct EPR signal at a g value of 2.003, which is attributed to electrons trapped in oxygen vacancies (Figure 3g,h). Notably, the proportion of defect oxygen was higher than that of lattice oxygen, which not only reduced the adsorption energy but also acted as a conduit for electron storage and transport, enhancing electron transfer and strengthening SDT efficiency. The bandgap energy was determined using the Kubelka–Munk function, corresponding to the *X*‐intercept of the extrapolated linear portion of the plot. The bandgap of TiO_2_ NPs and Ru/TiO_2‐x_ SAE was 3.58 and 2.98 eV, respectively (Figure 3i), implying that the anchoring of single‐atomic Ru resulted in a narrower bandgap for Ru/TiO_2‐x_ SAE. These findings indicated the successful fabrication of Ru/TiO_2‐x_ SAE with sufficient oxygen vacancies and a narrower bandgap, which effectively trapped oxygen‐containing species and accelerated e^−^/h^+^ separation.

**Figure 4:**
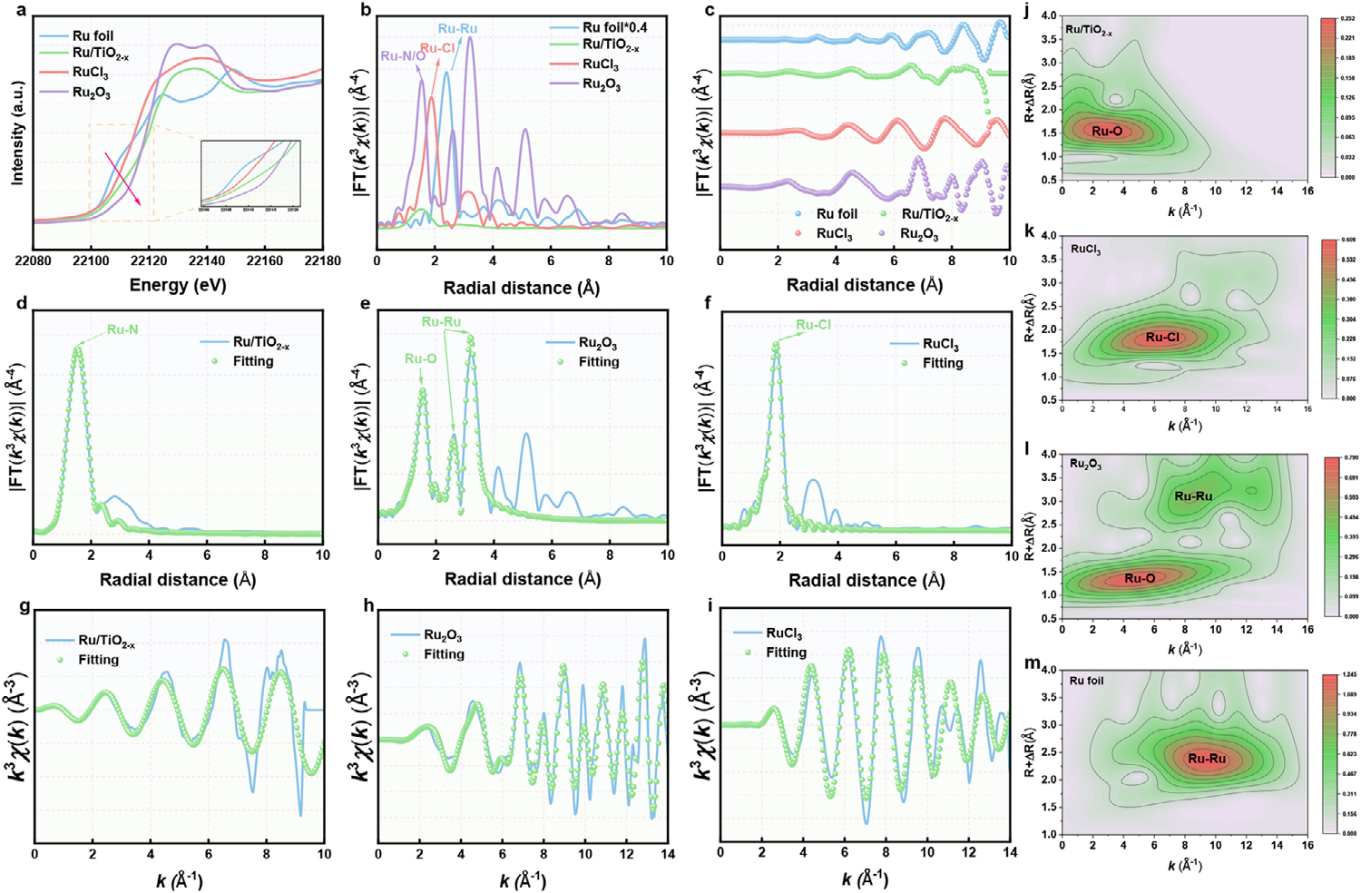
Atomic structural characterization of Ru/TiO_2‐x_ SAE. a) Ru K‐edge XANES spectra and the enlarged pre‐edge region (inset)of Ru foil, Ru/TiO_2‐x_ SAE, RuCl_3_, and Ru_2_O_3_. b) The Fourier transform EXAFS of the Fe K‐edge of Ru foil, Ru/TiO_2‐x_ SAE, RuCl_3_, and Ru_2_O_3_. c) EXAFS curves of Ru foil, Ru/TiO_2‐x_ SAE, RuCl_3_, and Ru_2_O_3_ at the k space. EXAFS fitting curve of d) Ru/TiO_2‐x_ SAE, e) Ru_2_O_3_, and f) RuCl_3_ at the R space. EXAFS fitting curve of g) Ru/TiO_2‐x_ SAE, h) Ru_2_O_3_, and i) RuCl_3_ at the k space. Wavelet transformation of Fe K‐edge EXAFS of j) Ru/TiO_2‐x_ SAE, k) RuCl_3_, l) Ru_2_O_3_, m) Ru foil at the k space.

To further analyze the electronic structure and coordination environment of the Ru atom within Ru/TiO_2‐x_ SAE, synchrotron radiation‐based X‐ray absorption near‐edge structure (XANES) spectroscopy and extended X‐ray absorption fine structure (EXAFS) were conducted. The Ru K‐edge XANES spectra revealed that the energy absorption threshold of Ru/TiO_2‐x_ SAE was situated between those of Ru foil and Ru_2_O_3_ (**Figure** **4**a). This placement suggested an oxidation state of Ru^δ+^ (0 < δ < 3) in Ru/TiO_2‐x_ SAE, consistent with the XPS results. In addition, the Fourier transform EXAFS at the R‐space spectrum showed that Ru/TiO_2‐x_ SAE showed a clear peak at 1.47 Å corresponding to the Ru─O bond, while lacking noticeable peaks at 2.36 Å for the Ru─Ru bond, thereby confirming the atomically dispersed Ru active sites in both Ru/TiO_2‐x_ SAE (Figure 4b–f; Figure S8, Supporting Information). The EXAFS fitting analysis at the Ru K‐edge provided insights into the coordination numbers and structure parameters surrounding the single‐atomic Ru species. As depicted in Figure 4g–i, Figure S9, and Table S1 (Supporting Information), the analysis revealed that Ru atoms in Ru/TiO_2‐x_ SAE were coordinated with approximately four O atoms. Furthermore, wavelet transform (WT) analysis was carried out on Ru/TiO_2‐x_, RuCl_3_, Ru_2_O_3_, and Ru foil to further confirm the presence of single‐atomic Ru species on vacancies TiO_2‐x_. As expected, Ru/TiO_2‐x_ SAE displayed a WT signal at 4.0 Å^−1^ corresponding to the Ru─O bond, with no WT intensity related to Ru─Ru bonds observed (Figure 4j–m). These findings decisively indicated the successful fabrication of Ru/TiO_2‐x_ SAE with atomically dispersed Ru‐O_x_ sites.

### 2.2 Catalytic Activities of Ru/TiO_2‐x_ SAE

**Figure 5:**
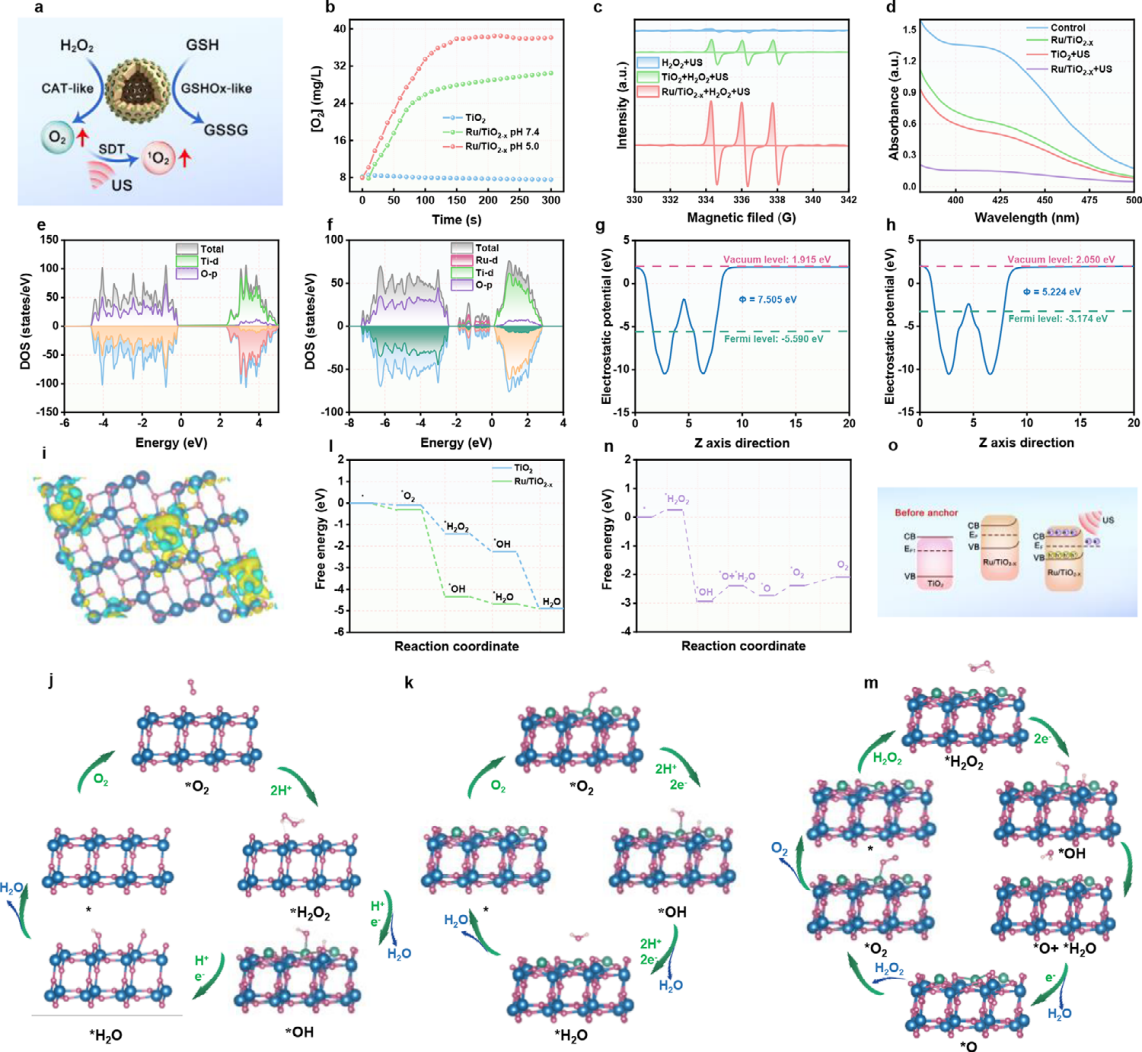
DFT calculations of the amplification of the SDT efficacy and CAT‐like activity of Ru/TiO_2‐x_ SAE. a) Schematic diagram of CAT‐ and GSHOx‐like activities of Ru/TiO_2‐x_ SAE. b) The oxygen generation rate of H_2_O_2_ catalyzed by Ru/TiO_2‐x_ SAE under different pH conditions was determined by a dissolved oxygen analyzer. c) EPR spectra of different formulations plus H_2_O_2_ with TEMP as the spin trapper. d) GSH depletion capacity of different nanozymes using a DTNB kit. The total DOS spectra of e) TiO_2_ NPs and f) Ru/TiO_2‐x_ SAE. The work functions spectra of g) TiO_2_ NPs and h) Ru/TiO_2‐x_ SAE. i) The local charge density difference plot of the Ru/TiO_2‐x_ SAE. Yellow: charge accumulation; Cyan: charge depletion. The isosurface value is set to 0.005 e/Bohr^3^. DFT optimized structures of the oxygen reduction reaction (ORR) intermediates on j) TiO_2_ NPs and k) Ru/TiO_2‐x_ SAE surface. l) Gibbs free‐energy diagrams for different pathways of ORR on the modeled surface of TiO_2_ NPs and Ru/TiO_2‐x_ SAE. m) DFT optimized structures of H_2_O_2_ decomposition to O_2_ on Ru/TiO_2‐x_ SAE. n) Gibbs free‐energy diagrams of H_2_O_2_ decomposition to O_2_ on Ru/TiO_2‐x_ SAE. o) Boosted mechanism of Ru/TiO_2‐x_ SAE for SDT.

After characterizing the coordination number of Ru atoms in Ru/TiO_2‐x_ SAE, we systematically evaluated its enzyme‐like activities, including CAT‐ and GSHOx‐like activities, under mild acidic conditions that simulate the TME (**Figure** **5**a). The CAT‐mimicking activity was assessed by determining oxygen generation from the catalytic disproportionation of H_2_O_2_ using a dissolved oxygen analyzer. Figure 5b shows that Ru/TiO_2‐x_ SAE presented superior CAT‐like activity under mild acidic conditions compared to natural pH. In contrast, TiO_2_ NPs exhibited negligible catalytic performance, highlighting the dominating role of Ru single atoms in CAT mimicry. In addition, we have measured the CAT‐like activity of different nanozymes. As can be seen in Figure S10 (Supporting Information), the Ru/TiO_2‐x_ SAE exhibited the highest CAT‐like activity compared to reported nanozymes. Furthermore, we used EPR to assess the SDT efficacy of both TiO_2_ NPs and Ru/TiO_2‐x_ SAE with the spin trapper 2,2,6,6‐tetramethylpiperidine (TEMP). As depicted in Figure 5c, the characteristic 1:1:1 triplet peak confirmed the generation of ^1^O_2_ by TiO_2_ NPs under US. In contrast, Ru/TiO_2‐x_ SAE produced a significantly higher ^1^O_2_ generation efficacy, attributed to the abundant oxygen supplied by CAT‐like activity and highly enhanced e^−^/h^+^ separation due to oxygen vacancies. Furthermore, fluorescence spectrophotometer analysis was employed to measure charge separation efficiency under ultrasonic stimulation. As shown in Figure S11 (Supporting Information), the Ru/TiO_2‐x_ SAE combined with US exhibited higher charge separation efficiency compared to TiO_2_ NPs. Since Ru^δ+^ can catalyze the oxidation of the thiol group in GSH, we evaluated the GSHOx‐like activity of Ru/TiO_2‐x_ SAE using 5,5′‐dithiobis(2‐nitrobenzoic acid) (DTNB) as a probe (Figure 5d). As expected, Ru/TiO_2‐x_ SAE demonstrated impressive GSH depletion capacity due to the reaction of single‐atomic Ru with the thiol group in GSH. Importantly, Ru/TiO_2‐x_ SAE combined with the US presented superior GSH consumption compared to TiO_2_ NPs with the US, which was attributed to the high e^−^/h^+^ separation by oxygen vacancies and the extended lifetime of charge carriers. Furthermore, we have investigated the influence of pH and temperature on the catalytic activity of Ru/TiO_2‐x_ SAE and TiO_2_ NPs (Figures S12 and S13, Supporting Information). These findings revealed that Ru/TiO_2‐x_ SAE amplified the SDT efficacy by improving the generation of ^1^O_2_ efficacy and GSH depletion capacity, owing to the presence of oxygen vacancies and sufficient single‐atomic Ru in Ru/TiO_2‐x_ SAE.

### 2.3 DFT Theoretical Simulations

To further disclose the mechanism behind the boosted catalytic performances of Ru/TiO_2‐x_ SAE induced by oxygen vacancies, we carried out density functional theory (DFT) calculations. These nanostructures were modeled and simulated using a defect crystal approximation representation. The geometrically optimized structural diagrams of TiO_2_ NPs and Ru/TiO_2‐x_ SAE are presented in Figures S14 and S15 (Supporting Information). For Ru/TiO_2‐x_ SAE, we observed a decrease in the O‐Ti‐O bond angle and an increase in the Ti─O bond length following the deposition of single‐atom Ru compared to TiO_2_ NPs. This suggested that the formation of oxygen vacancies resulted in the lattice distortion of Ru/TiO_2‐x_ SAE. By analyzing the total density of states (DOS) for TiO_2_ NPs (Figure 5e) and Ru/TiO_2‐x_ SAE (Figure 5f), we identified a defect band between the conduction and valence bands for Ru/TiO_2‐x_ SAE. This facilitates easy excitation of electrons into the conduction band, allowing them to migrate to the surface and participate in reactions that accelerate the separation of e^−^/h^+^ pairs under US irradiation. Furthermore, the DOS pattern of Ru/TiO_2‐x_ SAE revealed interwoven Ti and single‐atomic Ru, indicating that Ru is anchored to oxygen via vacancies, consistent with our XPS results. We calculated the work functions of TiO_2_ and Ru/TiO_2‐x_ SAE, along with the charge density difference, to elucidate the interface characteristics and charge distribution between TiO_2‐x_ and single‐atomic Ru. The work functions were found to be 7.505 eV for TiO_2_ NPs and 5.224 eV for Ru/TiO_2‐x_ SAE (Figure 5g,h). The substantial difference in work functions resulted in strong interface activation behavior, promoting the spontaneous flow of electrons from TiO_2‐x_ to single‐atomic Ru. This process reaches a new equilibrium at the Fermi level, causing the band of TiO_2‐x_ to bend upward and form a lower barrier at the interface. The charge density difference pattern and the interface side view of the Ru/TiO_2‐x_ SAE contact further illustrated the charge distribution between TiO_2‐x_ and Ru (Figure 5i; Figure S16, Supporting Information), confirming the strong interaction at the interface between single‐atomic Ru and TiO_2‐x_, in agreement with XPS results. The significant charge transfer from TiO_2‐x_ to Ru showed that the construction of the built‐in electric field enhanced effective electron separation. We proposed a reaction pathway for optimizing the reduction of adsorbed O_2_ to H_2_O by optimizing the surface intermediate structures of TiO_2_ and Ru/TiO_2‐x_ SAE using DFT. The five proton–electron transfer steps of TiO_2_ NPs surface sites were as follows: * + O_2_ → *O_2_ + 2H^+^ + 2e^−^ → H_2_O_2_* + H^+^ + e^−^ → *OH + H^+^ +e^−^ → H_2_O* → H_2_O + * (Figure 5j). For the Ru/TiO_2‐x_ SAE surface, the four proton‐electron transfer steps are: * + O_2_ → *O_2_ + 2H^+^ + 2e^−^ → 2 *OH + 2H^+^ + 2e^−^ → H_2_O* → H_2_O + *(Figure 5k). According to the Gibbs (ΔG) free energy pattern (Figure 5l), the deprotonation steps occurred more readily on the Ru/TiO_2‐x_ SAE surface compared to TiO_2_ NPs. These results revealed that Ru/TiO_2‐x_ SAE boosted oxygen utilization by accelerating oxygen adsorption and reaction kinetics, which is particularly beneficial for SDT. In addition, Figure 5m,n depicted the primary intermediate structures and Gibbs free energy diagrams along the optimized reaction pathways for the conversion of H_2_O_2_ to O_2_, showcasing the CAT‐mimicking performance of Ru/TiO_2‐x_ SAE, which alleviated the tumor hypoxia associated with the resistance to SDT. The amplified mechanism of Ru/TiO_2‐x_ SAE for SDT was illustrated by calculation results (Figure 5o). The significant work function difference drove electron flow from TiO_2‐x_ to single‐atomic Ru, resulting in the formation of the built‐in electric field and a barrier that facilitated e^−^/h^+^ separation under US irradiation. Both experimental and theoretical results confirmed that Ru/TiO_2‐x_ SAE held substantial potential to enhance the effectiveness of SDT.

### 2.4 In Vitro SDT of Ru/TiO_2‐x_ SAE

**Figure 6:**
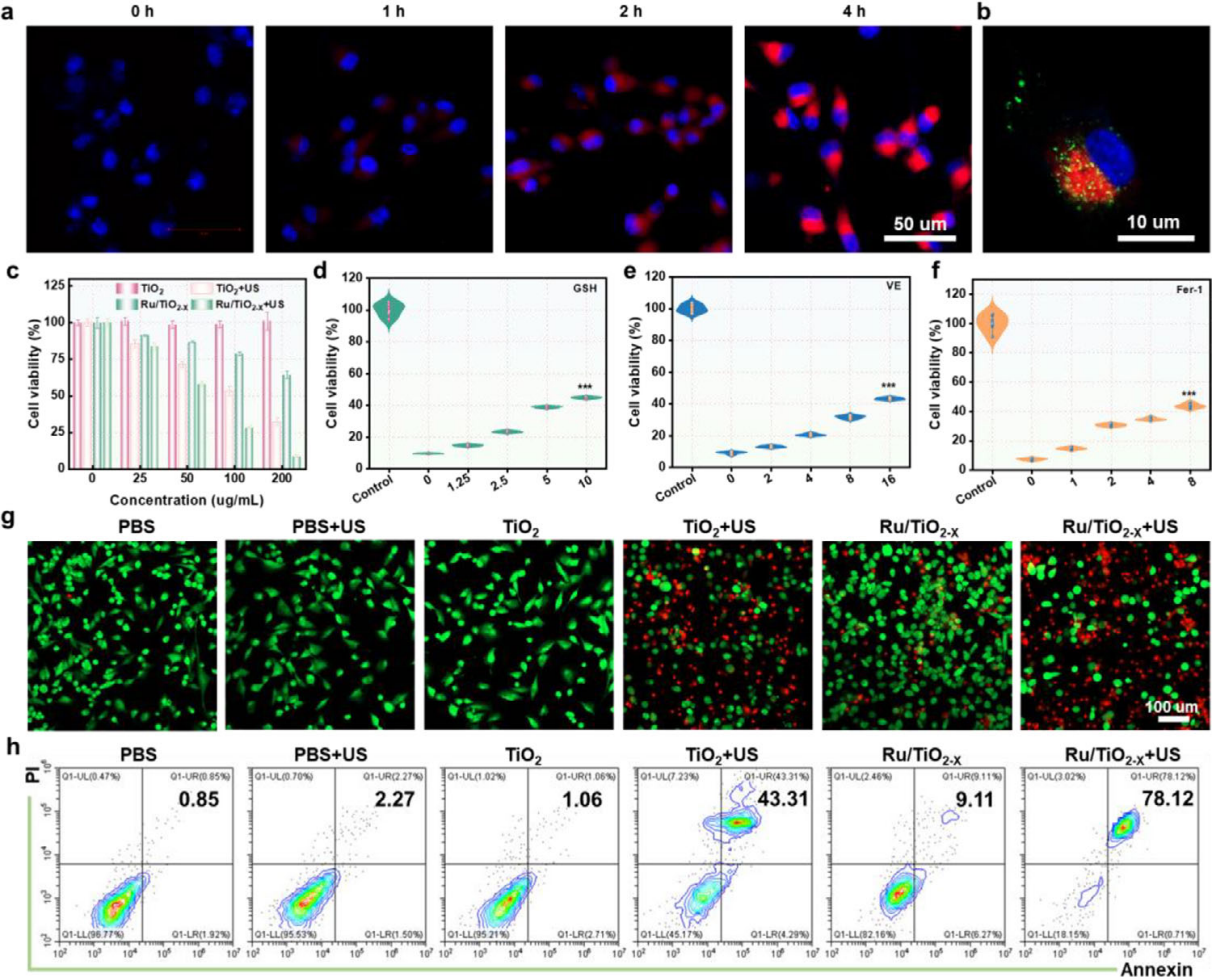
Antitumor effect of Ru/TiO_2‐x_ SAE in vitro. a) The CLSM images of GL261 cells treated with Cy5.5‐labeled Ru/TiO_2‐x_ SAE. b) The fluorescence images showed the colocalization of Ru/TiO_2‐x_ SAE with the lysosomes of GL261 cells. c) The viability of GL261 cells after a 24‐h treatment with different formulations (*n* = 5, for each group). The viability of tumor cells after a 24‐h treatment with Ru/TiO_2‐x_ SAE in the presence of d) GSH, e) VE, and f) Fer‐1. g) The CLSM images of live/dead co‐staining for GL261 cells following treatment with various formulations. h) Flow cytometry analysis of GL261 cell death rate following treatment with various formulations. ^***^ *p* < 0.001. All experiments were expressed as mean ± S.D. Statistical differences were calculated using the two‐tailed Student′s *t*‐test.

The passage discusses the evaluation of in vitro cytotoxicity through various assays, emphasizing enhanced SDT effects. The cellular uptake of Ru/TiO_2‐x_ SAE was analyzed using confocal laser scanning microscopy (CLSM), which demonstrated a time‐dependent increase in Cyanine5.5 (Cy5.5) fluorescence within GL261 cells. (**Figure** **6**a; Figure S17, Supporting Information). Additionally, ICP‐MS showed effective internalization of Ru/TiO_2‐x_ SAE by GL261 cells via endocytosis (Figure S18, Supporting Information). Confocal imaging indicated some Ru/TiO_2‐x_ SAE‐escaped lysosomes into the cytoplasm, resulting in O_2_ generation and GSH depletion (Figure 6b; Figure S19, Supporting Information). We have compared the endogenous levels of H_2_O_2_ in noncancerous LO2 cells and cancerous GL261 cells by using a H_2_O_2_‐specific probe pentafluorobenzenesulfonyl fluorescein (PBSF). As expected, GL261 cells showed stronger PBSF fluorescence over LO2 cells (Figure S20, Supporting Information), indicating that cancer cells have higher endogenous H_2_O_2_ levels compared with normal cells. The cytotoxicity of Ru/TiO_2‐x_ SAE and TiO_2_ NPs on GL261 cells was quantificationally measured using a cell counting kit 8 (CCK‐8), revealing slight growth suppression of GL261 cells due to Ru/TiO_2‐x_ SAE, linked to significant GSH depletion and oxidative stress. TiO_2_ NPs partially inhibited GL261 cell growth under US irradiation because of the SDT efficacy (Figure 6c). In contrast, Ru/TiO_2‐x_ SAE significantly inhibited GL261 cell growth under US irradiation due to pronounced GSH depletion, high e^−^/h^+^ separation efficiency, and the initiation of self‐sustaining sonodynamic ROS storms. Various antioxidants, including GSH, vitamin E (VE), and ferrostatin‐1 (Fer‐1), were used to further validate the tumor cell‐killing effects of Ru/TiO_2‐x_ SAE attributed to enhanced sonodynamic ROS storms. As anticipated, the viability of GL261 cells treated with Ru/TiO_2‐x_ SAE plus US significantly increased due to the ROS scavenging ability of the antioxidants (Figure 6d–f). The Calcein‐AM/propidium iodide (PI) co‐staining kit was conducted to visually assess the antitumor effects of different formulations. CLSM images demonstrated that Ru/TiO_2‐x_ SAE, when subjected to the US, presented a greater anti‐proliferation effect compared to TiO_2_ with the US (Figure 6g; Figure S21, Supporting Information), consistent with CCK‐8 results. Furthermore, the cell‐killing effect of Ru/TiO_2‐x_ SAE was further quantitatively analyzed through flow cytometry, confirming that Ru/TiO_2‐x_ SAE induced the highest percentage of GL261 cell death upon US irradiation. (Figure 6h; Figure S22, Supporting Information). We have studied the anti‐proliferation effect of Ru/TiO_2‐x_ SAE on different normal cells. The result demonstrated that Ru/TiO_2‐x_ SAE had a poor inhibition effect on cell growth, further confirming that Ru/TiO_2‐x_ SAE exhibited superior catalytic performances under tumor microenvironment (Figure S23, Supporting Information). Overall, these findings supported the amplified SDT effectiveness of Ru/TiO_2‐x_ SAE.

**Figure 7:**
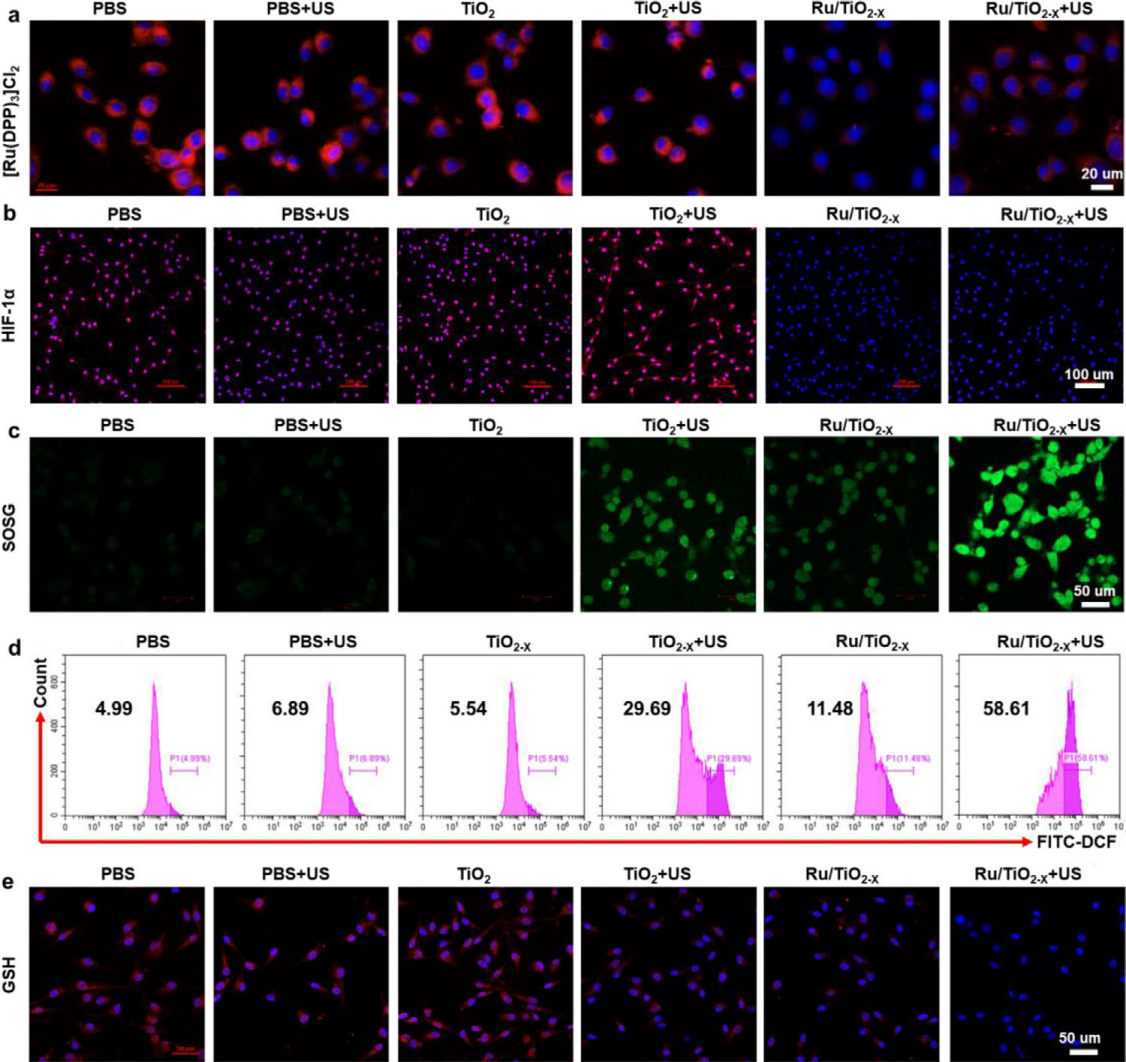
CAT‐ and GSH‐like activities of Ru/TiO_2‐x_ SAE in vitro. a) CLSM images of tumor cells stained with Ru(DPP)_3_Cl_2_ following various formulations. b) IF images of tumor cells stained with HIF‐1α following different treatments. c) SOSG fluorescence of tumor cells exposed to various formulations. d) Flow cytometry analysis of ROS intensity in GL261 cells after treatment with various formulations. e) Fluorescence images of GSH content in GL261 cells following different treatments.

The study investigated the pathway of GL261 cell death to understand the potential mechanisms behind the antitumor effects induced by Ru/TiO_2‐x_ SAE. Initially, it was found that Ru/TiO_2‐x_ SAE, which exhibited excellent CAT‐mimicking activity, can efficiently mitigate tumor hypoxia by converting overproduced H_2_O_2_ into oxygen. To assess cellular oxygen concentration, an oxygen probe, [Ru(dpp)_3_]Cl_2_ (RDPP), was used, as its red fluorescence is quenched by oxygen. CLSM images showed that the red fluorescence was obviously quenched after incubation with Ru/TiO_2‐x_ SAE, suggesting that the single‐atomic Ru in Ru/TiO_2‐x_ SAE remained effective in the generation of oxygen within cancer cells (**Figure** **7**a; Figure S24, Supporting Information). Furthermore, the expression of hypoxia‐inducible factor HIF‐1α was characterized, providing a direct measure of hypoxia levels in the tumor cells. As expected, cancer cells exhibited much brighter red fluorescence than those treated with Ru/TiO_2‐x_ SAE, indicating significant inhibition of HIF‐1α expression (Figure 7b; Figure S25, Supporting Information). This effect can be attributed to the adequate oxygen supply generated by single‐atomic Ru through H_2_O_2_ decomposition. These findings confirmed that Ru/TiO_2‐x_ SAE could potentially enhance the sonodynamic effectiveness in hypoxic tumors. Then, the remarkable GSH depletion capability and sonodynamic efficacy of Ru/TiO_2‐x_ SAE led to elevated levels of ROS. The ^1^O_2_ levels were evaluated using a specific fluorescence probe, singlet oxygen sensor green (SOSG). CLSM images revealed considerable green fluorescence in the presence of Ru/TiO_2‐x_ SAE, indicating effective GSH depletion (Figure 7c; Figure S26, Supporting Information). When subjected to ultrasound (US), Ru/TiO_2‐x_ SAE exhibited stronger green fluorescence intensity compared to TiO_2_ NPs combined with US irradiation, which was ascribed to the significant GSH depletion, oxygen generation, and high e^−^/h^+^ separation efficiency. The excessive ROS levels were further assessed using flow cytometry with a ROS probe, 2′,7′‐dichlorofluorescin diacetate (DCFH‐DA). Consistent with CLSM results, Ru/TiO_2‐x_ SAE combined with the US showed the highest fluorescence intensity compared to other formulations (Figure 7d; Figure S27, Supporting Information). In addition, the cellular GSH levels were evaluated, with immunofluorescence and total glutathione assay results demonstrating that Ru/TiO_2‐x_ SAE effectively depleted GSH owing to the single‐atomic Ru, an effect that intensified upon US irradiation. (Figure 7e; Figures S28 and S29, Supporting Information). These findings confirmed that Ru/TiO_2‐x_ SAE can alleviate tumor hypoxia and deplete overproduced GSH, thereby amplifying SDT effectiveness.

**Figure 8:**
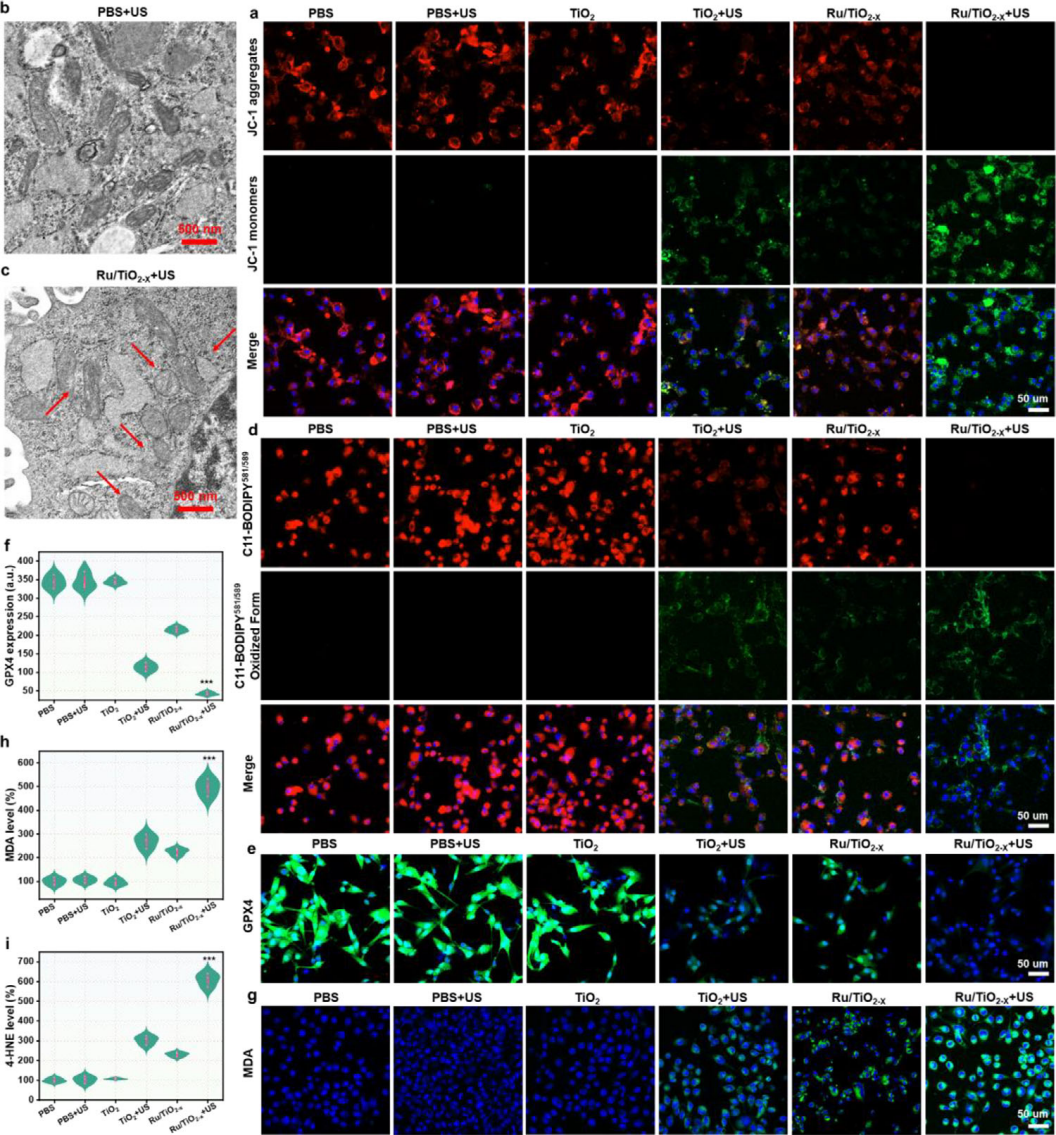
Sonodynamic‐amplified ferroptosis induced by Ru/TiO_2‐x_ SAE in vitro. a) CLSM images of GL261 cells measured with a JC‐1 kit following various treatments. Bio‐TEM images of GL261 cells after treatment with b) PBS plus US or c) Ru/TiO_2‐x_ SAE plus US. d) Confocal images of tumor cells stained with C11‐BODIPY^581/589^ following a 24‐h treatment with various formulations. e) CLSM images and the corresponding quantification of f) GPX4 expression in cancer cells after various treatments. g) CLSM images of cancer cells stained with MDA kit following different treatments. Quantitative analysis of enzyme‐linked immunosorbent assay (ELISA) for h) MDA and i) 4‐HNE levels after different treatments. ^***^ *p* < 0.001. All experiments were expressed as mean ± S.D. Statistical differences were calculated using the two‐tailed Student′s *t*‐test.

To gain insights into how Ru/TiO_2‐x_ SAE can induce ferroptosis in cancer cells, a fluorescence probe 5,5′,6,6′‐tetrachloro‐1,1′,3,3′‐tetraethyl‐imidacarbocyanine iodide (JC‐1) was used to measure the polarization in mitochondrial membrane potential (MMP) by monitoring its fluorescence shift from red to green. As seen in CLSM images (**Figure** **8**a; Figure S30, Supporting Information), the red fluorescence signal decreased in the TiO_2_ NPs group with US irradiation, while the green fluorescence signals were enhanced, suggesting damage to the MMP. In contrast, Ru/TiO_2‐x_ SAE combined with US irradiation resulted in enhanced green fluorescence and diminished red fluorescence, implying extreme MMP polarization. Furthermore, the Bio‐TEM was employed for a deeper evaluation of mitochondrial damage. As depicted in Figure 8b,c, GL261 cells treated with Ru/TiO_2‐x_ SAE and subjected to US irradiation showed notable mitochondrial shrinkage, with cristae decreasing or even disappearing‐a hallmark of ferroptosis. The intrinsic characteristics of membrane LPO were further examined to indicate the ferroptotic mechanism triggered by Ru/TiO_2‐x_ SAE using a fluorescence probe C11‐BODIPY^581/589^, based on the fluorescence change from red to green. Confocal images demonstrated that Ru/TiO_2‐x_ SAE combined with the US significantly amplified green fluorescence signals and diminished red fluorescence signals (Figure 8d; Figure S31, Supporting Information), indicating an accumulation of lipid peroxides triggered by Ru/TiO_2‐x_ SAE, which greatly promoted lethal ferroptosis. However, the LPO in tumor cells is quickly mitigated by the endogenous glutathione peroxidase 4 (GPX4) system. As previously reported, depleting GSH and producing excessive ROS could effectively downregulate GPX4 expression, leading to ferroptosis. CLSM images showed that Ru/TiO_2‐x_ SAE partially suppressed GPX4 expression (Figure 8e,f; Figure S32, Supporting Information), further enhancing the inhibitory efficacy upon US irradiation. In addition, ferroptosis indicators, including malondialdehyde (MDA) and 4‐hydroxynonenal (4‐HNE), were selected to prove Ru/TiO_2‐x_ SAE‐triggered ferroptosis. It can be seen in Figure 8g–i, and Figures S33 and S34 (Supporting Information) that a significant amplification in MDA and 4‐HNE levels in GL261 cells incubated with Ru/TiO_2‐x_ SAE combined with US irradiation. Overall, these results provided evidence that Ru/TiO_2‐x_ SAE effectively initiated the LPO bioprocess and inhibited GPX4 expression, resulting in an irreversible tumor ferroptosis.

### 2.5 In Vivo Tumor Therapy

**Figure 9:**
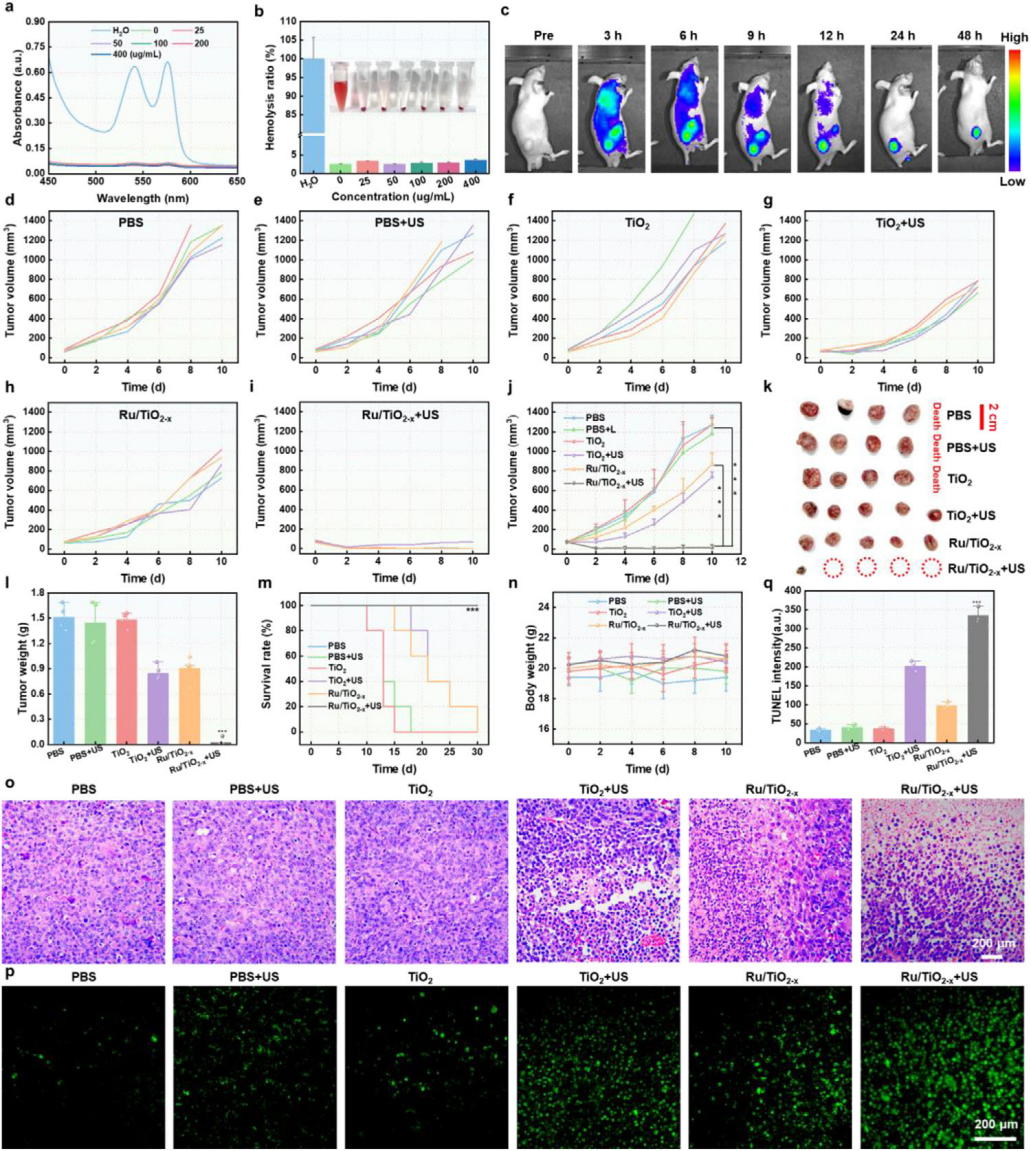
Antitumor effect of Ru/TiO_2‐x_ SAE in vivo. a) UV–vis spectra of erythrocytes exposed to a range of Ru/TiO_2‐x_ SAE. b) The corresponding percentages of hemolysis induced by various concentrations (Inset: photograph of centrifuge tubes with supernatants from erythrocytes treated with varying concentrations of Ru/TiO_2‐x_ SAE). c) Fluorescence images of GL261 mice at different time points postinjection with Cy5.5‐labeled Ru/TiO_2‐x_ SAE. Tumor growth curves d–j) of GL261 tumor‐bearing mice following intravenous injection of different formulations. k) Representative photograph of excised tumors after various treatments. l) Average tumor weights of mice on day 10 post different treatments. m) Kaplan–Meier survival curves of mice following various formulations. n) Body weight curves of GL261 tumor‐bearing mice following different formulations. o) H&E staining of tumor slices from various groups following different formulations. p) TUNEL staining of tumor slices and the q) corresponding quantification from various groups following different formulations. ^***^ *p* < 0.001. All experiments were expressed as mean ± S.D. Statistical differences were calculated using the two‐tailed Student′s *t*‐test.

The biocompatibility of Ru/TiO_2‐x_ SAE was evaluated using various assays. First, a hemolysis test was employed to determine the degree of erythrolysis in blood contact with Ru/TiO_2‐x_ SAE. UV–vis results indicated minimal hemolysis of red blood cells (less than 5%) even at a high concentration of 400 µg mL^−1^ Ru/TiO_2‐x_ SAE (**Figure** **9**a,b), which confirmed that Ru/TiO_2‐x_ SAE possessed good biocompatibility. Subsequently, biochemical parameters were selected to evaluate the biosafety of Ru/TiO_2‐x_ SAE. No significant alterations in these indices were detected following the intravenous administration of Ru/TiO_2‐x_ SAE (Figure S35, Supporting Information). Furthermore, haematoxylin and eosin (H&E) staining indicated no significant damage in major organs (Figure S36, Supporting Information), offering substantial evidence of the excellent biocompatibility profile of Ru/TiO_2‐x_ SAE. Moving forward, we established a GL261 glioblastoma model in mice. Fluorescence imaging revealed that Cy5.5‐labeled Ru/TiO_2‐x_ SAE revealed a satisfied accumulation in the tumor region (Figure 9c; Figure S37, Supporting Information), ascribed to the enhanced permeability and retention effect. The biodistribution of Ru/TiO_2‐x_ SAE in tumor‐bearing mice, as shown in Figure S38 (Supporting Information), further confirmed the satisfactory tumor accumulation efficacy, which significantly implied the potential therapeutic efficacy of sonodynamic‐amplified ferroptosis.

The enhanced SDT efficacy of ferroptosis induced by Ru/TiO_2‐x_ SAE was assessed in GL261 tumor‐bearing mice. These mice were randomly divided into six groups: PBS, PBS plus US, TiO_2_ NPs, TiO_2_ NPs plus US, Ru/TiO_2‐x_ SAE, and Ru/TiO_2‐x_ SAE plus US. As depicted in Figure 9d–j, TiO_2_ NPs showed a partial inhibition of tumor growth upon US irradiation, likely due to a significant sonodynamic effect. Ru/TiO_2‐x_ SAE demonstrated a moderate suppression of tumor growth, attributed to the synergistic effect of ferroptosis involving single‐atomic Ru‐mediated GSH depletion. Furthermore, the antitumor efficacy was markedly enhanced under US irradiation, which can be attributed to the adequate oxygen supply generated by single‐atomic Ru, sufficient oxygen vacancies, and high e^−^/h^+^ separation efficiency (Figure 8k–l). This significantly enhanced the sonodynamic‐amplified ROS generation efficacy and improved the survival rate of tumor‐bearing mice (Figure 9m). In addition, no significant variety in body weight was noticed in the Ru/TiO_2‐x_ SAE plus US group (Figure 9n), indicating outstanding biosafety. The therapeutic effect was proved through H&E, terminal deoxynucleotidyl transferase‐mediated dUTP‐biotin nick end labeling (TUNEL), and immunofluorescence staining of excised tumors. It can be seen in H&E images (Figure 9o) that Ru/TiO_2‐x_ SAE caused the most damage to tumors when exposed to US irradiation compared to other formulations. TUNEL staining results demonstrated that mice treated with Ru/TiO_2‐x_ SAE plus the US experienced the most severe cell death (Figure 9p,q; Figure S39, Supporting Information), indicating the profound therapeutic outcomes, consistent with H&E results. Immunofluorescence images showed that Ru/TiO_2‐x_ SAE triggered the lowest expression of HIF‐1α (Figure S40, Supporting Information), suggesting the alleviation of hypoxic TME. Furthermore, the significant decrease of GPX4 expression induced by Ru/TiO_2‐x_ SAE also provided substantial evidence for the sonodynamic‐strengthened ferroptosis (Figure S41, Supporting Information). These results strongly confirmed the sonodynamic‐boosted ferroptosis induced by Ru/TiO_2‐x_ SAE and underscored its potential for cancer therapy.

## 3 Conclusion

In this study, we have developed a Ru single‐atom nanozyme featuring abundant oxygen vacancies and high e^−^/h^+^ separation efficiency, which enhanced its response to TME and induced ferroptosis in tumors. The fabrication process involved reducing hydrogen to generate oxygen vacancies in TiO_2‐x_ NPs, which facilitated the immobilization of single‐atomic Ru. Ru‐anchored oxygen vacancies not only altered the electronic structure of the nanosensitizer, improving e^−^/h^+^ separation efficiency, but also increased oxygen adsorption and accelerated reaction kinetics, thereby enhancing the selectivity and reactivity of the sonosensitizer. Furthermore, Ru/TiO_2‐x_ SAE, with its atomically dispersed active metal sites, demonstrated exceptional catalytic activity, generating O_2_ from H_2_O_2_ to alleviate hypoxia in the TME. It also downregulated GPX4 expression by depleting the overproduced reductive GSH, thus triggering SDT‐amplified ferroptosis. DFT calculations, combined with experimental results, revealed the superior catalytic mechanism and therapeutic effect of Ru/TiO_2‐x_ SAE, which was enriched with oxygen vacancies, compared to traditional TiO_2_‐based sonosensitizers. Collectively, these results highlighted the innovative role of single‐atom Ru in optimizing sonosensitizers, offering new insights into the development of single‐atom sonosensitizers and advancing the application of SDT‐amplified ferroptosis.

## 4 Experimental Section

### Animal Ethics Statement

The protocol for conducting animal experiments was approved by the Ethical Committee of Fujian Medical University (Approval Number: IACUC FJMU2022‐0608).

### Statistical Analysis

All experiments were expressed as mean ± S.D. Statistical analyses were performed using Origin 2024 software. For experiments requiring statistical analysis, at least three separate experiments were conducted (*n* ≥ 3). Statistical significance was assessed using a two‐tailed Student's *t*‐test, with a threshold of *p* < 0.05 considered statistically significant. The levels of significance are denoted as follows: *p* < 0.05 (^*^), *p* < 0.01 (^**^), *p* < 0.001 (^***^). Unless otherwise specified, all comparisons were made relative to the control group.

## Conflict of Interest

The authors declare no conflict of interest.

## Author Contributions

Y.Z., D.W., and C.D. contributed equally to this work. Y.Z. performed conceptualization, data curation, resources, and visualization, wrote the original draft, and edited the final manuscript. D.W. performed data curation and investigation. C.D. performed data curation, visualization, and methodology. T.W. performed software, resources, and supervision. P.W. performed data curation and software. H.Z. performed visualization and software. G.L. performed a formal analysis. S.Z. performed a formal analysis. L.S. performed software. L.Y. performed software and investigation. Y.H. performed data curation and methodology. H.W. performed resources and methodology. L.L. performed the supervision and edited the final draft. C.D. performed funding acquisition and supervision. X.C. performed visualization, edited the final manuscript, and provided supervision.

## Supporting information



Supporting Information

## Data Availability

The data that support the findings of this study are available from the corresponding author upon reasonable request.
