# Supplementary material for: Ruthenium Single‐Atom Nanozyme Driven Sonosensitizer with Oxygen Vacancies Enhances Electron–Hole Separation Efficacy and Remodels Tumor Microenvironment for Sonodynamic‐Amplified Ferroptosis
Source: Adv Sci (Weinh). 2025 Apr 25;12(22):2416997. doi: 10.1002/advs.202416997 (PMC12165091; doi:10.1002/advs.202416997)
Supplement: Supplementary file 1 — Supporting Information [file ADVS-12-2416997-s001.docx]

**Supporting Information**

**Ruthenium single-atom nanozyme driven sonosensitizer with oxygen vacancies enhances electron-hole separation efficacy and remodels tumor microenvironment for sonodynamic-amplified ferroptosis**

Yang Zhu^+^, Dengliang Wang^+^, Chengzhong Du^+^, Tiantian Wu*, Penghui Wei, Hongjia Zheng, Guanting Li, ShunZhe Zheng, Lichao Su, Lingjun Yan, Yongrui Hu, Huimin Wang, Lisen Lin*, Chenyu Ding*, Xiaoyuan Chen*

Y. Zhu, D. Wang, C. Du, P. Wei, H. Zheng, G. Li. S. Zheng, L. Su, L. Yan, Y. H, H. Wang, L. Lin, C. Ding

Email: lisen.lin@fzu.edu.cn; dingcydr@163.com

Department of Neurosurgery, Neurosurgery Research Institute, The First Affiliated Hospital of Fujian Medical University, Fuzhou, Fujian 350209, China

Fujian Provincial Institutes of Brain Disorders and Brain Sciences, The First Affiliated Hospital Fujian Medical University, Fuzhou, Fujian 350209, China

T. W

Email: WuTiantian@hainmc.edu.cn

School of Pharmaceutical Sciences/NHC key laboratory of tropical disease control/School of Tropical Medicine, Hainan Medical University, Haikou 571199, P. R. China

X. Chen

Departments of Diagnostic Radiology, Surgery, Chemical and Biomolecular Engineering, and Biomedical Engineering, Yong Loo Lin School of Medicine and College of Design and Engineering, National University of Singapore, Singapore, 119074, Singapore

Clinical Imaging Research Centre, Centre for Translational Medicine, Yong Loo Lin School of Medicine, National University of Singapore, Singapore 117599, Singapore

Nanomedicine Translational Research Program, Yong Loo Lin School of Medicine, National University of Singapore, Singapore 117597, Singapore

Email: chen.shawn@nus.edu.sg

^+^These authors contributed equally.

**Chemicals**

Hydrogen peroxide (H_2_O_2_), acetic acid (HAc), sodium acetate (NaAc), and ethanol were purchased from Sinopharm Chemical Reagents (Shanghai, China). 3,3',5,5'-tetramethylbenzidine (TMB), and C11-BODIPY^581/591^ were provided by Sigma-Aldrich (St. Louis, USA). Hoechst 33342, 2′,7′-dichlorofluorescin diacetate (DCFH-DA), cell count kit-8 (CCK-8), thiobarbituric acid (TBA), AM/PI, and 1,1',3,3'-tetraethyl-5,5',6,6'-tetrachloroimidacarbocyanine iodide (JC-1) were bought from Beyotime (Shanghai, China). Dulbecco's modified eagle medium (DMEM) was purchased from Hyclone (Logan, USA). Cyanine 5.5 monosuccinimidyl ester (Cy5.5-NHS), ELISA, and annexin V-FITC/PI apoptosis detection kit were purchased from Beijing Solarbio Science & Technology Co., Ltd. (Beijing, China). Live & Dead Bacterial Staining Kit (Cat#40274ES60) and GMyc-PCR Mycoplasma Test Kit (Cat#40601) was purchased from Yeasen Biotechnology (Shanghai) Co., Ltd.. 20 mm glass-bottom dishes, and centrifuge tubes were obtained from NEST Biotechnology Co. Ltd. (Wuxi, China). Deionized (DI) water was obtained from a Milli-Q water purification system.

**Methods**

**Ethical regulations**

BALB/c nude mice aged about 6-8 weeks was brought from Beijing Vital River Laboratory Animal Technology Co. The protocol for conducting animal experiments was approved by the Ethical Committee of Fujian Medical University (Approval Number: IACUC FJMU2022-0608). All mice were maintained at 12 h light-dark cycle within 22-26 °C and 40-65% humidity. The maximal tumor burden permitted was 1500 mm^3^

**Preparation of Ru/TiO_2-x_ SAE**

To the mixed solution of 0.92 mL ammonia water, 46 mL ethanol, and 8.6 mL deionized water, 1.72 mL of tetraethyl orthosilicate (TEOS) was introduced, and the mixture was stirred for 4 h. The resulting SiO_2_ NPs were obtained via centrifugation at 10,000 rpm for 10 min. Subsequently, 0.4g of hydroxypropyl cellulose, 190 mL of deionized water, and 0.96 mL ethanol were added to the SiO_2_ NPs and stirred for 10 min. Meanwhile, 8 mL of a tetrabutyl titanate solution in 36 mL ethanol was slowly added to the mixture. This mixture was then heated to 85 °C and maintained for 120 min. After refluxing, the product was washed with ethanol and resuspended in 20 mL of deionized water to yield the SiO_2_@TiO_2_ NPs. To this, 4 mL of a 2.5 M NaOH solution was added, and the mixture was stirred for 6 h. The product was washed with deionized water and ethanol, then dried at 60 °C. The product was redispersed in deionized water at a concentration of 10 mg/mL and mixed with a 1 mmol HCl solution for 30 minutes. The TiO_2_ NPs were obtained by centrifugation at 10,000 rpm, washed with ethanol, and dried at 60 °C. Finally, 500 mg of TiO_2_ NPs and 10 mg of RuCl_3_ were redispersed in 50 mL of deionized water and stirred for 12 h. The TiO_2_@Ru NPs were obtained by centrifugation at 11,000 rpm, washed with ethanol, and dried under vacuum overnight. The TiO_2_@Ru NPs were transferred to a tube furnace and heated to 300 °C in an atmosphere composed of 5% hydrogen and 95% argon at a heating rate of 5 °C/min.

**CAT-like activity of Ru/TiO_2-x_ SAE**

The CAT-like activity of Ru/TiO_2-x_ SAE were evaluated assessed by measuring oxygen generation using a dissolved oxygen analyzer. 20 mM H_2_O_2_ was dissolved in different buffer solution with pH values of 5.0 or 7.4, and 100 ug/mL Ru/TiO_2-x_ SAE was added to the solution. Oxygen concentration was immediately detected, and the data were recorded continuously for 5 min.

**In vitro SDT of Ru/TiO_2-x_ SAE**

The production of ^1^O_2_ was quantified using SOSG fluorescence probe. A mixture of Ru/TiO_2-x_ SAE or TiO_2_ NPs (100 μg/mL), SOSG solution, and 1 mM H_2_O_2_ was subjected to US irradiation (1 W/cm^2^). Fluorescence was then assessed using a fluorescence spectrophotometer. Additionally, to further investigate ^1^O_2_ production, TEMP, a trapping agent, was employed. The same concentration of TiO_2_ NPs or Ru/TiO_2-x_ SAE in aqueous dispersion was exposed to US irradiation (1 W/cm^2^). The ^1^O_2_ production was detected using an EPR spectrometer

**GSHOx-like activity of Ru/TiO_2-x_ SAE**

A 5 mM GSH solution was added to Ru/TiO_2-x_ SAE or TiO_2_ NPs (100 μg/mL) solution and incubated for 2 h. Then, a 2 mg/mL DTNB solution was added to the mixed solution. After centrifugation, the resulting solution was analyzed using a UV-Vis spectrophotometer.

**Cellular uptake**

GL261 cells were seeded into confocal dishes and cultured overnight. The medium was then replaced with 100 μg/mL Cy5.5-labeled Ru/TiO_2-x_ SAE, and the cells were incubated for varying time points (0, 1, 2, and 4 h). After incubation, the cells were treated with DMEM containing 20 ug/mL Hoechst and 10 uM LysoTracker for 20 min. Fluorescence imaging was then performed using a CLSM.

**In vitro SDT**

GL261 cells were seeded in confocal dishes and cultured for 12 h. The fresh DMEM containing 100 ug/mL Ru/TiO_2-x_ SAE or TiO_2_ NPs was added. After 4 h of incubation, the cells were exposed to or without US irradiation (1 W/cm^2^) for 5 min. The cells were further cultured for 20 h. Cell viability was assessed using the CCK8 assay and measured using a BioTek microplate reader.

**live and dead assay**

GL261 cells were seeded in confocal dishes and cultured overnight. The fresh DMEM containing 100 ug/mL Ru/TiO_2-x_ SAE or TiO_2_ NPs was added. After 4 h of incubation, the cells were exposed to or without US irradiation (1 W/cm^2^) for 5 min. The cells were then cultured for an additional 8 h. Tumor cells were stained using Calcein-AM and PI according to the manufacturer’s protocol and imaged using a CLSM.

**Flow cytometry analysis**

GL261 cells were seeded in 6-well plates and incubated overnight. The fresh DMEM containing 100 ug/mL Ru/TiO_2-x_ SAE or TiO_2_ NPs was added. After 4 h of incubation, the cells were exposed to or without US irradiation (1 W/cm^2^) for 5 min. The cells were then cultured for an additional 8 h. Tumor cells were stained using Annexin-v and PI according to the manufacturer’s protocol and measured using a Beckman flow cytometry.

**Detection of intracellular O_2_ level**

GL261 cells were seeded in confocal dishes and incubated for 12 h. T The fresh DMEM containing 100 ug/mL Ru/TiO_2-x_ SAE or TiO_2_ NPs was added. After 8 h of incubation, the cells were exposed to or without US irradiation (1 W/cm^2^) for 5 min. Tumor cells were stained using a O_2_ probe Ru(DPP)_3_]Cl_2_ and Hoechst 33342 and imaged using a CLSM.

**HIF-1α and GPX4 expression**

GL261 cells were seeded in confocal dishes and cultured overnight. The fresh DMEM containing 100 ug/mL Ru/TiO_2-x_ SAE or TiO_2_ NPs was added. After 4 h of incubation, the cells were exposed to or without US irradiation (1 W/cm^2^) for 5 min. After treatments, tumor cells were fixed with PBS containing 1% paraformaldehyde (PFA) for 15 min and permeabilized with PBS containing 0.2% Triton X-100 for 10 min. Following permeabilization, the cells were blocked with PBS containing 10% goat serum for 50 minutes at room temperature. The cells were then incubated overnight at 4°C with primary antibodies against HIF-1α or GPX4 in a humidified chamber. Afterward, they were treated with secondary antibodies (Alexa Fluor® 568 Conjugate or Alexa Fluor® 488 Conjugate) at room temperature for 1 h. The expression levels of HIF-1α and GPX4 were assessed by detecting fluorescence using confocal microscopy.

**In vitro ROS level**

GL261 cells were seeded in confocal dishes and cultured for 12 h. the fresh DMEM containing 100 ug/mL Ru/TiO_2-x_ SAE or TiO_2_ NPs was added. After 4 h of incubation, the cells were exposed to or without US irradiation (1 W/cm^2^) for 5 min. Tumor cells were stained using a ^1^O_2_ probe SOSG and Hoechst 33342, then imaged using a CLSM.

**Measurement of mitochondrial membrane potential**

GL261 cells were seeded in in confocal dishes and incubated overnight. Subsequently, the fresh DMEM containing 100 ug/mL Ru/TiO_2-x_ SAE or TiO_2_ NPs was added. After 4 h of incubation, the cells were exposed to or without US irradiation (1 W/cm^2^) for 5 min. Tumor cells were stained using a JC-1 probe and Hoechst 33342, then imaged by CLSM.

**Lipid peroxidation**

GL261 cells were seeded in in confocal dishes and incubated overnight. The fresh DMEM containing 100 ug/mL Ru/TiO_2-x_ SAE or TiO_2_ NPs was added. After 4 h of incubation, tumor cells were exposed to or without US irradiation (1 W/cm^2^) for 5 min and further incubated for 8 h. After incubation, the cells were washed for three times with DMEM and stained with BODIPY^581/591^-C11 probe (10 μM) for 20 min. Fluorescence imags were then capturedand analyzed using CLSM.

**MDA level**

GL261 cells were seeded in 6-well plates and incubated for 12 h. The cells were then treated with100 ug/mL Ru/TiO_2-x_ SAE or TiO_2_ NPs for 4 h. Then, tumor cells were exposed to or without US irradiation (1 W/cm^2^) for 5 min and further incubated for 20 h. Following incubation, tumor cells were treated according to the manufacturer’s protocol of MDA kit and measured using a BioTek microplate reader.

**In vivo fluorescence imaging**

Cy5.5-labeled Ru/TiO_2-x_ SAE (15 mg/kg) was intravenously injected to GL261 tumor-bearing mice. At various time points after injection, NIR fluorescence images were captured using an IVIS in vivo Imaging System. The imaging was performed three times, and the data were analyzed using Living Image software.

**In vivo SDT**

Six groups of GL261 tumor-bearing mice were randomly assigned as follows: PBS, PBS + US, TiO_2_ NPs, TiO_2_ NPs + US, Ru/TiO_2-x_ SAE, and Ru/TiO_2-x_ SAE + US. After intravenous injection of 12 h, the mice in the US, TiO_2_ NPs + US, Ru/TiO_2-x_ SAE + US groups were subjected to US irradiation (1.0 W/cm^2^) for 10 min. Tumor volumes and body weights were recorded every two days. Two days after treatment, tumors from all groups were collected for histological analysis, including IF, H&E, and TUNEL staining. On day 10 post-treatment, the mice were euthanized by painless cervical dislocation. Major organs and blood samples were collected from each group which was evaluated via histological analysis and blood biochemistry.

**Reporting summary**

Further information on research design is available in the Nature Portfolio Reporting Summary linked to this article.

**Data availability**

All data are available in the main text, Supplementary Information, or Source Data file. If any raw data files are needed in another format, they are available from the corresponding author upon request. Source data are provided with this paper.

**Supplementary Figures

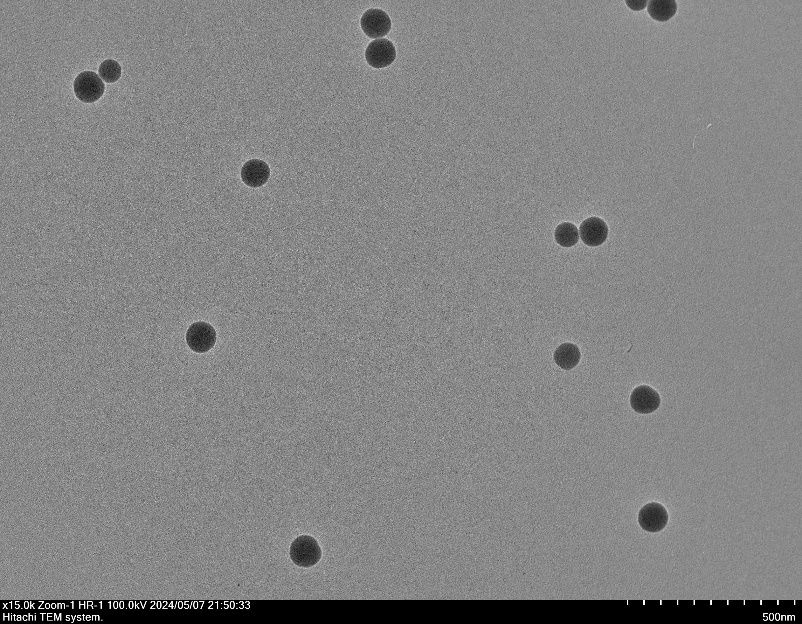
**

**Figure S1. TEM image of SiO_2_ NPs.**

**
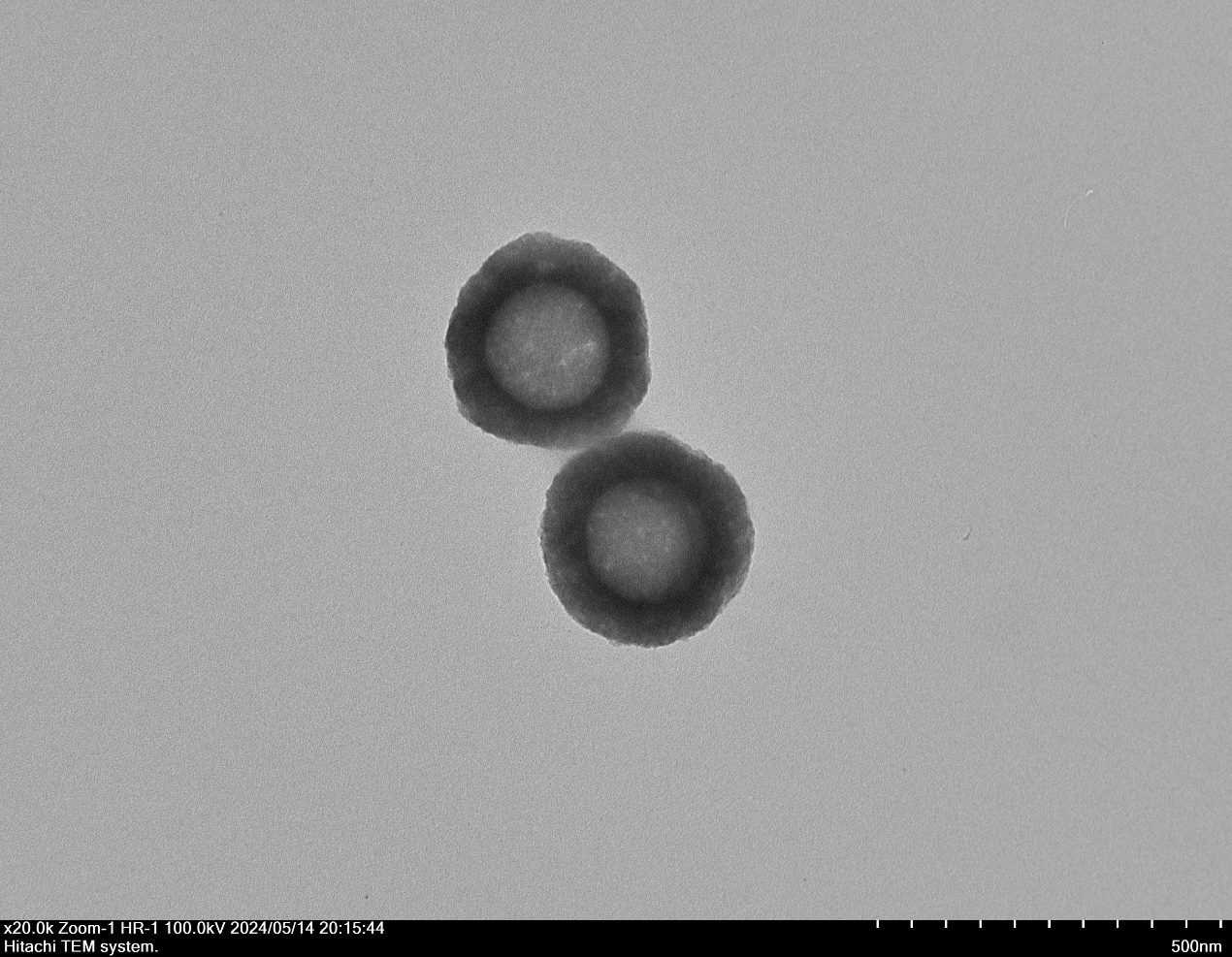
**

**Figure S2. TEM image of TiO_2_ NPs.**

**
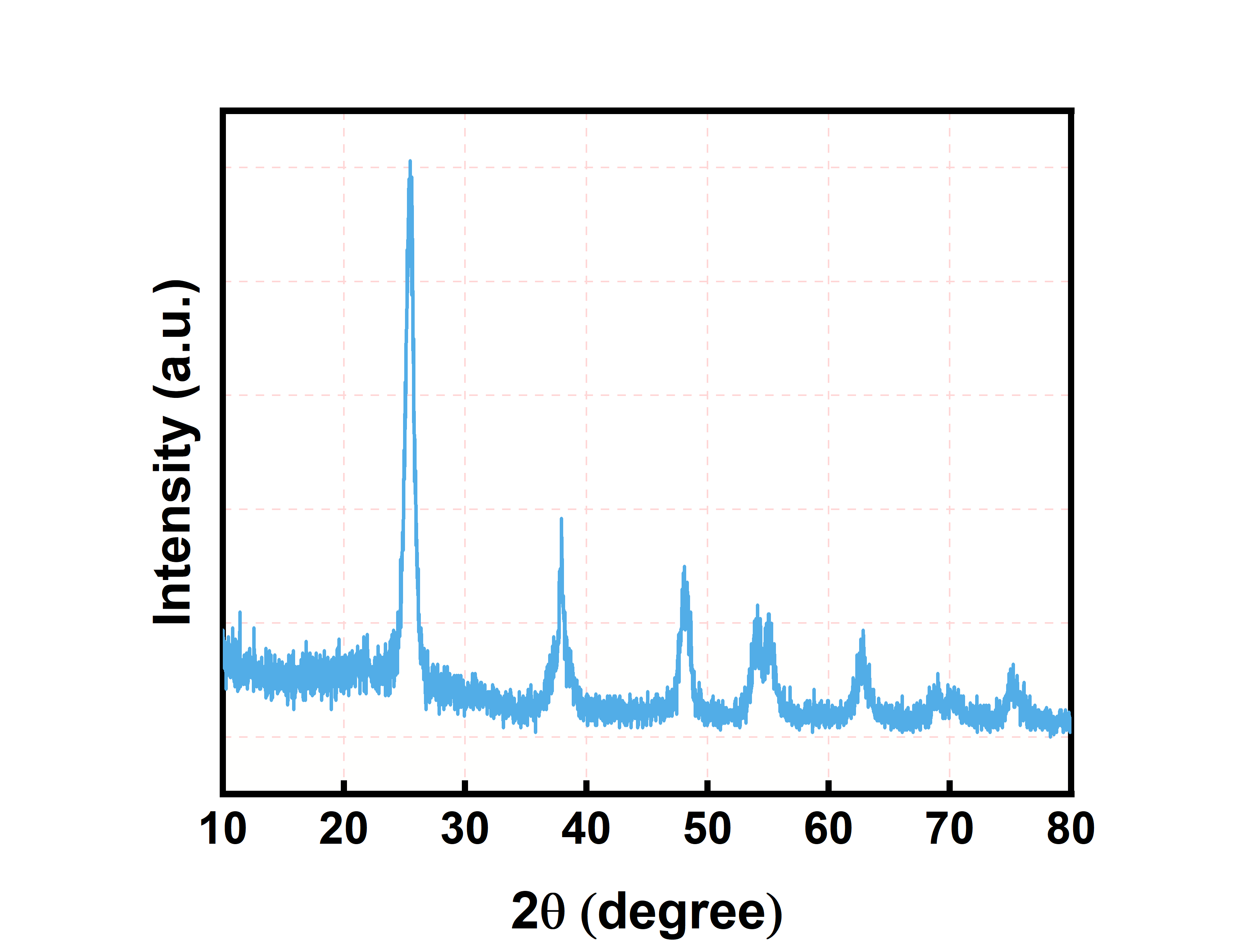
**

**Figure S3. XRD pattern of Ru/TiO_2-x_ SAE.**

**
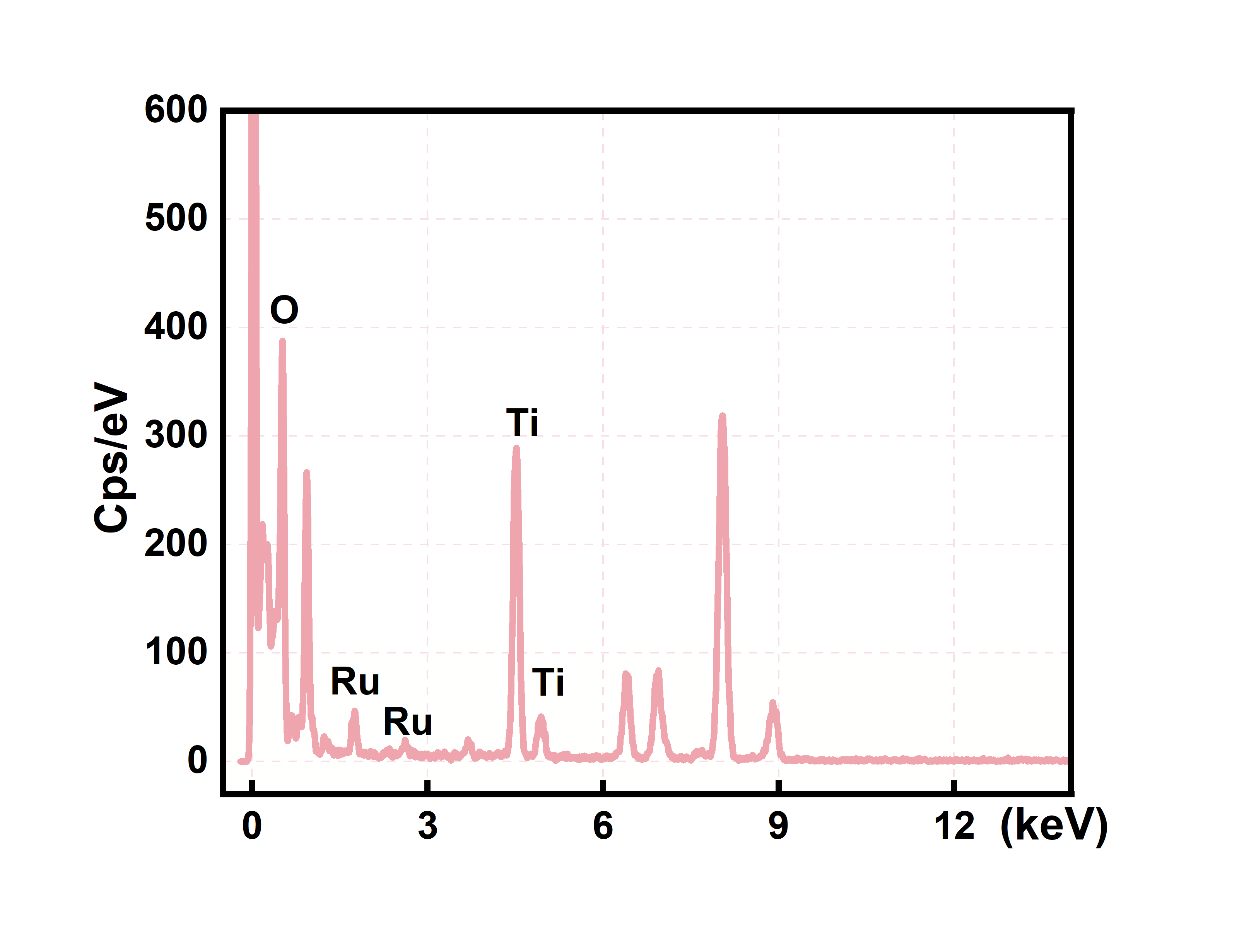
**

**Figure S4. EDX spectrum of Ru/TiO_2-x_ SAE.**

**
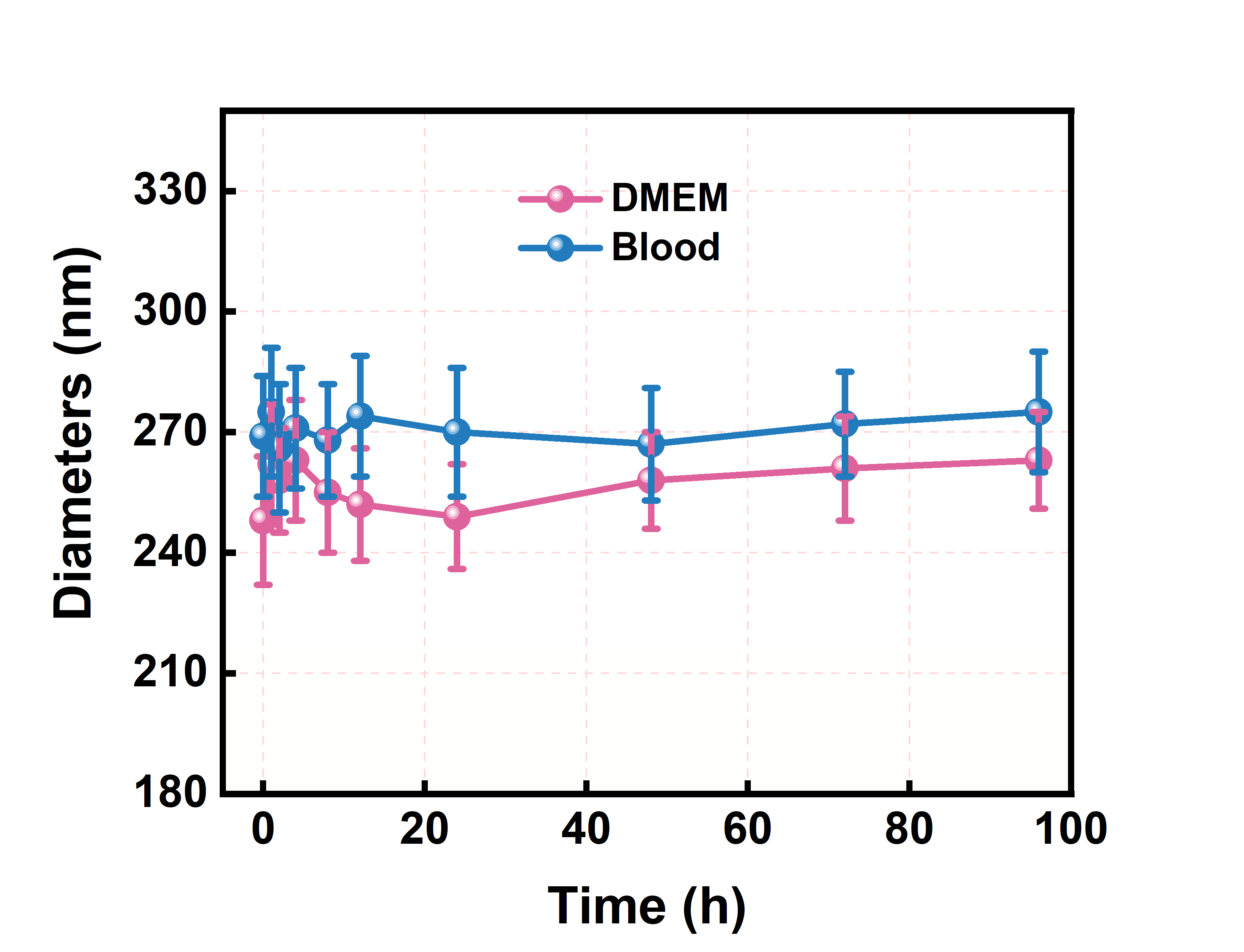
**

**Figure S5. The stability of Ru/TiO_2-x_ SAE in DMEM and blood sample.**


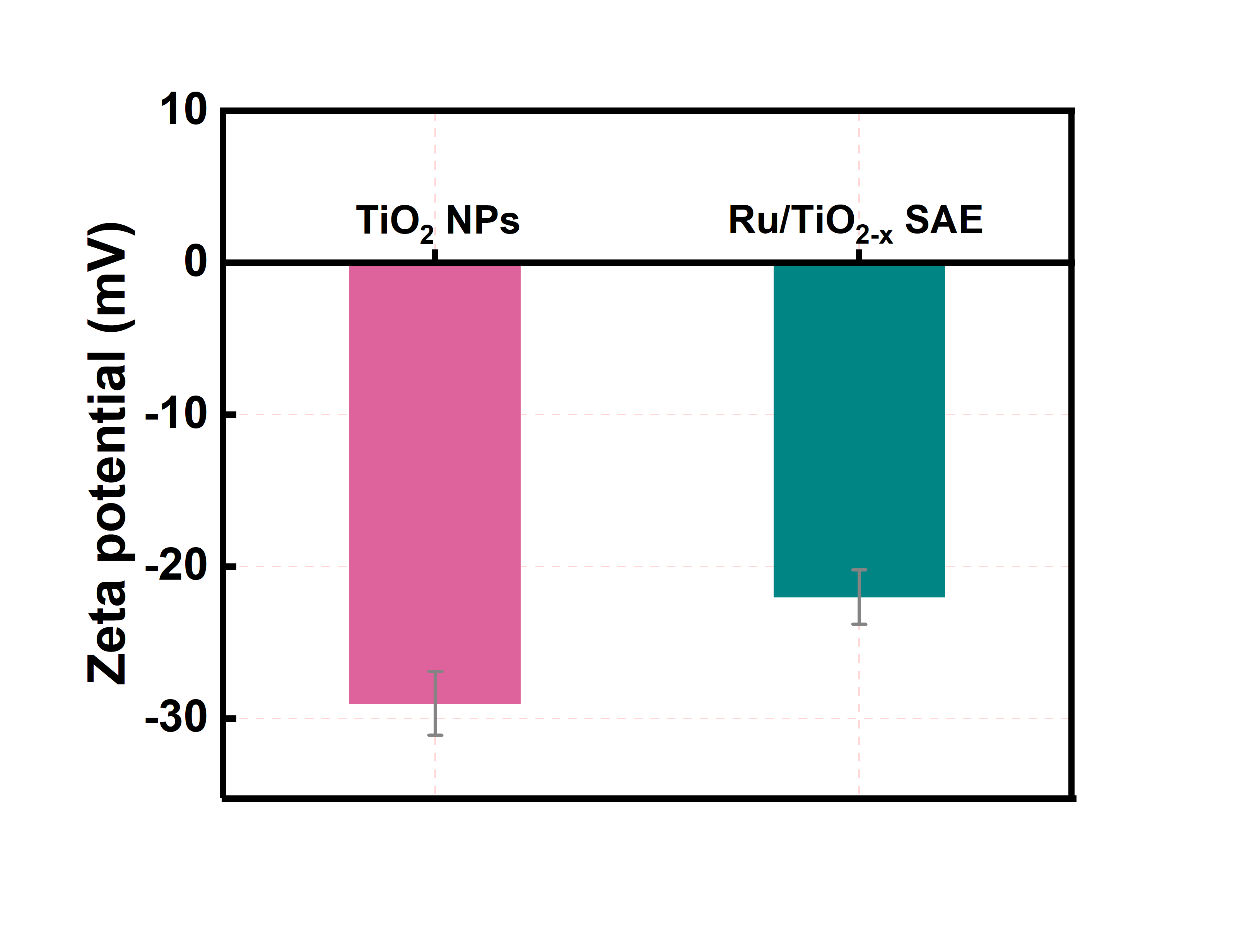


**Figure S6. The zeta potentials of both Ru/TiO_2-x_ SAE and TiO_2_ NPs.**

**
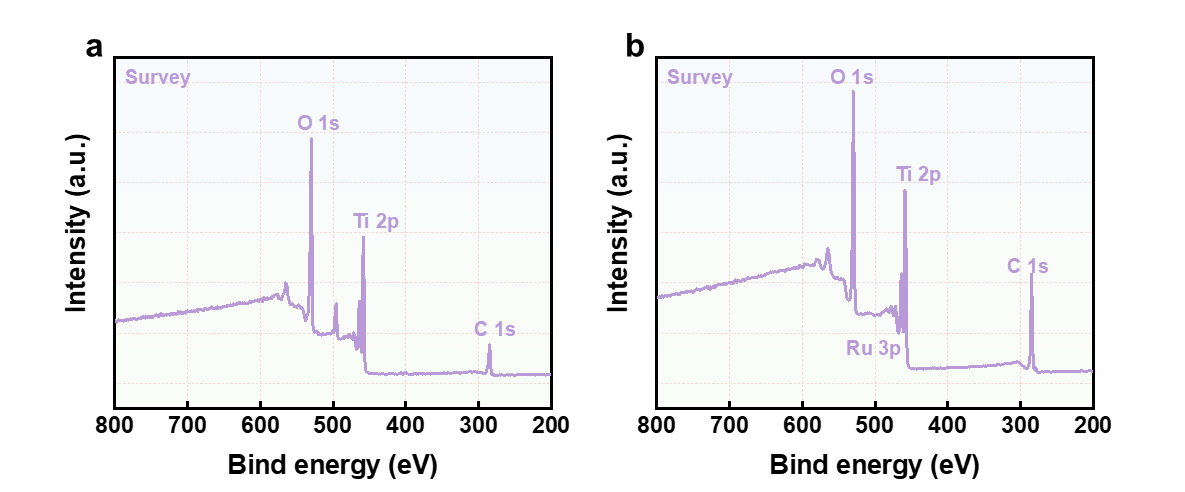
**

**Figure S7. The survey XPS of (a) TiO_2_ NPS and (b) Ru/TiO_2-x_ SAE.**

**
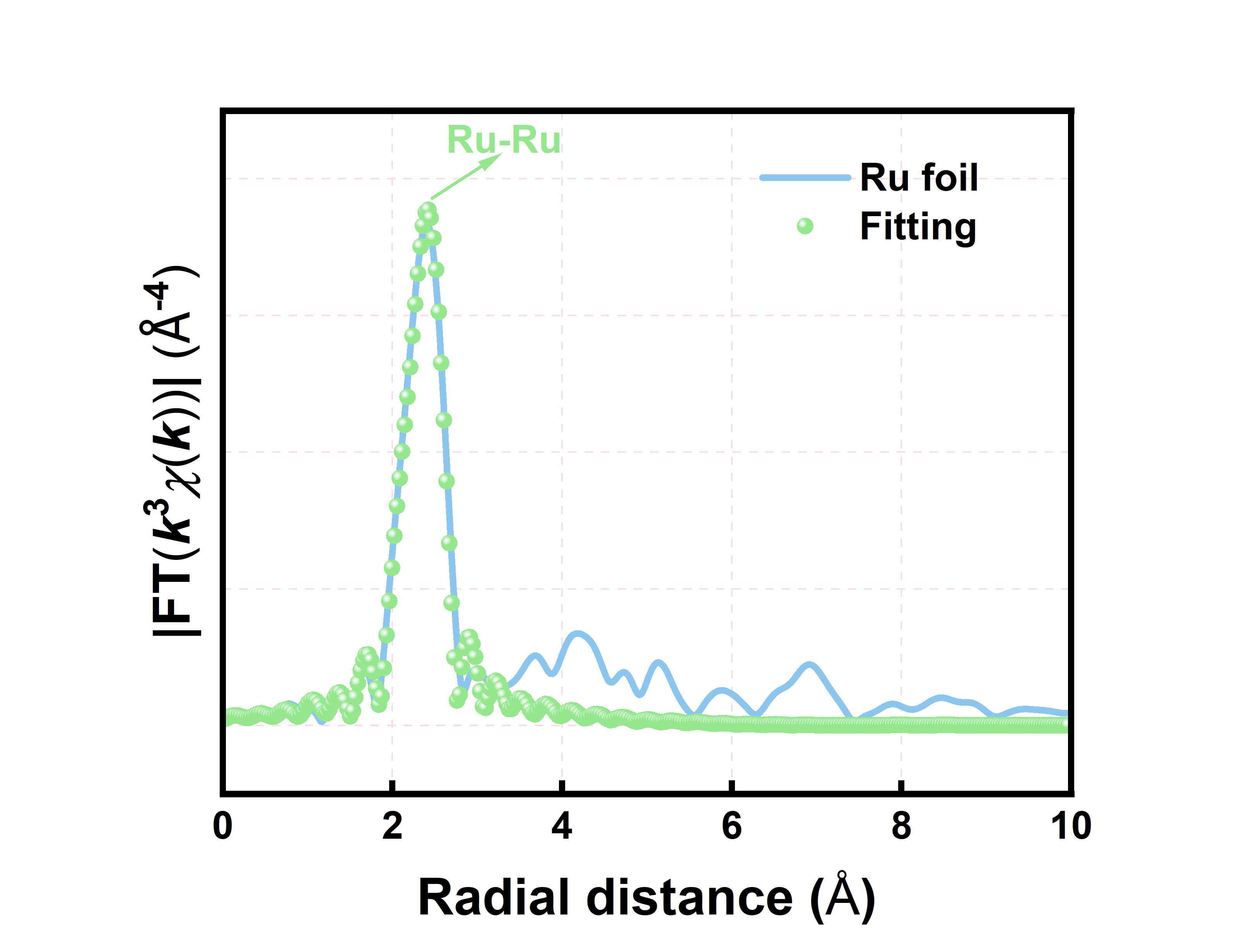
**

**Figure S8. EXAFS fitting curve of Ru foil at the R space.**

**
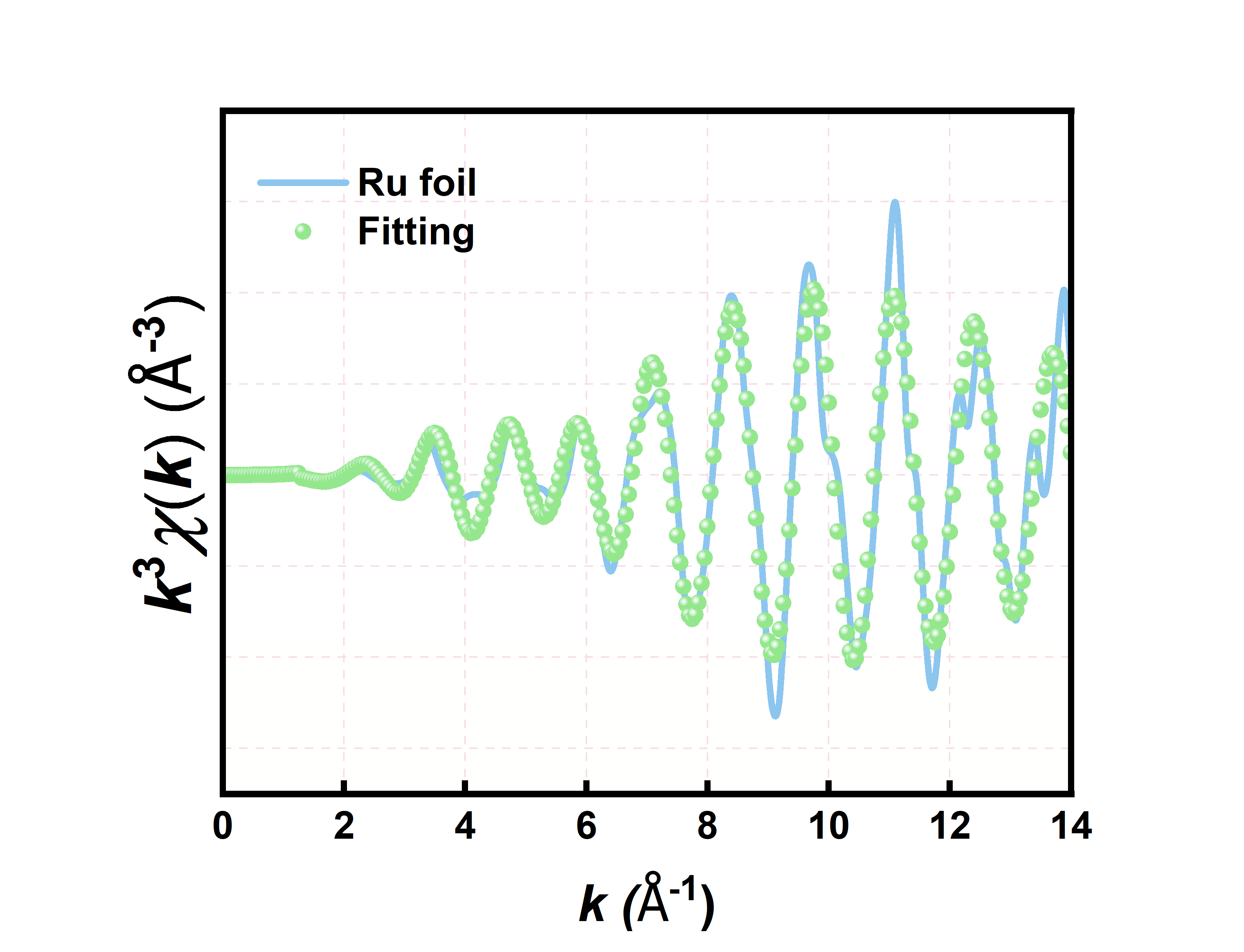
**

**Figure S9. EXAFS fitting curve of Ru foil at the k space.**

**
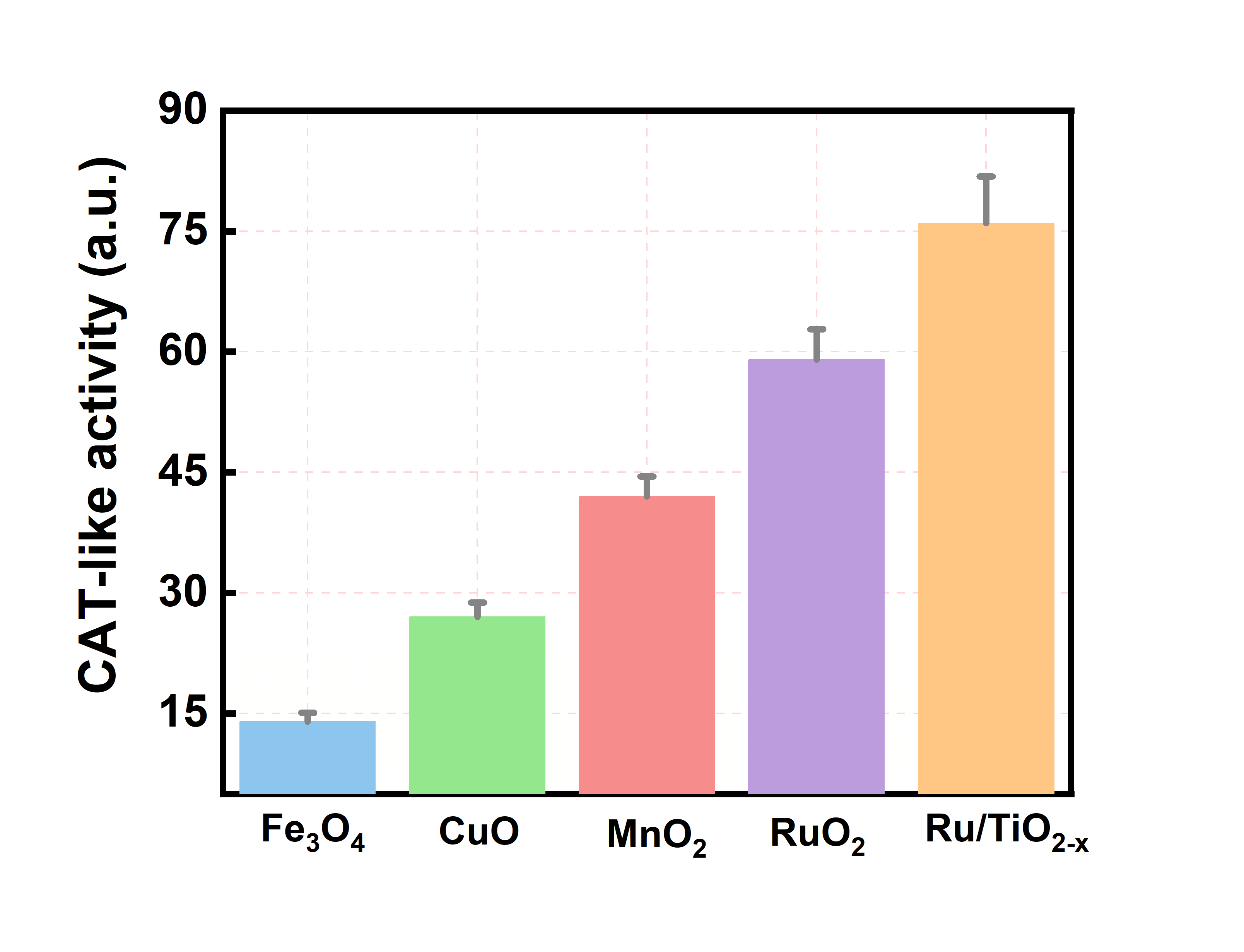
**

**Figure S10. The CAT-like activity of different nanozymes.**


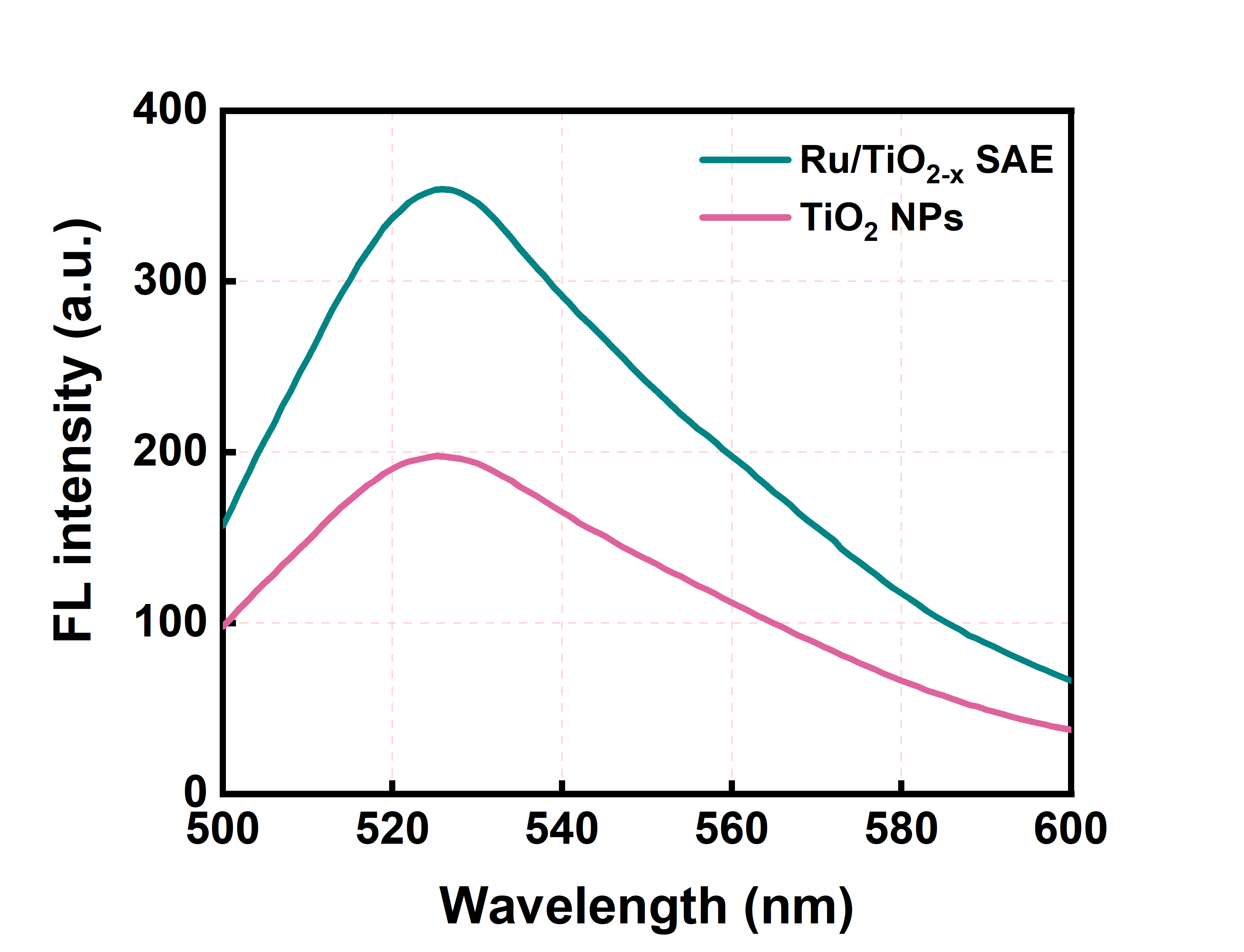


**Figure S11.** Fluorescence spectra of different formulations plus H_2_O_2_ with SOSG as a probe.


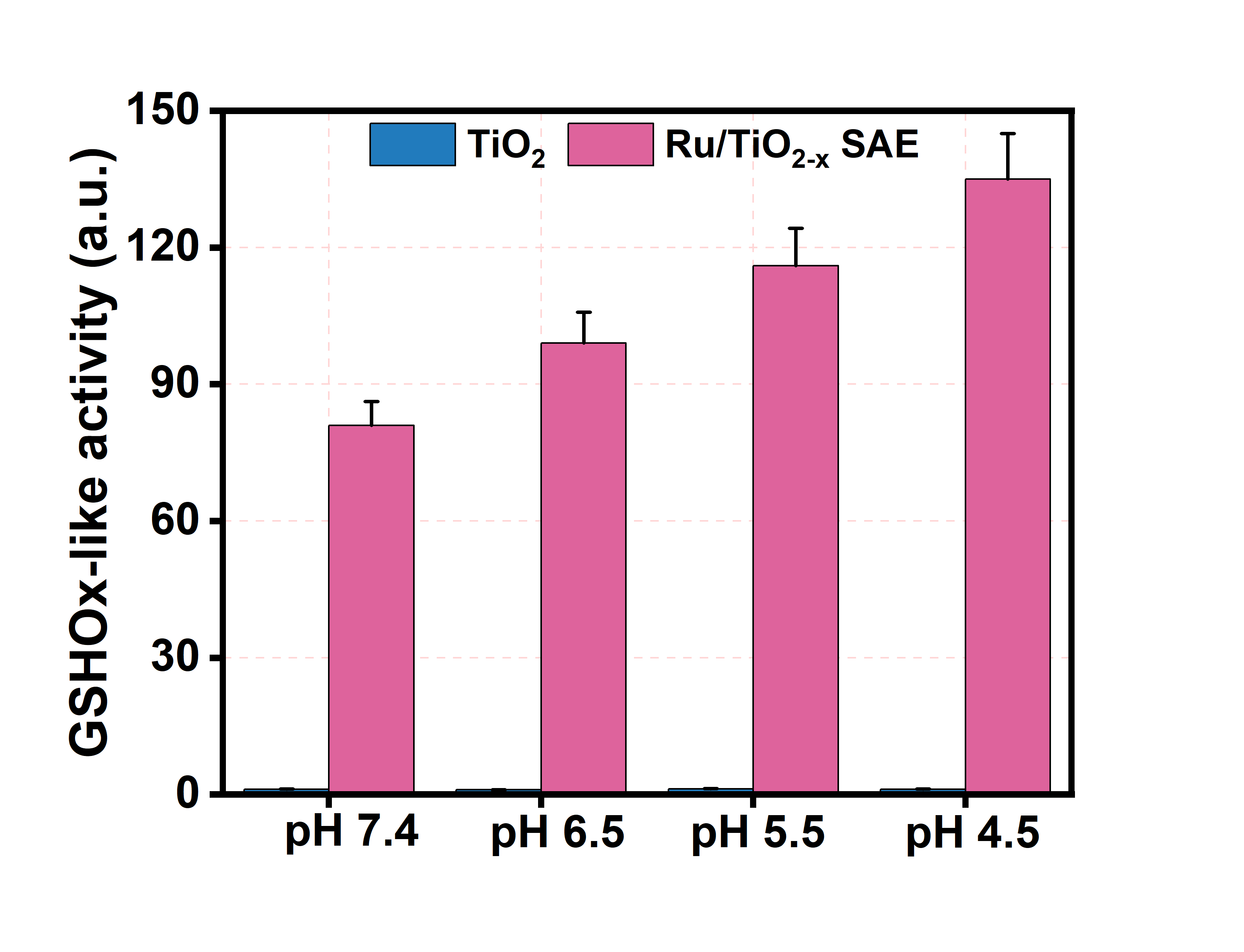


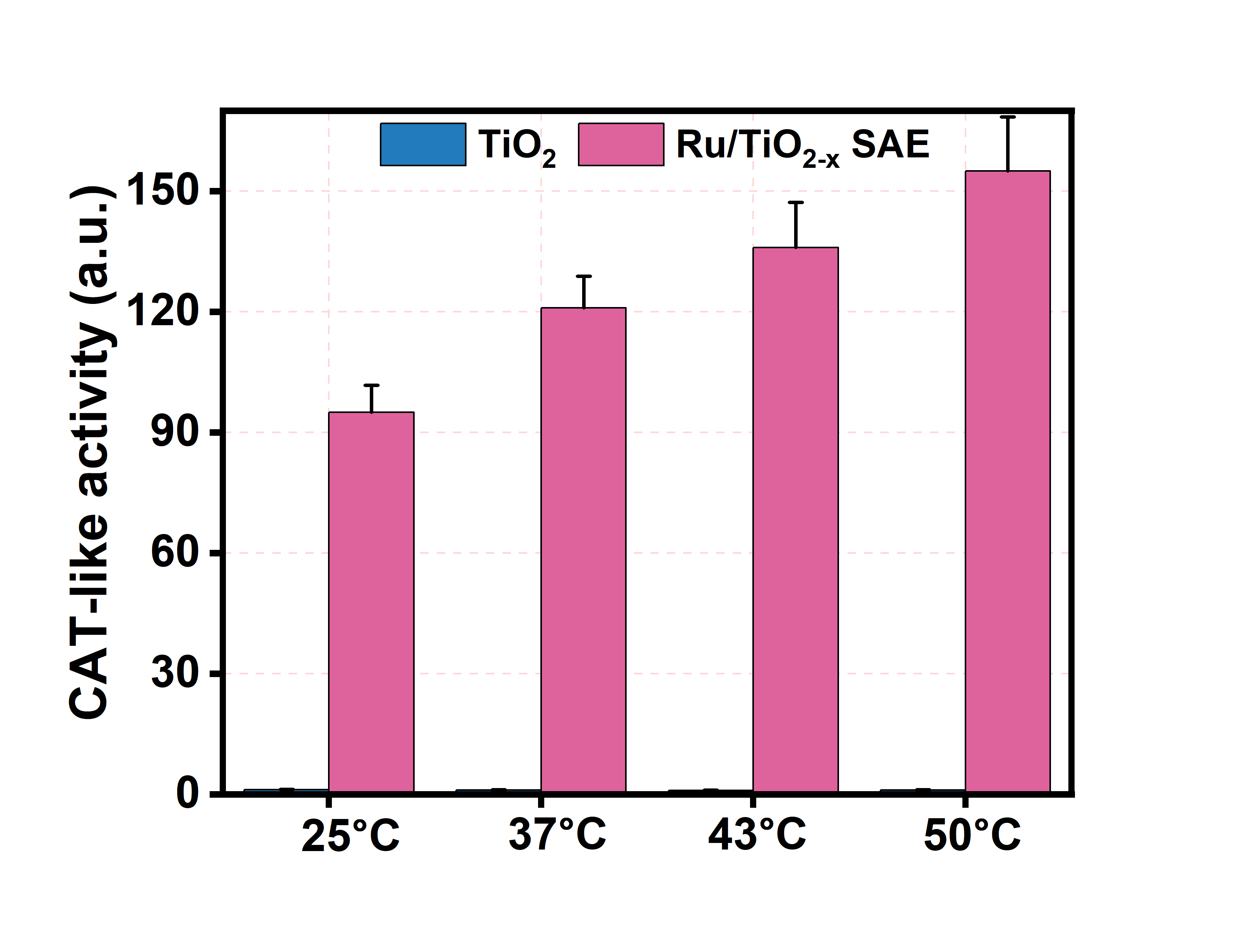


**Figure S12.** The CAT-like activity of Ru/TiO_2-x_ SAE in different pH and temperatures.


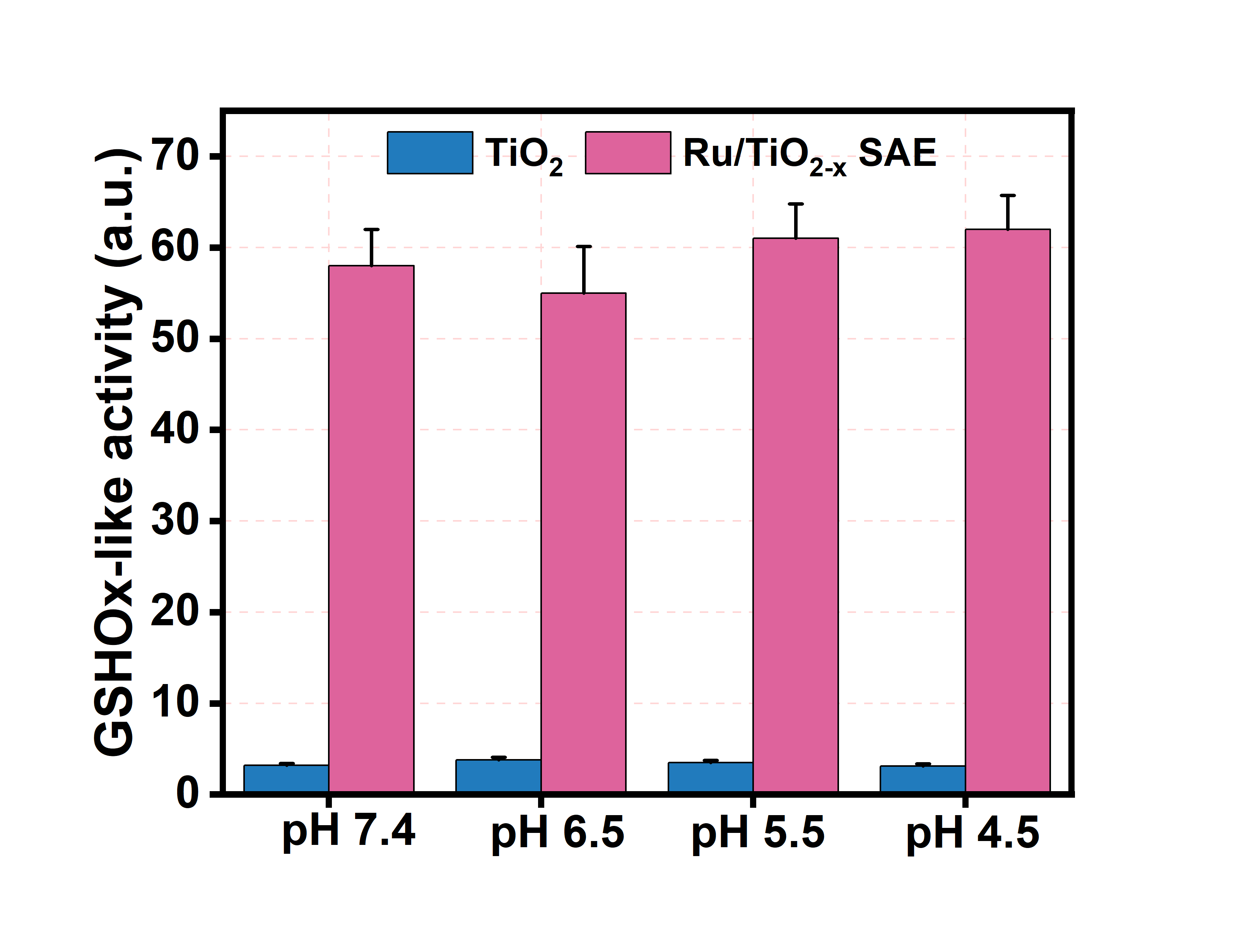


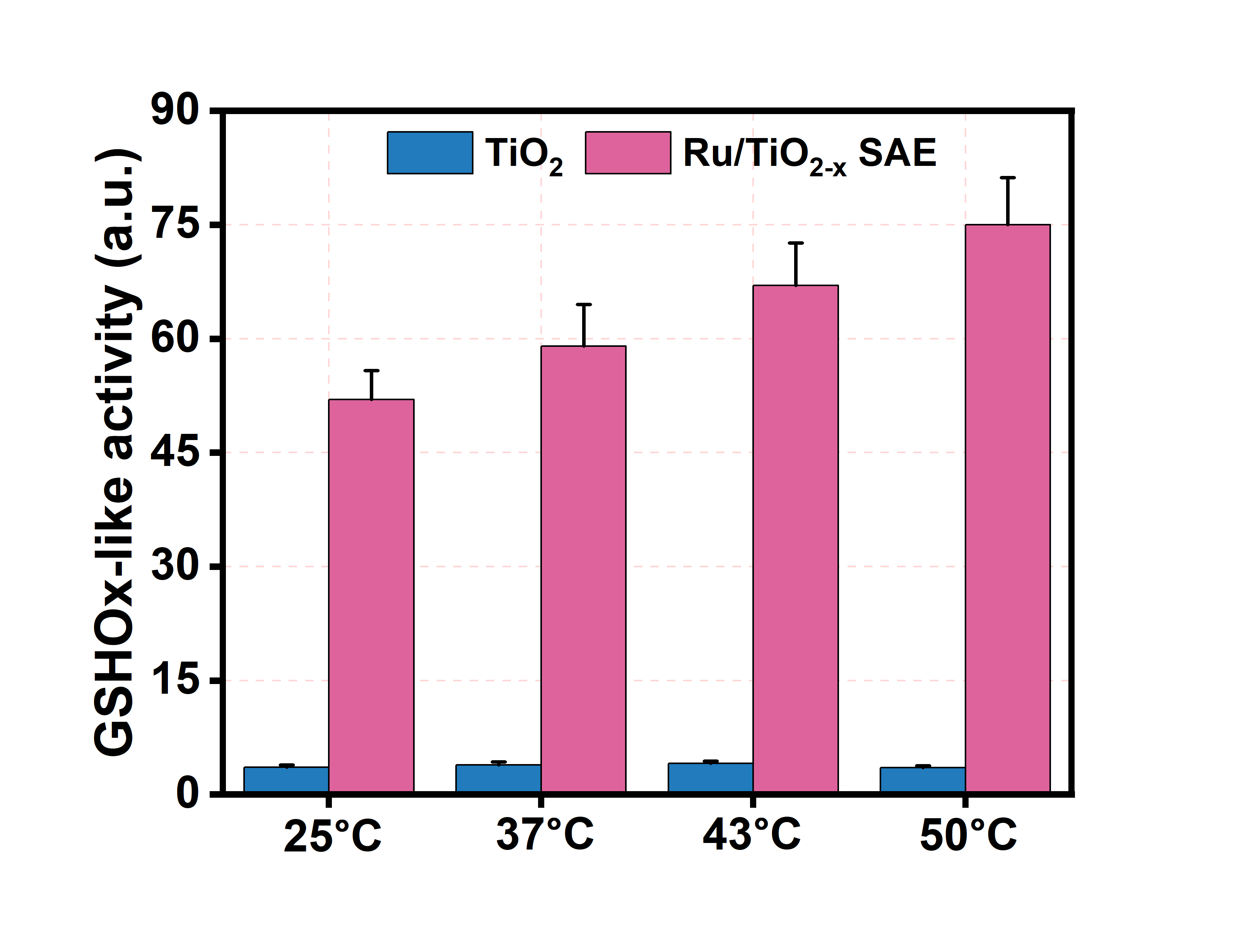


**Figure S13.** The GSHOX-like activity of Ru/TiO_2-x_ SAE in different pH and temperatures.

**
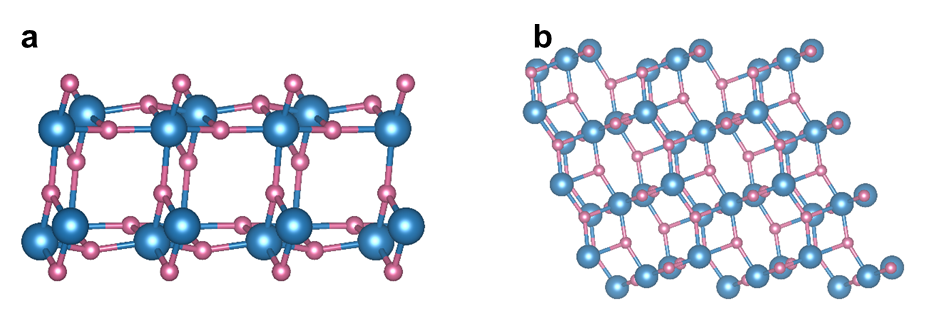
**

**Figure S14 Geometrically optimized structures of TiO_2_ NPs.** (a) Side view, (b) Top view.

**
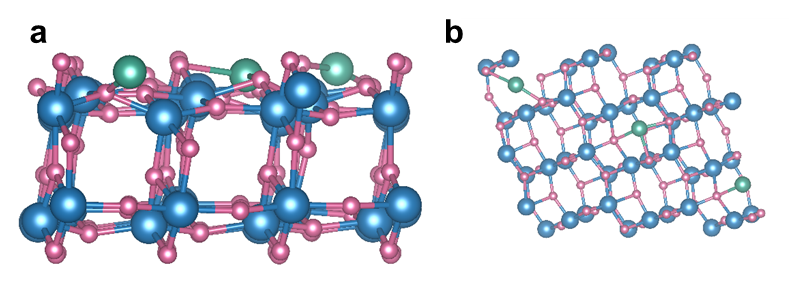
**

**Figure S15. Geometrically optimized structures of Ru/TiO_2-x_ SAE.** (a) Side view, (b) Top view.


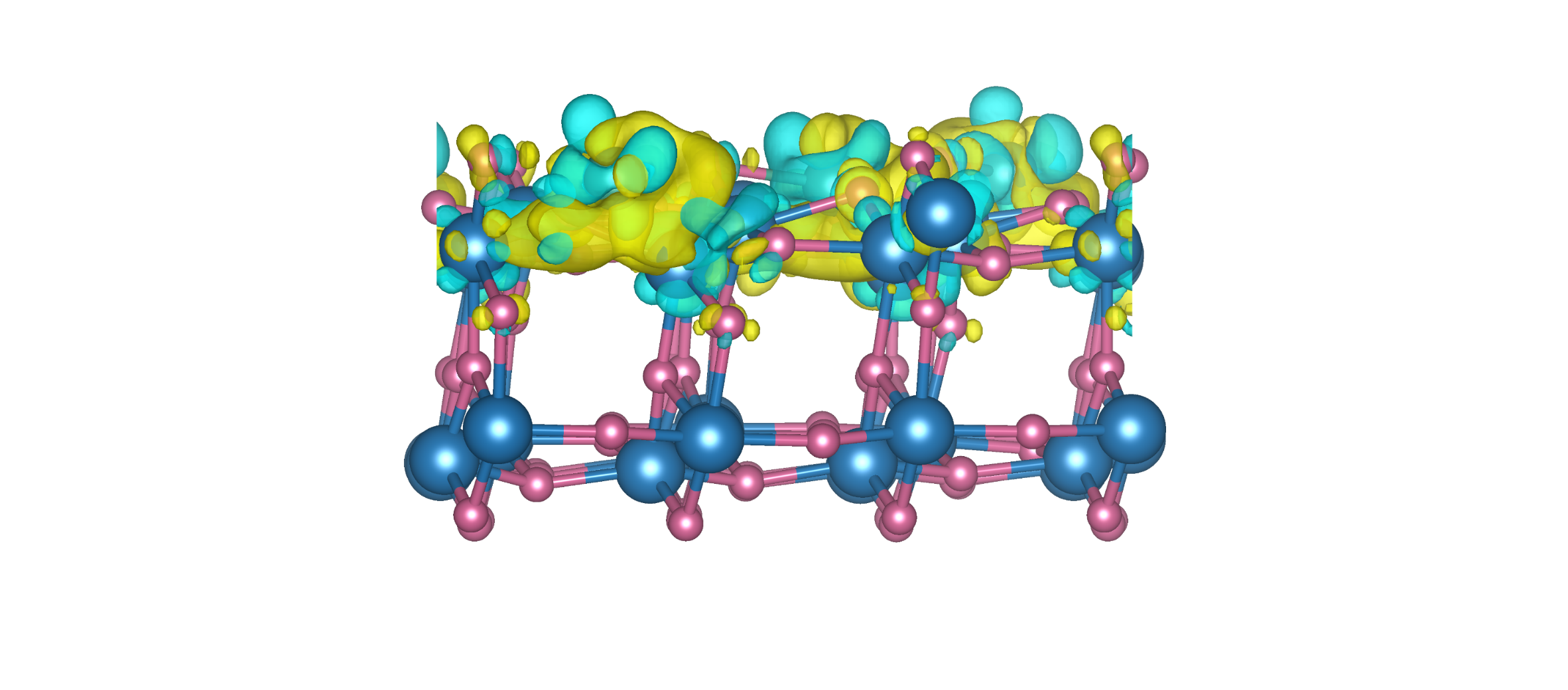


**Figure S16. The local charge density difference plot of the Ru/TiO_2-x_ SAE.** Yellow: charge accumulation; Cyan: charge depletion.

**
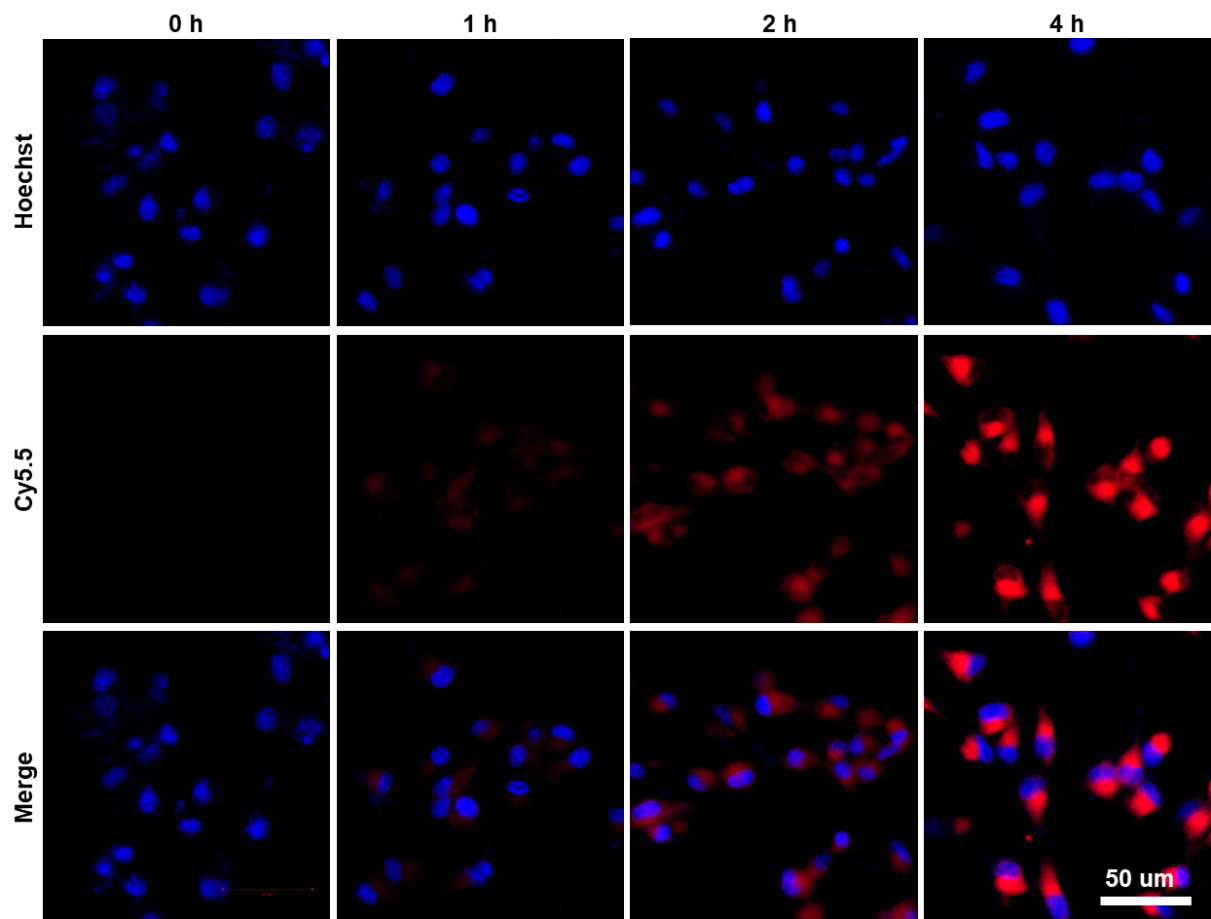
**

**Figure S17. The CLSM images of GL261 cells treated with Cy5.5-labeled Ru/TiO_2-x_ SAE.**

**
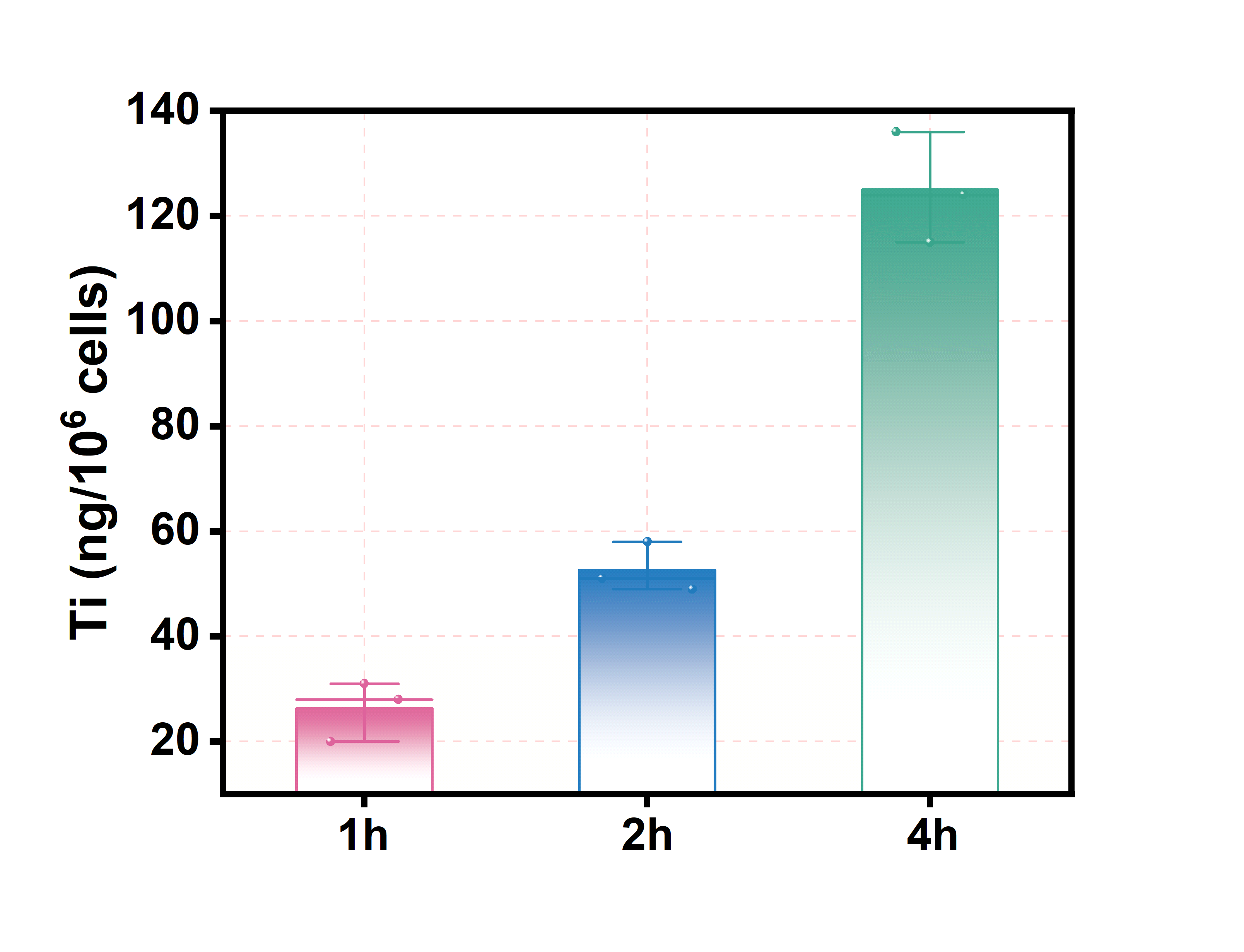
**

**Figure S18. The cellular uptake of Ru/TiO_2-x_ SAE using an ICP-MS.**

**
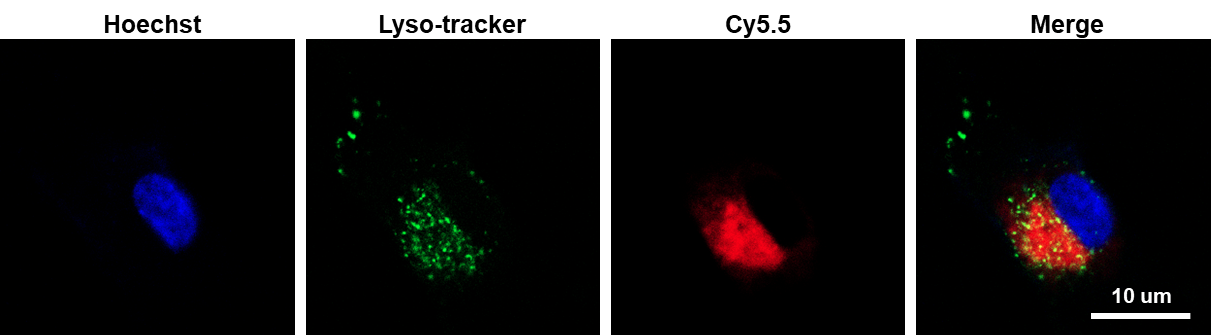
**

**Figure S19. The confocal images showed the colocalization of Ru/TiO_2-x_ SAE with the lysosomes of GL261 cells.**

**
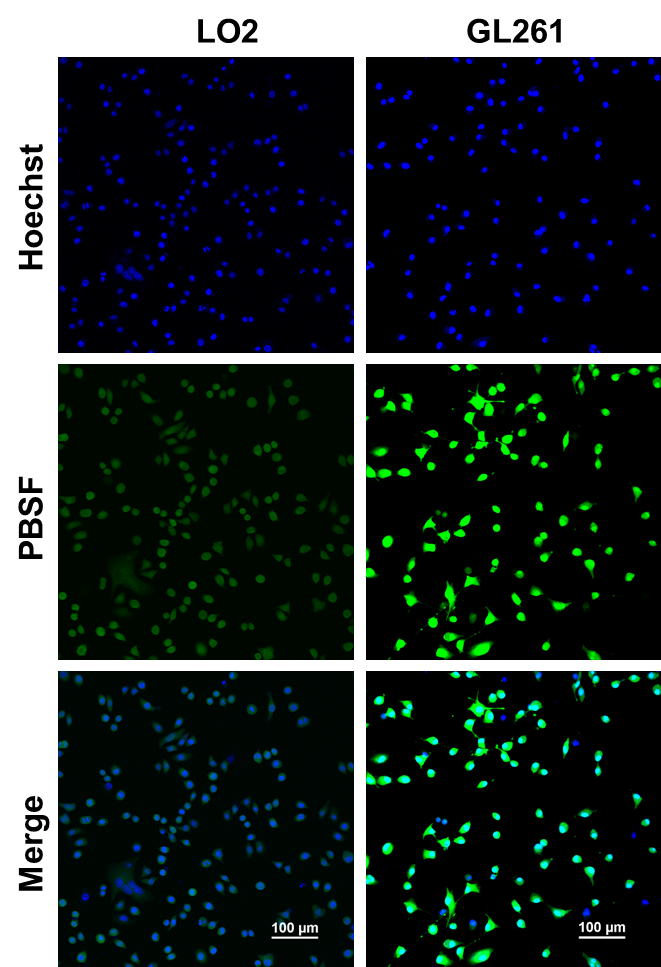
**

**Figure S20. CLSM images of LO2 and GL261 cells stained with H_2_O_2_ probe pentafluorobenzenesulfonyl fluorescein (PBSF).**

**
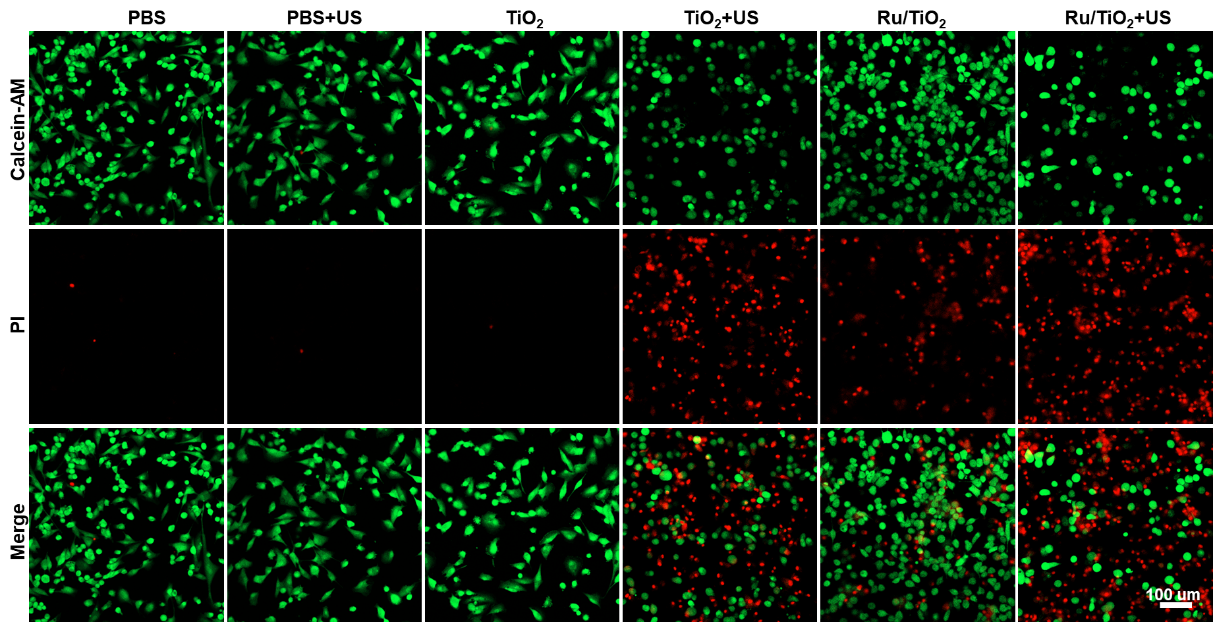
**

**Figure 21. The CLSM images of live/dead co-staining for GL261 cells following treatment with various formulation.**

**
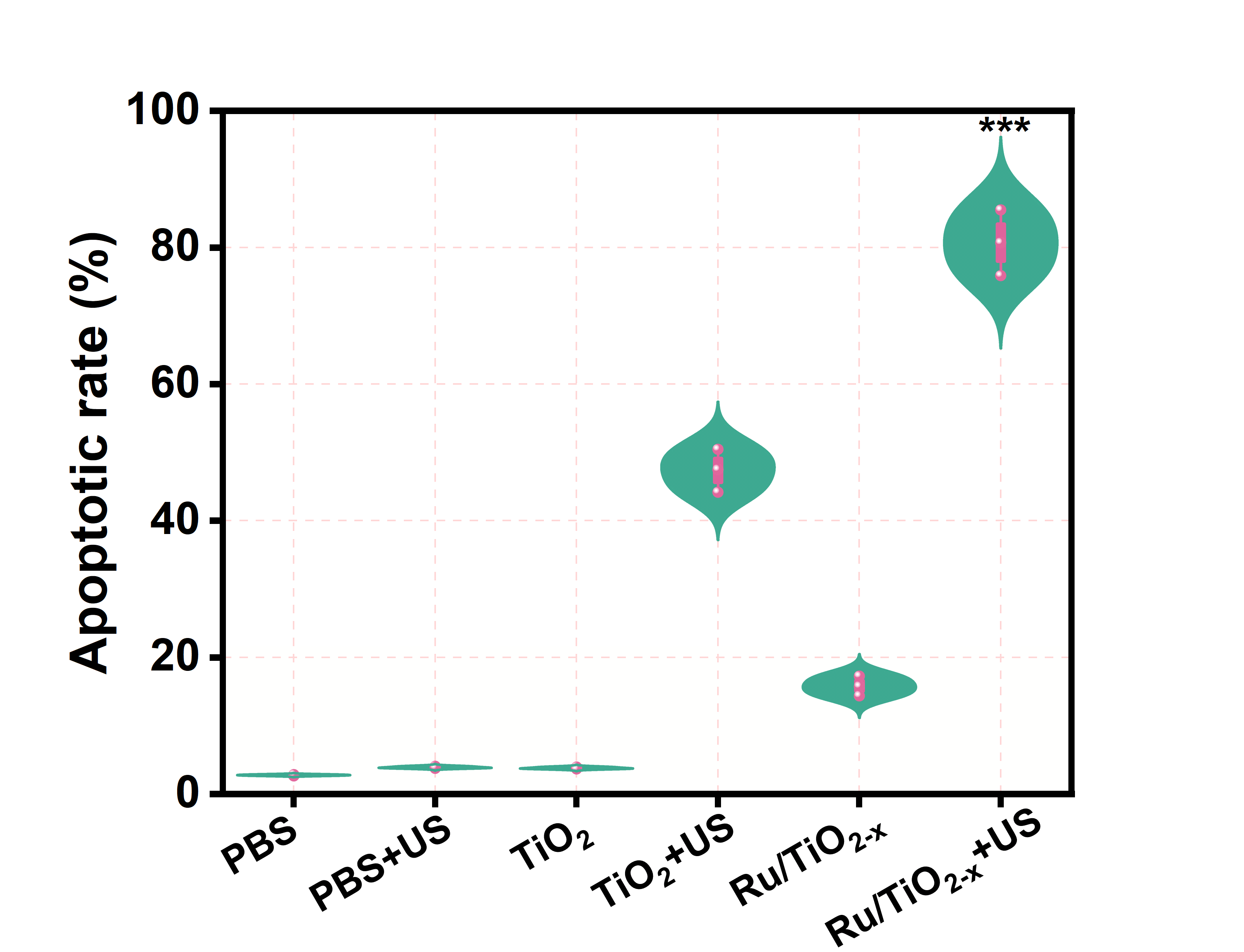
**

**Figure S22. Flow cytometry analysis of GL261 cells death rate following treatment with various formulations.**

**
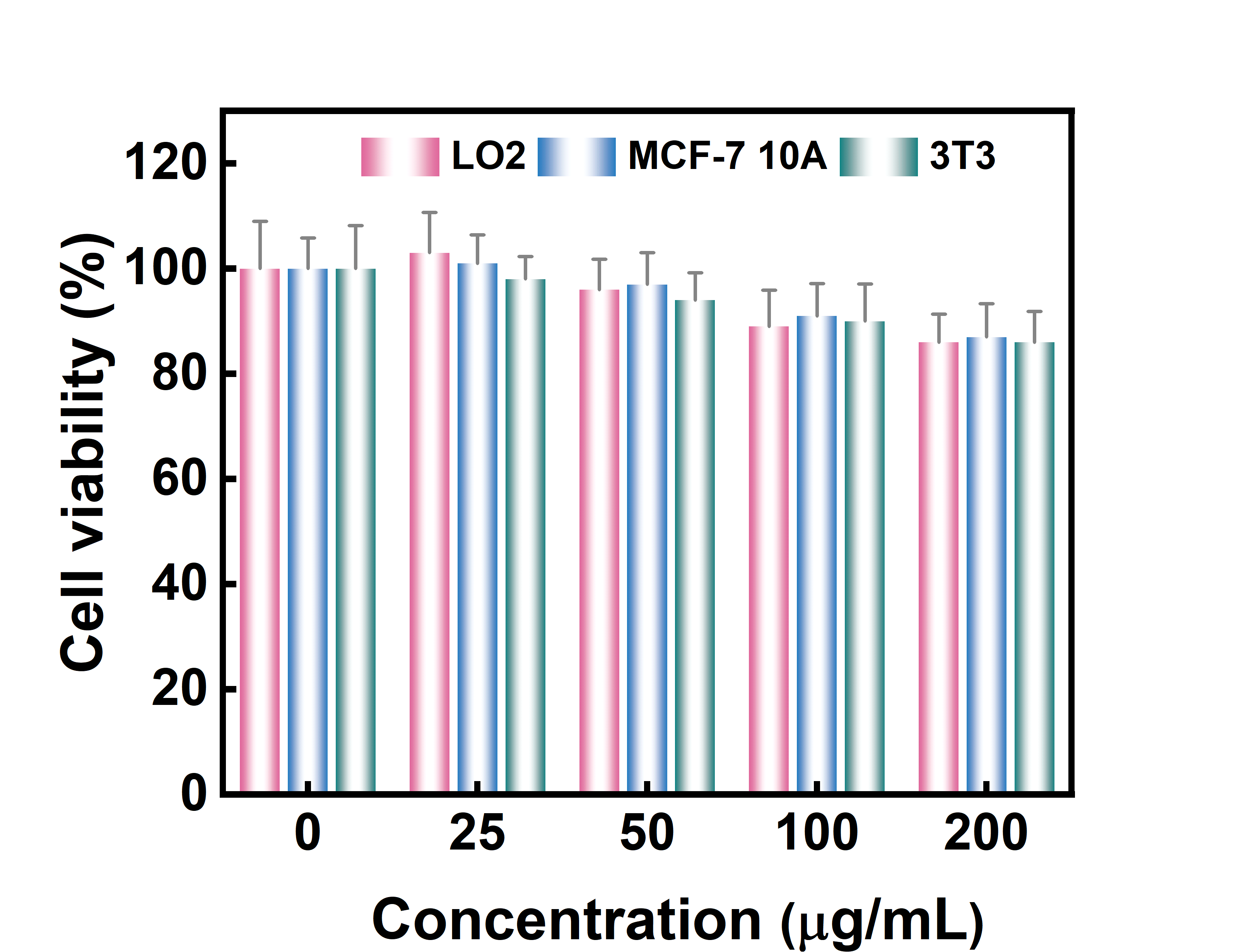
**

**Figure S23. The anti-proliferation effect of Ru/TiO_2-x_ SAE on different normal cells.**

**
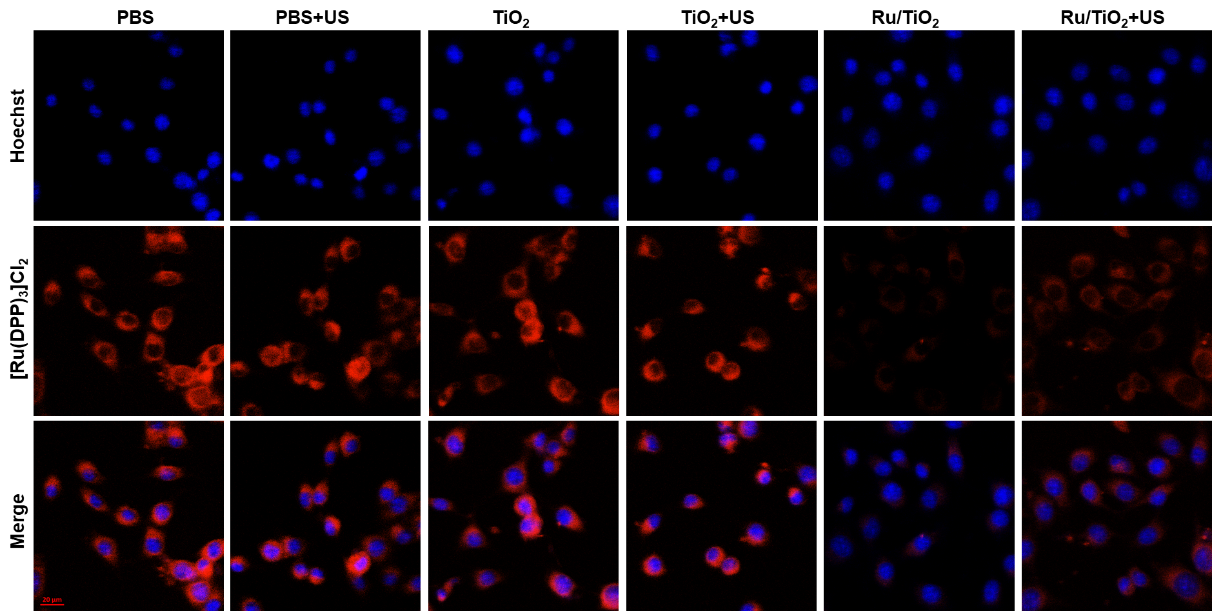
**

**Figure S24. CLSM images of GL261 cells stained with Ru(DPP)_3_Cl_2_ following various formulations.**

**
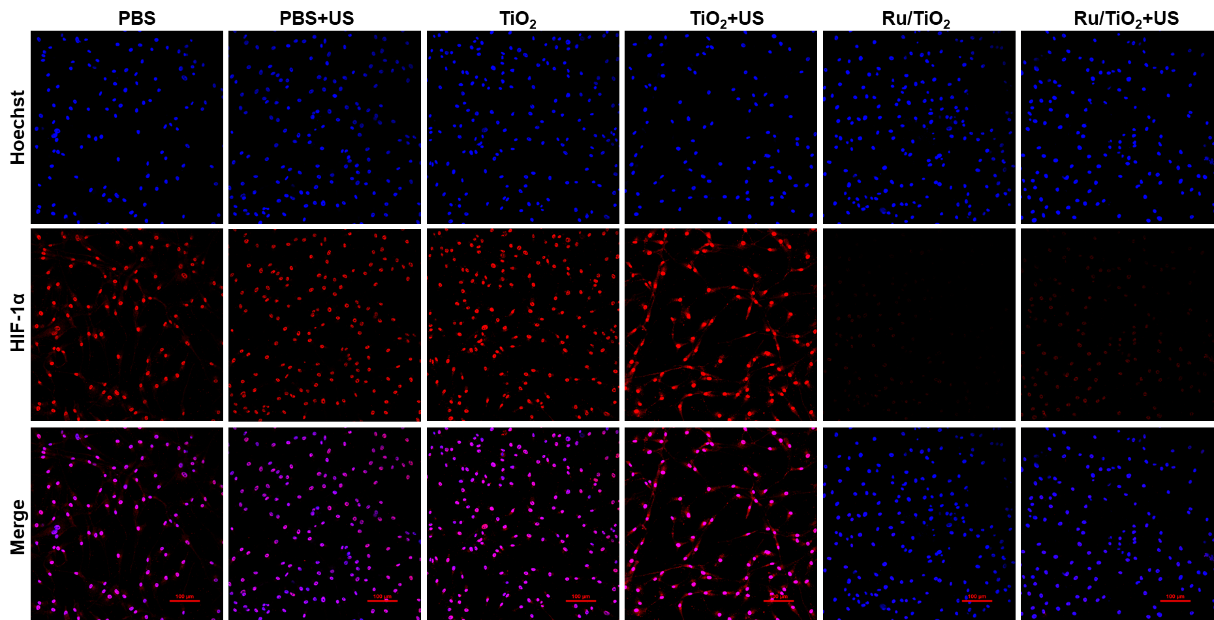
**

**Figure S25. IF images of tumor cells stained with HIF-1α following different treatments.**


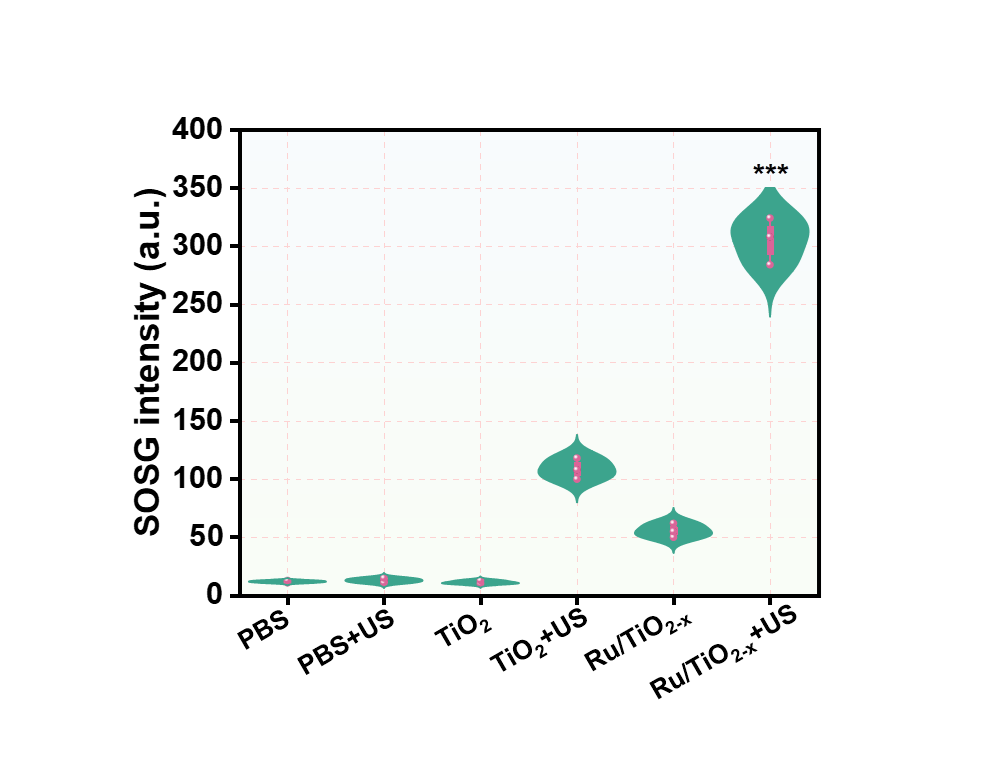


**Figure S26. 18 SOSG fluorescence of tumor cells exposed to various formulations**.


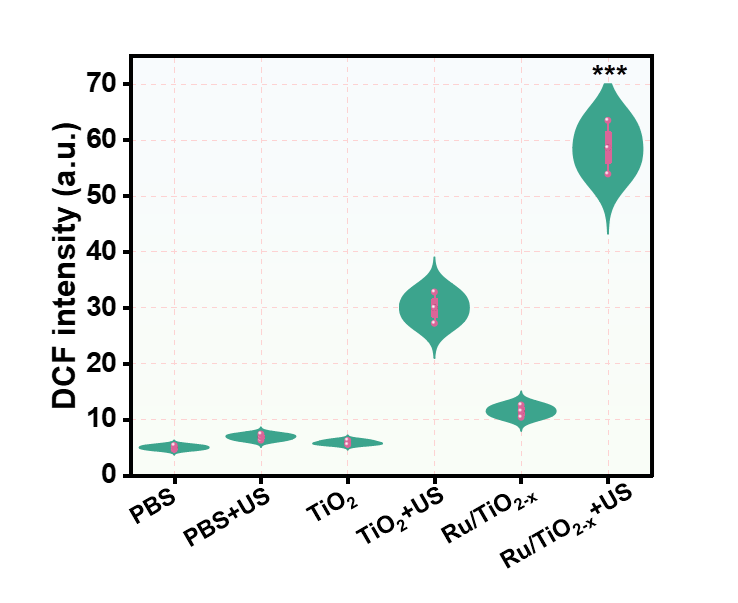


**Figure S27. Flow cytometry analysis of ROS intensity in GL261 cells after treatment with various formulations.**

**
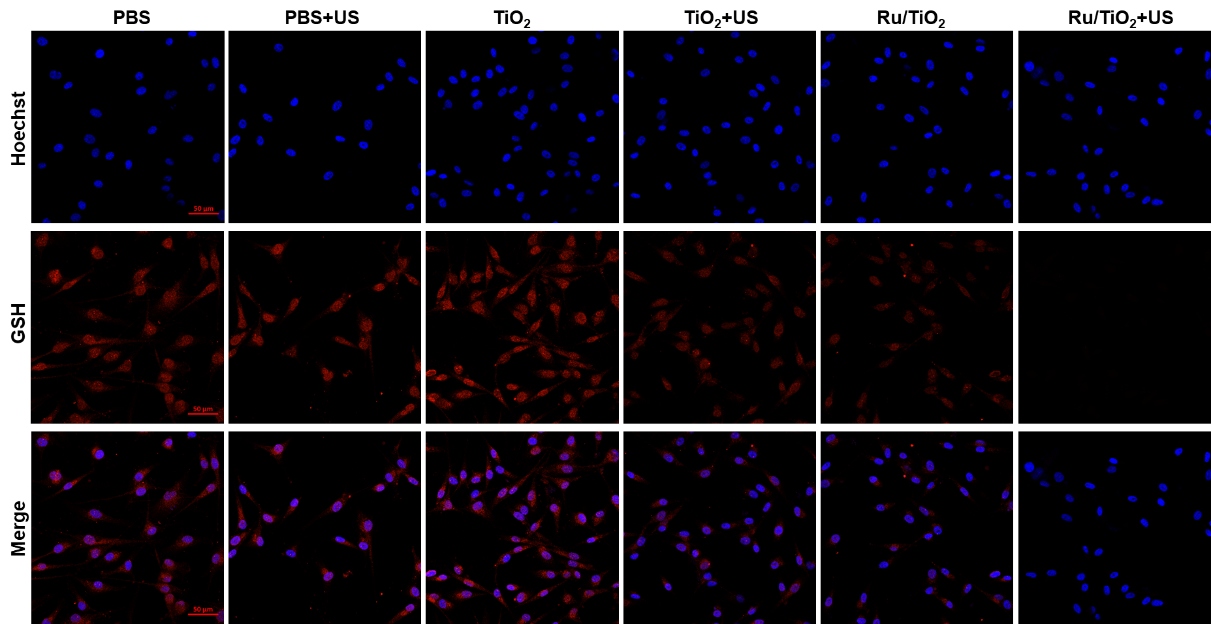
**

**Figure S28. CLSM images of GSH content in GL261 cells following different treatments.**

**
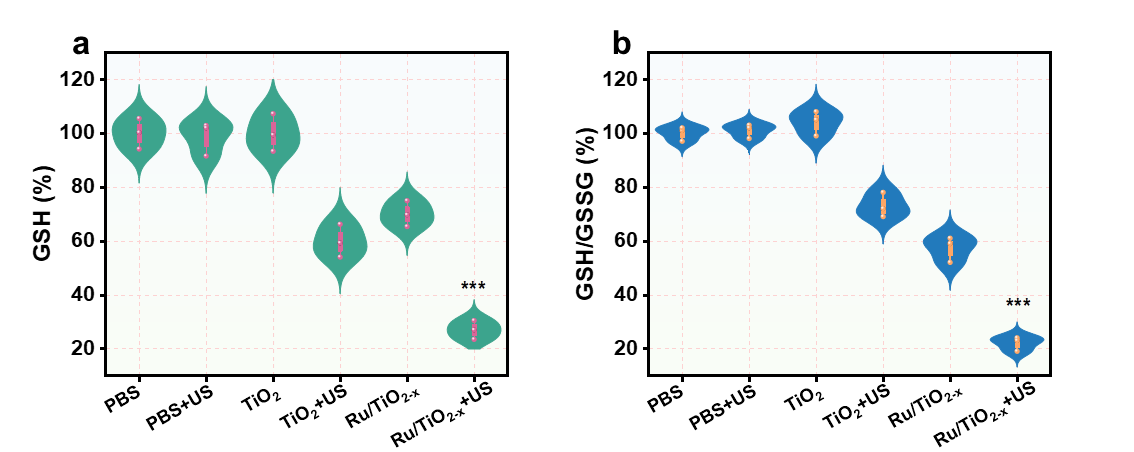
**

**Figure S29. The levels of cellular GSH after various treatments were determined using a DTNB assay kit. (a) GSH level, (b) the rate of GSH and GSSG.**

**
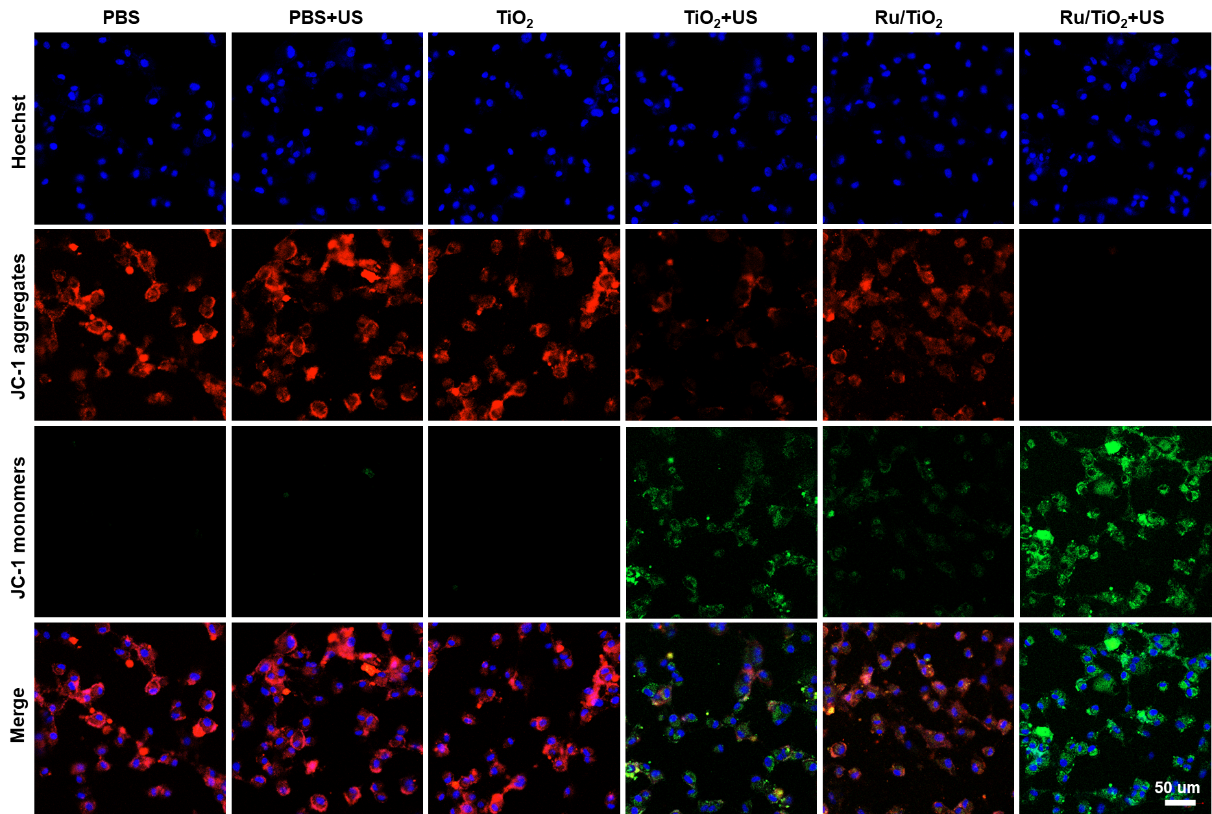
**

**Figure S30. CLSM images of GL261 cells measured with JC-1 kit following various treatments.**

**
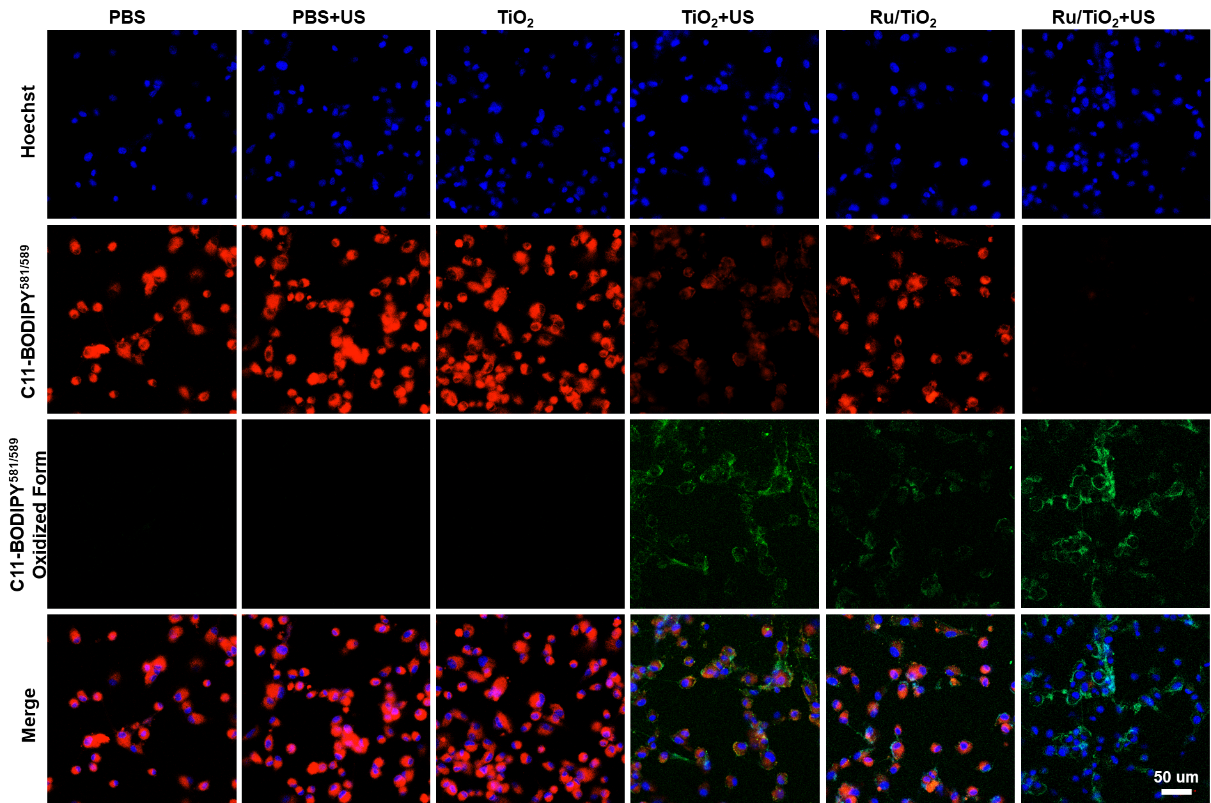
**

**Figure S31. Confocal images of tumor cells stained with C11-BODIPY^581/589^ following a 24-hour treatment with various formulations.**

**
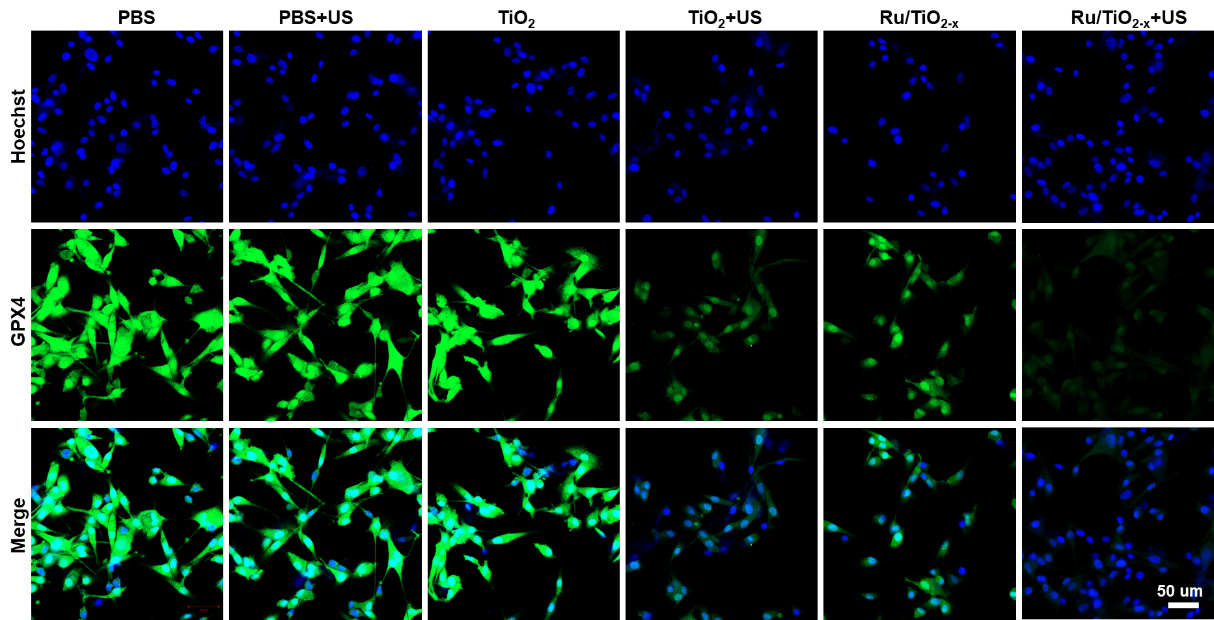
**

**Figure S32. CLSM images of GPX4 expression in cancer cells after different treatments.**


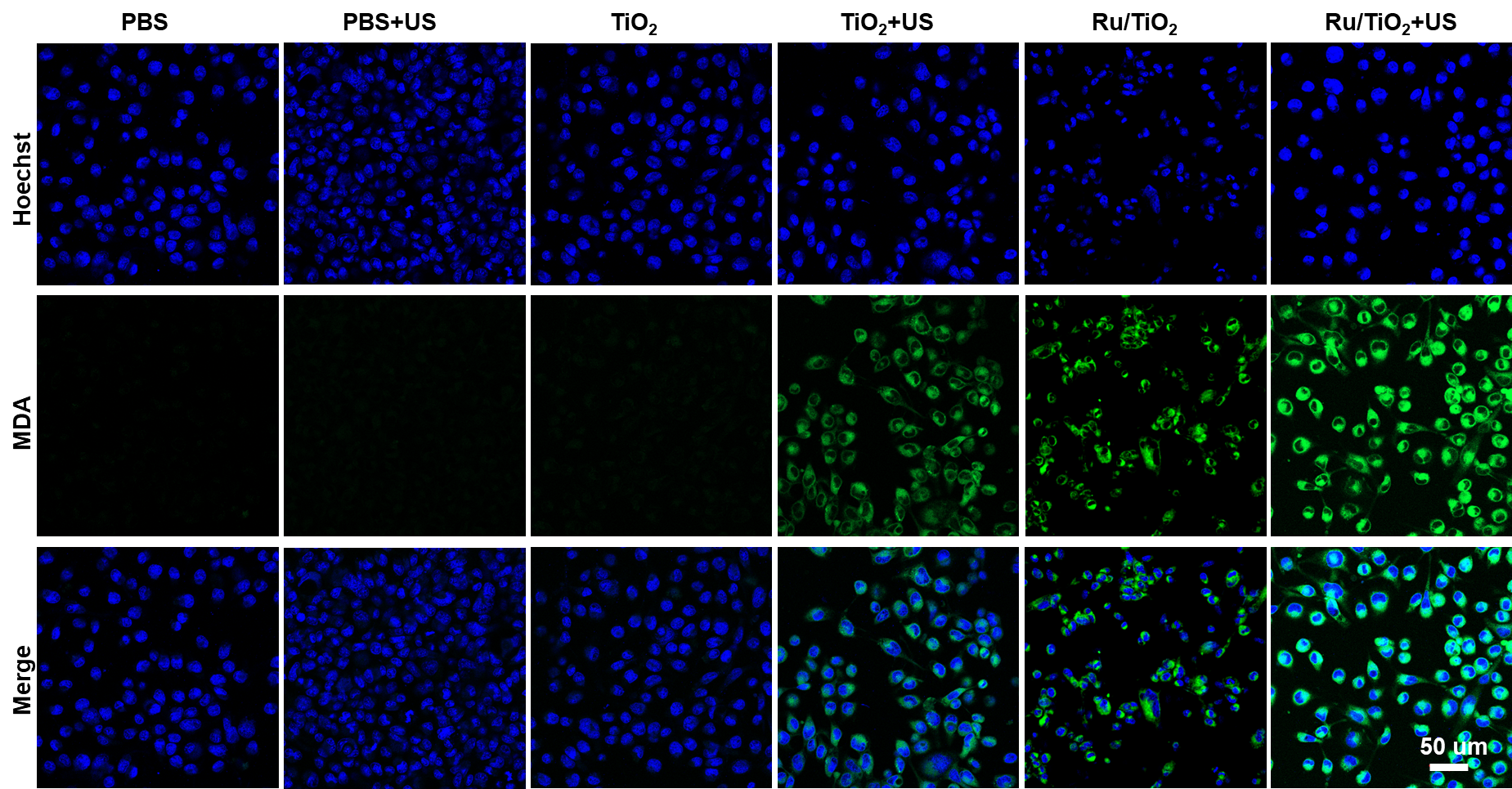


**Figure S33. Confocal images of GL261 cells stained with MDA kit following different treatments.**


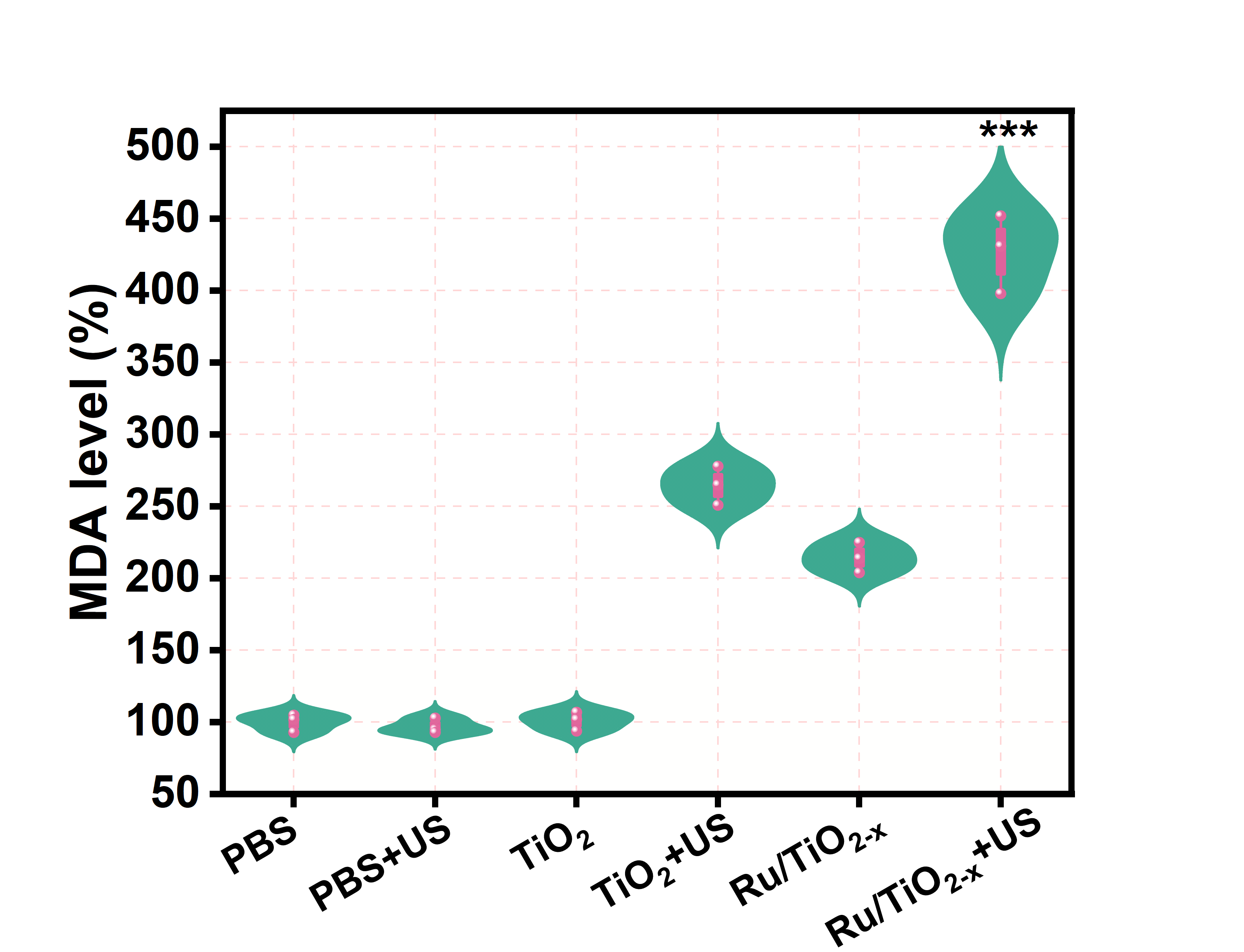


**Figure S34. Quantitative analysis of MDA levels using the thiobarbituric acid (TBA) assay through ultraviolet-visible spectrophotometry following various treatments.**

**
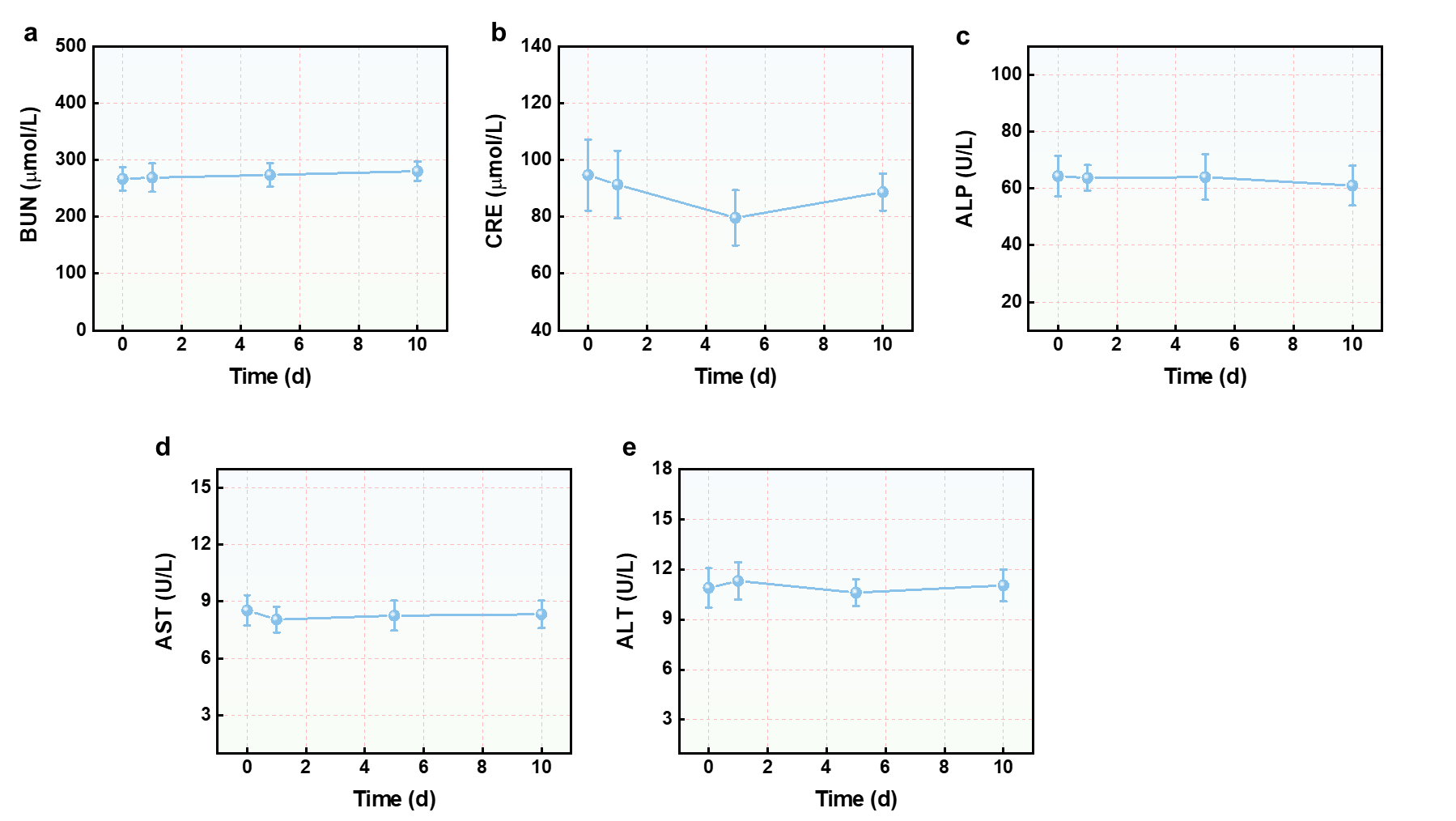
**

**Figure S35. Blood biochemistry analysis of mice after intravenous injection with Ru/TiO_2-x_ SAE at different time points.** a) Blood urea nitrogen (BUN). b) Creatinine (CRE). c) Alkaline phosphatase (ALP) d) Aspartate aminotransferase (AST). (e) Alanine transaminase (ALT) levels at days 1, 5, and 10.

**
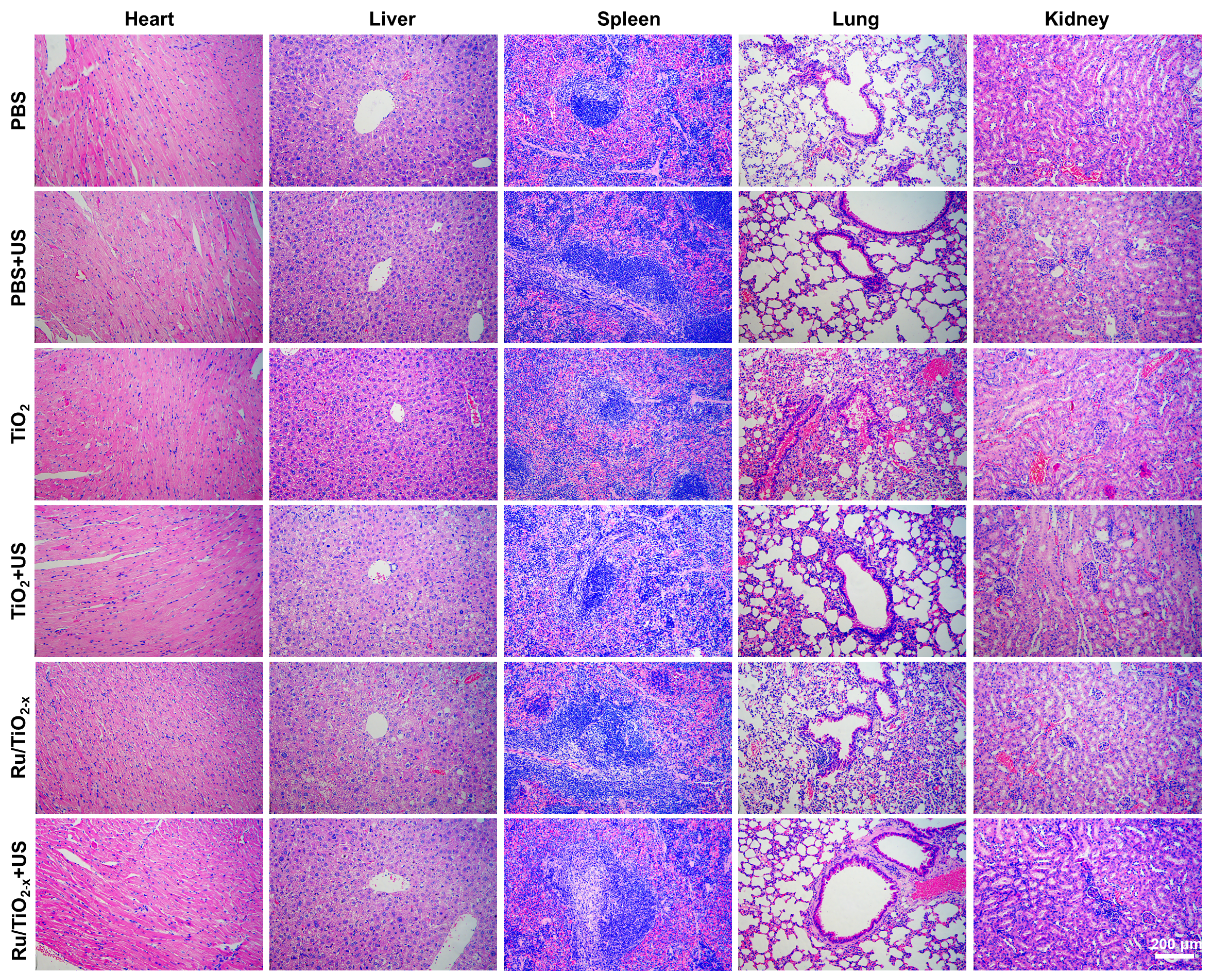
**

**Figure S36. H&E-stained images of major organs harvested from different groups of mice at 10 days post-treatment.**

**
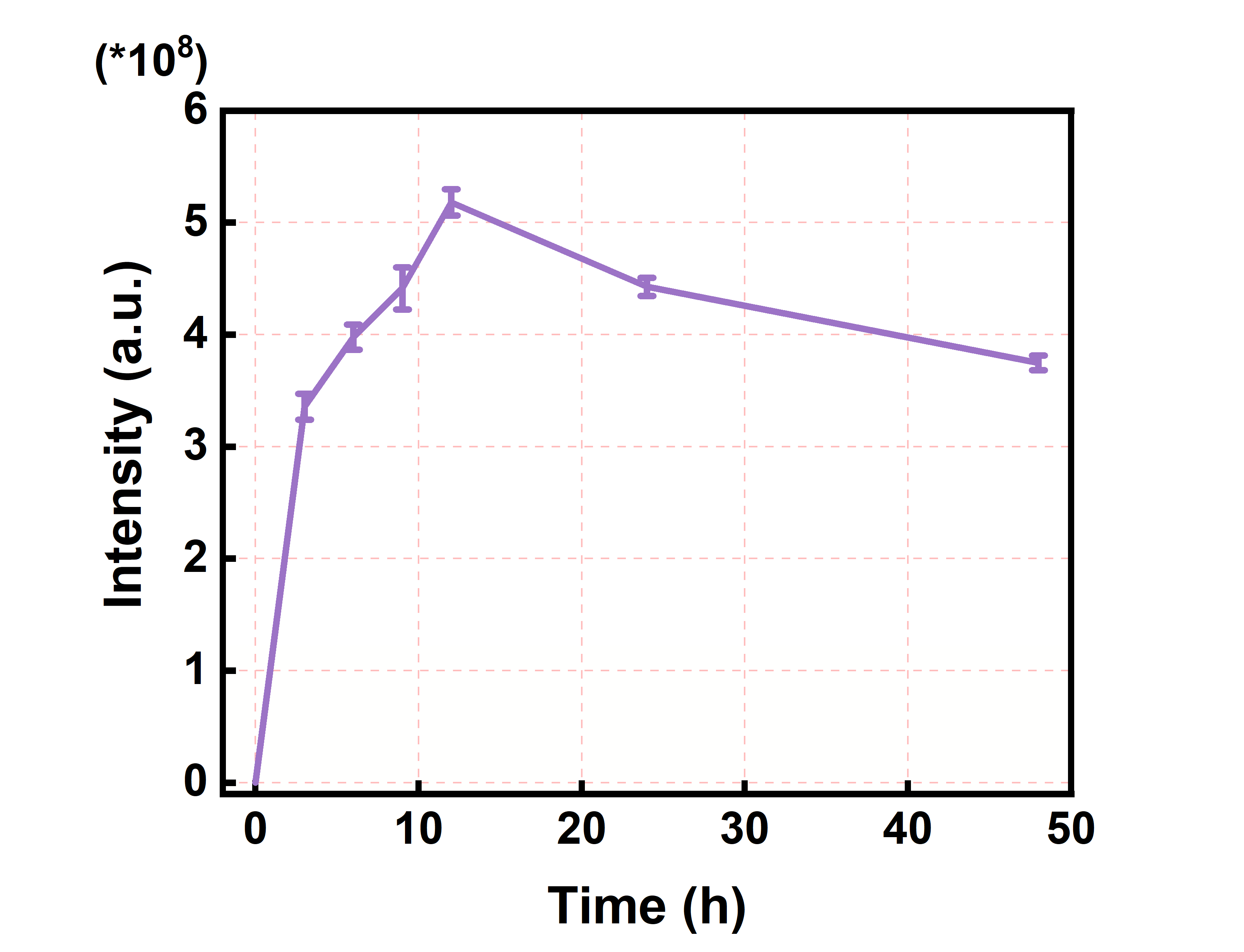
**

**Figure S37. The corresponding quantification of GL261 tumor-bearing mice at various time points post-injection with Cy5.5-labeled Ru/TiO_2-x_ SAE.**

**
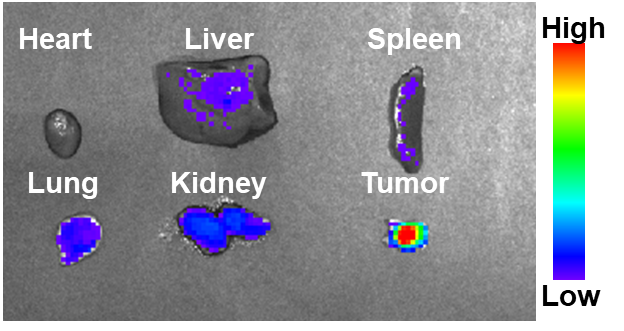
**

**Figure S38. Fluorescence images of the major organs (heart, liver, spleen, lung, kidney, brain) and tumor taken from the tumor bearing mice at 24 h after intravenous injection.**

**
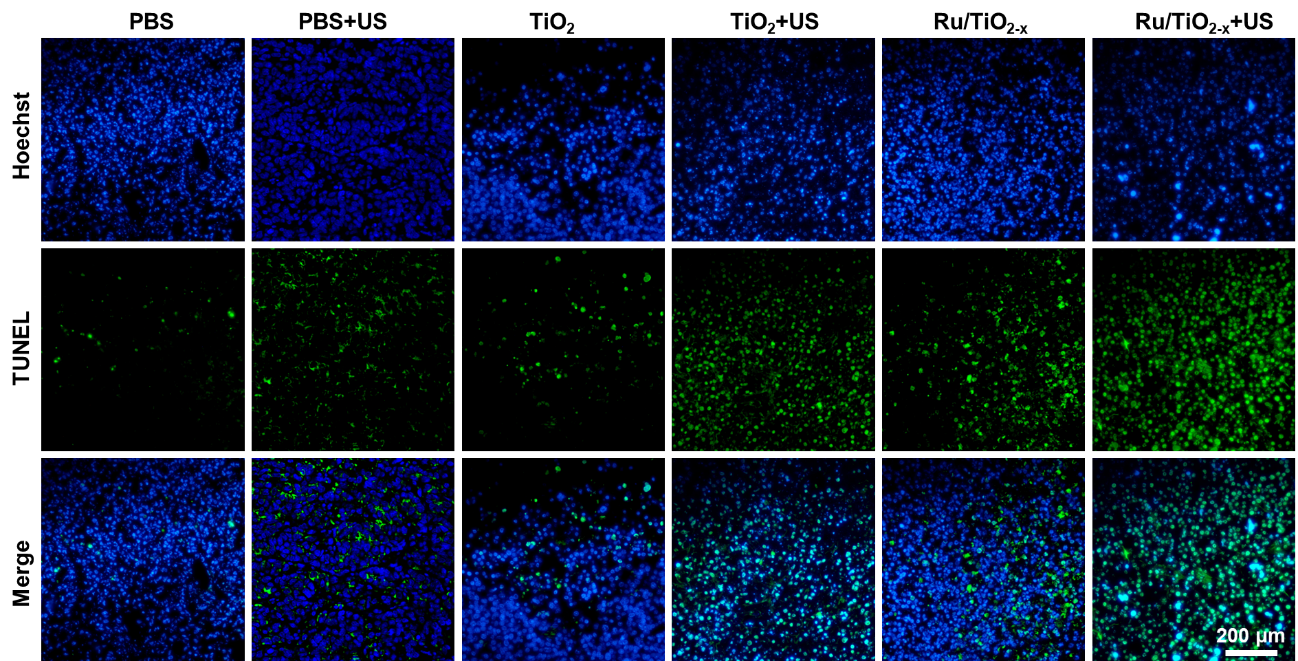
**

**Figure S39. TUNEL staining of tumor slices from various groups following different formulations.**

**
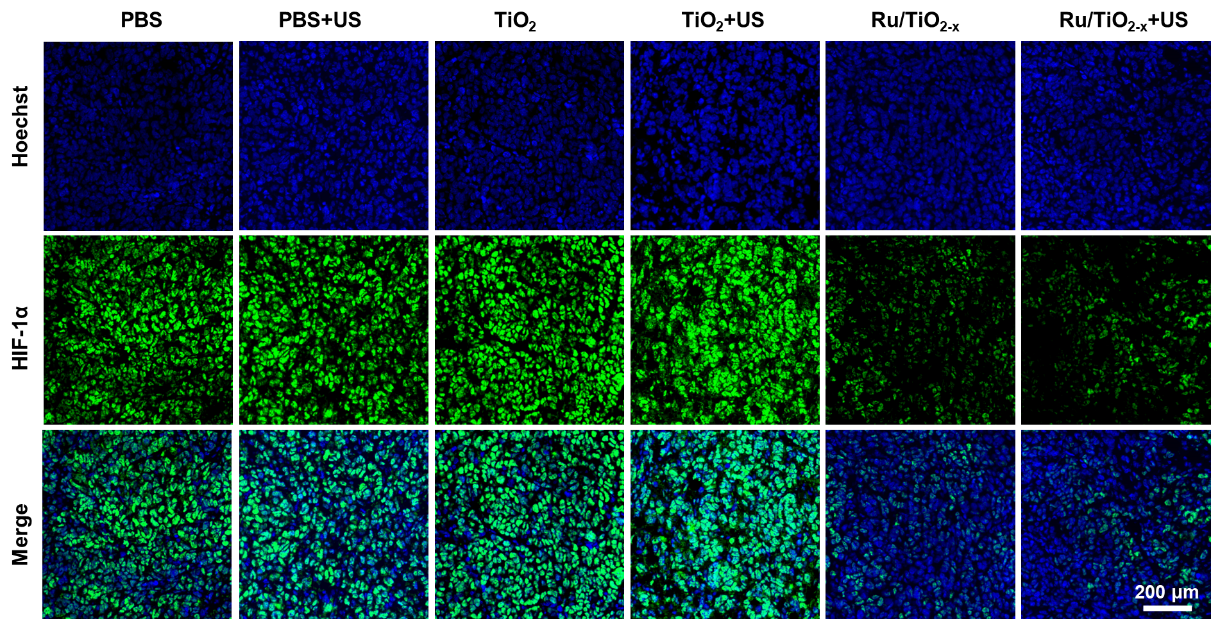
**

**Figure S40. Immunofluorescence analysis of HIF-1α protein expression in tumor treated with different formulation.**

**
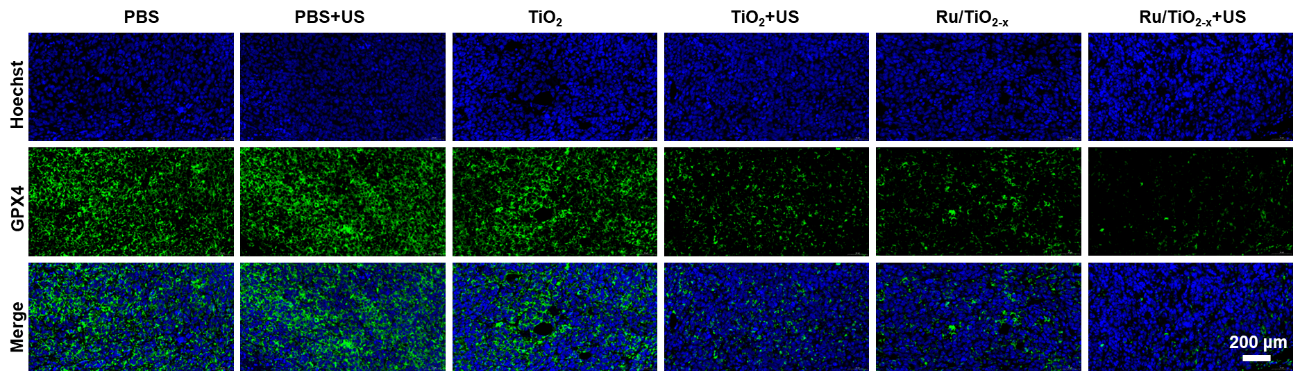
**

**Figure S41. Immunofluorescence analysis of GPX4 protein expression in tumor treated with different formulation.**

**Table S1 EXAFS fitting parameters of various samples at the Ru**

**K-edge (S0^2^ = 0.92).**

| **Ru K-edge (Ѕ_0_^2^=0.828)** | | | | | | |
| --- | --- | --- | --- | --- | --- | --- |
| **Ru foil** | **Ru-Ru** | **12.0*** | **2.673±0.003** | **0.0045** | **6.2** | **0.0114** |
| **RuCl_3_** | **Ru-Cl** | **6.1±0.7** | **2.339±0.019** | **0.0049** | **-2.2** | **0.0087** |
| **RuO_2_** | **Ru-O** | **6.2±0.4** | **1.971±0.007** | **0.0031** | **-0.4** | **0.0167** |
|  | **Ru-Ru** | **2.1±0.2** | **3.105±0.007** | **0.0088** | **-4.0** |  |
|  | **Ru-Ru** | **7.6±0.3** | **3.538±0.005** |  |  |  |
| **Ru/TiO_2-x_** | **Ru-O** | **4.1±0.3** | **2.008±0.023** | **0.0040** | **-12.7** | **0.0088** |

*^a^CN*, coordination number; *^b^R*, the distance between absorber and backscatter atoms; *^c^σ*^2^, the Debye Waller factor value; ^d^*ΔE*_0_, inner potential correction to account for the difference in the inner potential between the sample and the reference compound; *R* factor indicates the goodness of the fit. *S*0^2^ was fixed to 0.727, 0.823 and 0.828, according to the experimental EXAFS fit of Ru foil by fixing *CN* as the known crystallographic value. * This value was fixed during EXAFS fitting, based on the known structure of Ru. Fitting conditions: *k* range：1.0 - 9.0; *R* range: 1.0-2.0 (Ru/TiO_2-x_ SAE); fitting space: R space; *k*-weight = 3. A reasonable range of EXAFS fitting parameters: 0.800 < *Ѕ*_0_^2^ < 1.000; *CN >* 0; *σ*^2^ > 0 Å^2^; |Δ*E*_0_| < 15 eV; *R* factor < 0.02.
